# Synthesis and Biological Evaluation of Chalconesulfonamides: En Route to Proapoptotic Agents with Antiestrogenic Potency

**DOI:** 10.3390/ph17010032

**Published:** 2023-12-25

**Authors:** Stepan K. Krymov, Diana I. Salnikova, Lyubov G. Dezhenkova, Fedor B. Bogdanov, Alexander A. Korlyukov, Alexander M. Scherbakov, Andrey E. Shchekotikhin

**Affiliations:** 1 Gause Institute of New Antibiotics, 11 B. Pirogovskaya Street, 119021 Moscow, Russia; krymov.s.k@gmail.com (S.K.K.); dezhenkovalg@yahoo.com (L.G.D.); 2 Department of Experimental Tumor Biology, Blokhin N. N. National Medical Research Center of Oncology, Kashirskoe sh. 24, 115522 Moscow, Russia; dianasalnikova08@yandex.ru (D.I.S.); f.bogdanov.f@yandex.ru (F.B.B.); alex.scherbakov@gmail.com (A.M.S.); 3 A. N. Nesmeyanov Institute of Organoelement Compounds, Russian Academy of Sciences, Vavilov Str. 28, 119334 Moscow, Russia; alex@ineos.ac.ru; 4 Molecular Genetics Laboratory, Institute of Clinical Medicine, National Research Lobachevsky State University of Nizhny Novgorod, Prospekt Gagarina 23, 603950 Nizhny Novgorod, Russia

**Keywords:** chalcone, sulfonamide, breast cancer, antiproliferative activity, 4-hydroxytamoxifen resistance, estrogen receptor α

## Abstract

Breast and other estrogen receptor α-positive cancers tend to develop resistance to existing drugs. Chalcone derivatives possess anticancer activity based on their ability to form covalent bonds with targets acting as Michael acceptors. This study aimed to evaluate the anticancer properties of a series of chalcones (**7a**–**l**) with a sulfonamide group attached to the vinyl ketone moiety. Chalconesulfonamides showed a potent antiproliferative effect at low micromolar concentrations against several cancer cell lines, including ERα-positive 4-hydroxytamoxifen-resistant MCF7/HT2. Immunoblotting of samples treated with the lead compound **7e** revealed its potent antiestrogenic activity (ERα/GREB1 axis) and induction of PARP cleavage (an apoptosis marker) in breast cancer cells. The obtained compounds represent a promising basis for further development of targeted drugs blocking hormone pathways in cancer cells.

## 1. Introduction

**Figure 1:**
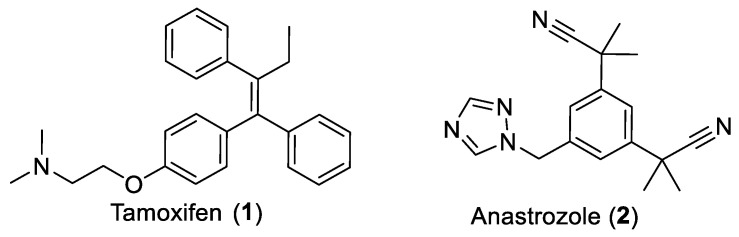
Structures of tamoxifen (**1**) and anastrozole (**2**).

Despite the progress in antiestrogen therapy, achieved through the development of selective estrogen receptor modulators (SERMs, for example, tamoxifen (**1**)) and aromatase inhibitors (AIs, for example, anastrozole (**2**), Figure 1), the development of tumor resistance reduces the efficiency of anticancer treatment and decreases survival rates of patients with breast, ovarian, and prostate cancers [1,2,3,4,5]. Therefore, the development of new chemotherapeutic agents with improved efficacy and that are capable of circumventing resistance mechanisms is an important task in pharmacology.

**Figure 2:**
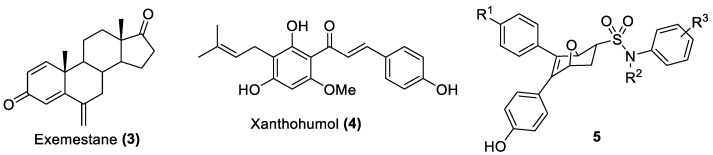
Exemestane (**3**)—AI with a covalent mechanism of action; chalcone **4** with antiproliferative activity; and general structure of sulfonamides **5** with antiestrogenic activity.

Development of covalent inhibitors is one of the promising directions in drug discovery and therapeutics [6]. Compared to common drugs with noncovalent interactions, covalent inhibitors could have increased efficacy, selectivity, and duration of action of the drug on the target [7]. Exemestane (**3**, Figure 2) is a covalent AI that is widely used in the treatment of estrogen receptor α (ER α)-positive breast cancer [8]. Unlike other AIs, which are reversible and competitive substrates, exemestane forms a covalent bond with the enzyme, leading to a prolonged and irreversible inhibition of aromatase activity [9]. Exemestane has shown significant benefits in terms of disease-free survival and overall survival rates in several clinical trials for the treatment of breast cancer [10,11]. The high antitumor efficacy of exemestane highlights the potential for the development of covalent inhibitors in the treatment of hormone-receptor-positive cancers [12].

Chalcones are naturally occurring compounds that have drawn the attention of researchers regarding the development of new anticancer agents [13,14]. Chalcone derivatives, such as xanthohumol (**4**), have been shown to possess potent anticancer activity by acting as Michael acceptors and covalently inhibiting various proteins, including IκB kinase [15] and thioredoxin reductase [16]. Recently, it was shown that the antiestrogenic effects of chalcones may underlie their anticancer activity [17,18]. Thus, the evaluation of new chalcone-based compounds may contribute to the development of new anticancer antiestrogen agents.

Sulfonamides and their derivatives represent another important class of compounds with antiestrogenic activity [19,20,21]. For example, sulfonamides on an oxabicyclo [2.2.1] heptene scaffold (**5**, Figure 2) are potent antiestrogens with nanomolar antiproliferative potency against breast cancer cells. In addition, other sulfonamides are known inhibitors of carbonic anhydrase IX, phosphatidylinositol-3-kinase (PI3K), and protein kinase BRAF with validated antitumor properties [22,23,24,25].

**Figure 3:**
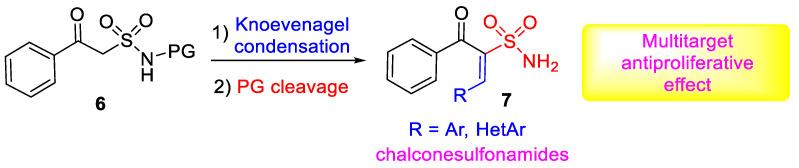
Development of chalconesulfonamide **7** with potential anticancer activity.

Taking into account the prospects for the development of chalcones as a privileged scaffold with a covalent mechanism with anticancer action and the antiestrogenic activity of sulfonamides, our research aims to design and evaluate the anticancer properties of novel chalconesulfonamides. According to a survey of the literature, the synthesis and biological properties of chalconesulfonamides have not been studied yet. Thereby, using Knoevenagel condensation of 2-oxo-2-phenylethanesulfonamide (**6**), we synthesized a series of chalconesulfonamides **7** and estimated their antiproliferative and proapoptotic activity and action on signaling pathways (Figure 3).

## 2. Results

### 2.1. Chemistry

The generation of nucleophilic reagents by ionization of C-H and N-H groups is a common method in organic synthesis. Metalation of methanesulfonamide derivatives allows for the synthesis of aliphatic sulfonamides with complex structures that are difficult to obtain with other methods [26,27,28]. Therefore, we use the metalation of methanesulfonamide as a useful approach to obtain the key intermediate **6** for the synthesis of the target compound **7**.

**Scheme 1:**
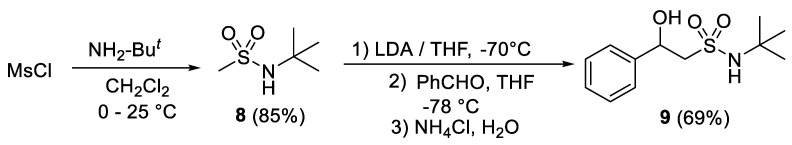
The synthesis of 2-hydroxy-2-phenylethane-1-sulfonamide **9**.

To obtain methanesulfonamide **8** for the subsequent C-H activation, methanesulfonyl chloride was reacted with *tert*-butylamine. (Scheme 1). In the second step, sulfonamide **8** was metalated with LDA and reacted with benzaldehyde to give 2-hydroxyethanesulfonamide **9** in good yield [26]. 

**Scheme 2:**
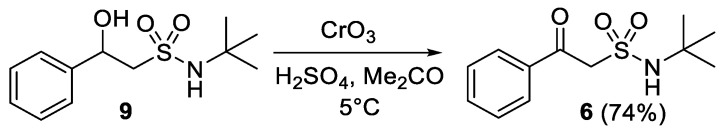
The synthesis of *N*-(*tert*-butyl)-2-oxo-2-phenylethane-1-sulfonamide (**6**).

In the next step, ketone **6** was synthesized in good yield via Jones oxidation of β-hydroxysulfonamide **9**, using a chromium oxide (VI) solution in the presence of dilute sulfuric acid in acetone (Scheme 2) [29]. Cr (VI) reagents are often the preferred method due to their availability and scalability [30,31]. As we required gram quantities of ketone **6** for this and future research, we chose not to use the more expensive, less stable, and less available oxidizing reagents such as Swern oxidation reagent or Dess–Martin periodinane.

**Scheme 3:**
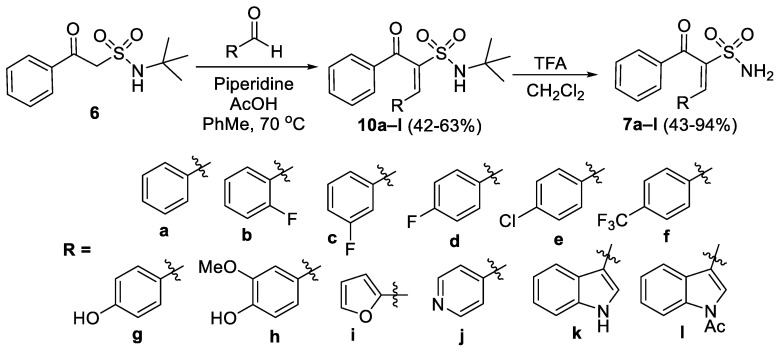
The synthesis of a series of chalconesulfonamides **7a**–**l**.

To obtain the target chalconesulfonamide **7**, the Knoevenagel condensation of 2-oxo-2-phenylethanesulfonamide **6** with arene or heteroarene carbaldehydes was studied. The use of classical conditions [32] with catalytic amounts of piperidine acetate did not lead to the formation of the adduct **10**. It was observed that the reaction with benzoylmethanesulfonamide **6** required additional equivalents of piperidine acetate, apparently due to the formation of dianion. Therefore, condensation of the activated methylene group of sulfonamide **6** with arene or heteroarene carbaldehydes in toluene at 70 °C afforded *N*-*tert*-butyl-derivatives of chalcone-2-sulfonamides **10a**–**l** in moderate to good yields (Scheme 3). It is noteworthy that the reaction proceeds with good stereoselectivity, leading to the formation of one of the *E*/*Z* isomers.

In the final step, cleavage of the *N*-*tert*-butyl group of the adducts **10a**–**l** via treatment with TFA in dichloromethane afforded the target series of chalcone-2-sulfonamides **7a**–**j**,**l** in high yields (88–94%). It is worth mentioning that when the *tert*-butyl protecting group is removed from the 1-*H*-indole derivative **10k**, there are side reactions involving dimerisation and polymerisation that result in a lower yield (43%) of the desired sulfonamide **7k**. Therefore, it may be worth considering obtaining compound **7k** via alkaline hydrolysis of the acetyl derivative **7l**. The structures of all derivatives **7a**–**l** were confirmed with ^1^H and ^13^C NMR spectra and HRMS data. The purity of compounds **7a**–**l** (>95%) was confirmed with HPLC. Analysis of the NMR spectra confirmed the structure of the proposed chalconesulfonamides **10** and **7**: the starting 2-oxo-2-phenylethanesulfonamide **6** contains a singlet signal of a CH_2_-group with a chemical shift value of 4.66 ppm, which is absent in the spectrum of compound **10a**. Instead, a singlet signal of a CH-group occurs in the 7.91–7.88 region, which corresponds to the signal of the arylmethylidene fragment. Finally, in the spectra of compound **7a**, the signals corresponding to *tert*-butyl and NH-groups are absent, but there is a singlet signal of the NH_2_ sulfonamide moiety with a chemical shift value close to 5.12 ppm.

**Figure 4:**
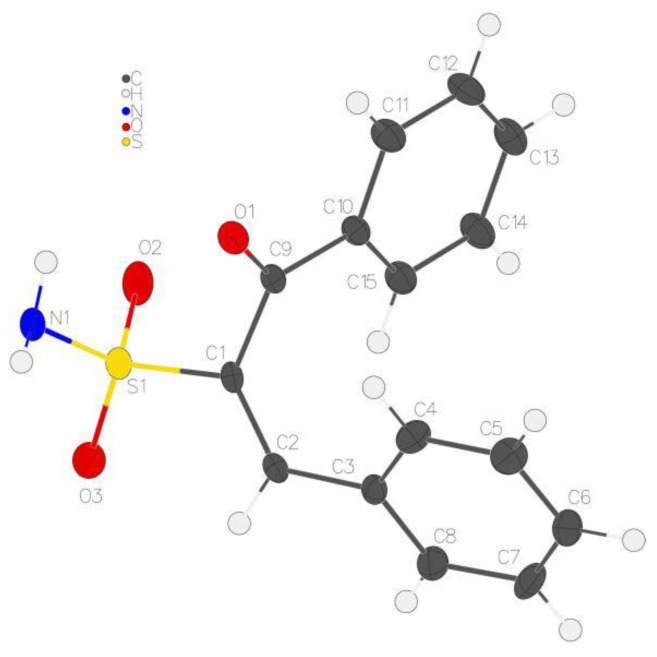
General view of molecule **7a**.

The ^1^H NMR and ^13^C spectra do not allow for the *E*/*Z* configuration of the synthesized isomer of compound **7a** to be determined. Therefore, to establish the stereochemistry of compound **7a**, we used X-ray analysis that showed that the obtained 3-oxo-1,3-diphenylprop-1-en-2-sulfonamide (**7a**) is the *E*-isomer (Figure 4). The present X-ray crystallography (experimental details are presented in ESI) result is in agreement with previously published data on the structure of related compounds [33]. The structure of **7a** crystallized in noncentrosymmetric space group *Cc* and contains four molecules in a unique part of the unit cell. The disposition of substituents at the double C1–C2 bond (mean length is 1.341 Å) that corresponds to E-configuration is caused due to two possible reasons. The first is bulky phenyl and sulfonamide groups and the second one is hydrogen bonding of sulfonamide groups in crystal. Indeed, the molecules of **7a** form two types of centrosymmetric H-bonded dimers. In turn, H-bonded dimers are assembled into infinite chains parallel to the b-side of the unit cell. The experimental details and figures of the dimers and crystal packing can be found in the Supplementary Materials (Sections: X-ray study of **7a** and Hydrogen bonding of **7a**, Tables S1 and S2, Figures S1–S3).

**Scheme 4:**
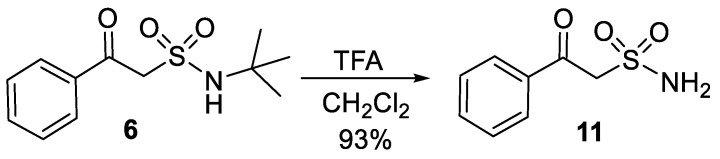
The synthesis of 2-oxo-2-phenylethane-1-sulfonamide (**11**).

Furthermore, in order to determine the role of the (hetero)arylmethylidene moiety in the antiproliferative activity of sulfonamide **7**, we synthesized analog **11** without the chalcone moiety (Scheme 4).

### 2.2. Antiproliferative Effects and Selectivity

Screening of the antiproliferative properties of the synthesized 2-oxo-2-phenylethane sulfonamide **11** and chalconesulfonamides **7a**–**l** was performed with an MTT test on breast cancer cells MCF7, hormone-resistant MCF7/HT2 subline [34], leukemia cell line K-562, resistant subline K-562/4 with Pgp [35,36], and colon cancer cells HCT-116. The results regarding antiproliferative activity are summarized in Table 1.

The results in Table 1 demonstrate that the synthesized chalconesulfonamides **7a**–**l** suppress the growth of tumor cells at low micromolar concentrations. SAR analysis among compounds **7a** and **11** clearly highlights the significance of the chalcone moiety for the antiproliferative potency of these derivatives. A comparison of the unsubstituted chalconesulfonamide **7a** with monofluorophenyl derivatives **7b**,**c**,**d** revealed that the introduction and position of the fluorine atom have no impact on the antiproliferative activity. However, the introduction of a chlorine atom (**7e**) or trifluoromethyl group (**7f**) at the *para*-position of the arylidene moiety enhances the antiproliferative activity against all tumor cells, especially against the resistant K-562/4 line that expresses P-glycoprotein. The introduction of hydroxy and methoxy (**7g**,**h**) groups results in reduced activity against all cancer cells. This suggests a correlation between antiproliferative activity, LogP, and the donor–acceptor characteristics of the substituents. 

The IC_50_ values for furan **7i**, pyridine **7j,** and the indole **7k**,**l** derivatives indicate that the inclusion of heterocycles in the chalcone structure results in a decrease in activity. Of note, among the indole derivatives, the *N*-acyl derivative **7l** exhibits higher activity than the unsubstituted **7k**. This reinforces the suggestion that LogP has a significant impact on the antiproliferative activity of synthesized sulfonamides **7a**–**l**. Overall, chalconesulfonamides **7a**–**l** display low micromolar inhibitory concentrations against leukemia, colon, and breast cancer cells. Furthermore, they retain a potent antiproliferative effect on hormone-resistant (MCF7/HT2) and Pgp-positive (K-562/4) sublines.

Evaluation of cytotoxicity and selectivity is an important step in drug development. Normal cell lines are used for such experiments—this avoids the need for animal experiments in the early stages of development. Human fibroblasts are chosen as a model of normal cells as previously described [37,38]. MCF7 breast cancer cells and fibroblasts were treated with compounds **7e** and **11** for 72 h and the cell viability was evaluated with the resazurin test [39,40,41].

The IC_50_ values obtained in this assay were consistent with the data that were obtained with the MTT test (Table 1). Thus, compound **7e,** at a concentration of about 2.5 μM, caused 50% of tumor cells to die, while fibroblasts were less sensitive to its effects. The selectivity data are presented in Table 2.

### 2.3. Signaling Pathways Regulated by Chalconesulfonamide ***7e***

**Figure 5:**
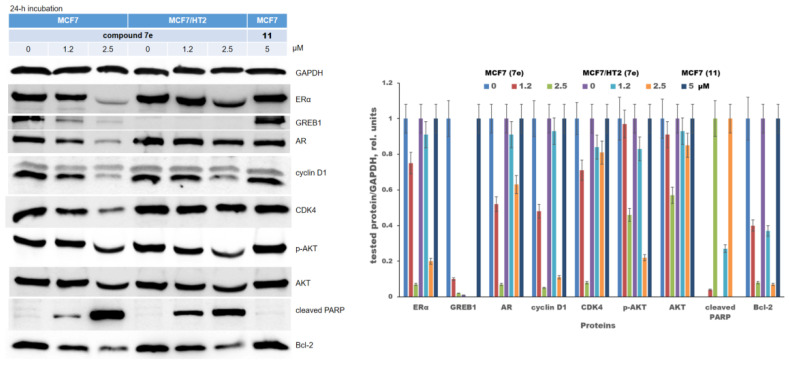
Impact of lead compound **7e** on signaling pathways in breast cancer cells. Protein expression was detected with immunoblotting after a 24 h treatment. Compound **11** was applied as a negative control. GAPDH (a loading control)—glyceraldehyde-3-phosphate dehydrogenase; ERα—estrogen receptor α; GREB1—gene regulated in breast cancer 1 protein; AR—androgen receptor, CDK4—cyclin-dependent kinase 4; AKT—RAC-α serine/threonine-protein kinase; PARP—Poly (ADP-ribose) polymerase 1; and Bcl-2—B-cell CLL/lymphoma 2. Densitometry for the tested proteins/GAPDH ratio was carried out using ImageJ software (version 1.53m).

The chalconesulfonamide **7e**, which showed activity against hormone-sensitive and hormone-resistant cells at a micromolar range of concentration, was selected for in-depth biological studies. MCF7 and MCF7/HT2 breast cancer cells were treated with compound **7e** for 24 h, and then samples were prepared for immunoblotting. The selected breast cancer cell lines differ primarily in the activity of the steroid hormone receptor signaling pathways. Prolonged incubation with the antiestrogen HT resulted in partial inhibition of ERα activity [34], limiting further application of such treatment. The compound **7e** caused a decrease in ERα expression in MCF7 cells; the selected compound at a concentration of 2.5 μM almost completely blocked ERα expression (Figure 5). The effect of compound **7e** on ERα expression in MCF7/HT2 cells was also observed, but it was less significant. Sulfonamides are a promising class for the development of selective antiestrogens. While the more polar secondary sulfonamides were weak ligands, some of the tertiary sulfonamides had very good estrogen receptor α binding affinity [19,20].

ERα activity in cancer cells can be assessed by analyzing the expression of genes that are under the control of estrogens [42]. Such genes include GREB1; its expression is regulated by estrogens and antiestrogens (SERMs or SERDs). A dose-dependent decrease in GREB1 expression was found in MCF7 cells that express this protein, suggesting an antiestrogenic effect of compound **7e**. Compound **11,** selected as a negative control in the experiments, had no effect on ERα and GREB1 expression. Hormone dependence of MCF7 cells is supported mainly through the ERα signaling pathway [43], and AR expression is also detected in these cells—this steroid hormone receptor is considered as a new target in breast cancer cells [44,45]. Chalconesulfonamide **7e** induced a dose-dependent decrease in AR expression in MCF7 cells. Interestingly, AR expression was not altered in hormone-resistant cells. This may indicate the resistance mechanisms that are realized in MCF7/HT2 cells. In subsequent experiments, proteins that regulate the cell cycle and support cell survival were analyzed. Cyclin D1 is among the key regulators of the cell cycle and its expression increases significantly when cells are exposed to growth factors [46,47]. A number of epidemiologic studies suggest that cyclin D1 expression is increased in 50% of breast cancers [48]. In both parental and hormone-resistant cells, chalconesulfonamide **7e** decreased the expression of cyclin D1, while compound **11** did not alter its expression. MCF7 cells treated with chalconesulfonamide **7e** revealed decreased expression of CDK4. No changes in CDK4 expression were detected in MCF7/HT2 treated with **7e**. This may indicate mechanisms of partial resistance of MCF7/HT2 cells to **7e**, as well as ways to search for new drug combinations.

The PI3K/AKT axis is one of the most frequently activated intracellular pathways, which is commonly involved as a balancer in cancer [49]. AKT kinase is activated when the cell is exposed to various growth stimuli [50]. This kinase plays a role in increasing the survival of tumor cells and stimulating their proliferation. A decrease in phosphorylated AKT was found in parental and hormone-resistant cells treated with **7e**. Compound **11** had no effect on AKT activity. Two markers of cell death, PARP and Bcl-2, were evaluated in subsequent experiments. Induction of programmed cell death causes activation of a number of enzymes, the final step in such signaling is the cleavage of PARP, and accumulation of cleaved PARP is indicative of apoptotic cell death. Sulfonamide **11** did not induce accumulation of cleaved PARP. In MCF7 and MCF7/HT2 cells treated with **7e**, a significant amount of cleaved PARP was detected. The accumulation of cleaved PARP was accompanied by a decrease in the expression of the antiapoptotic protein Bcl-2. As in the case of PARP, compound **11,** selected as a negative control, did not affect the expression of the marker. All described observations suggest a complex effect of chalconesulfonamide **7e** on the signaling pathways of breast cancer cells. The selected sulfonamide regulates steroid receptor signaling, expression of cell cycle regulators, and apoptosis proteins. The detected effects are largely realized in MCF7 hormone-dependent breast cancer cells; hormone-resistant cells are also sensitive to chalconesulfonamide **7e**, but some of the investigated proteins are not regulated by the selected compound. These data may provide a good basis for searching synergistic combinations of lead compound **7e** with other antitumor drugs for the development of new therapeutic approaches for hormone-resistant breast cancer.

## 3. Discussion

To date, C-H activation of the methylene group of 2-oxo-2-phenylethanesulfonamide (**6**), an example of β-ketosulfonamides, is presented by a reaction with isatoic anhydride and electrophilic fluorination [51,52]. In this study, we explored the possibility of using compound **6** as a methylene component in the Knoevenagel reaction to obtain chalconesulfonamide **7**. Thus, the reaction of benzoylmethanesulfonamide **6** with aldehydes afforded previously unknown Michael acceptors containing a sulfonamide group. Using various aromatic and heteroaromatic aldehydes, twelve examples of *E*-chalconesulfonamides **7a**–**l** were synthesized.

Screening of the antiproliferative activity showed that the chalcone scaffold was responsible for the inhibition of solid and hematological cancer cells at low micromolar concentrations. According to the SAR results, the antiproliferative activity of chalconesulfonamides **7a**–**l** increases with higher lipophilicity of the arylmethyledene fragment. The most active derivatives, specifically *para*-chloro- and *para*-trifluoromethylphenyl derivatives (**7e**,**f**), have shown IC_50_ values ranging from 1.3 to 5.1 µM against breast, colon, and leukemia cancer cells. When tested against resistant breast and leukemia cells, these compounds exhibited IC_50_ values between 2.5 and 3.8 µM. Selected for in-depth biological studies, **7e** reduces the expression of estrogen and androgen receptors, key factors in the development and resistance of luminal breast cancer. The observed proapoptotic activity and effects on cell cycle regulators may provide an explanation for the antiproliferative activity of chalconesulfonamides against colon cancer and leukemia cells. The evaluation of the cytotoxicity of the lead compound **7e** on fibroblasts demonstrated almost a 2-fold selectivity against MCF7 cancer cells, providing a basis for further research on chalconesulfonamides as anticancer agents.

The comparison of the biological properties of synthesized chalconesulfonamides **7a**–**l** with other chalcone derivatives indicates that chalconesulfonamides exhibit similar or greater antiproliferative activity [53,54]. Previously, our colleagues synthesized chalcone analogues based on steroidal benzylidenes. These compounds and chalconesulfonamides demonstrated similar IC_50_ values against MCF7. Studying the mechanism of action revealed that steroidal benzylidenes are able to cause both ERα inhibition and down regulation [55]. However, the discovered chalconesulfonamide **7e** required a 7-fold lower concentration in order to decrease the expression of ERα. This points out that the mechanism of action of chalconesulfonamide **7e** against hormone-positive cancer cells is based on an antiestrogenic and proapoptotic effect.

## 4. Materials and Methods 

### 4.1. Synthesis 

NMR spectra were recorded on a Varian VXR-400 instrument (Varian Inc, Palo Alto, CA, USA) operated at 400 MHz (^1^H NMR) and 100 MHz (^13^C NMR). Chemical shifts were measured in DMSO-*d*_6_ and CDCl_3_ using tetramethylsilane as an internal standard. Analytical TLC was performed on Silica Gel F254 plates (Merck, Boston, MA, USA), using column chromatography with a SilicaGel Merck 60. Melting points were determined using a Buchi SMP-20 apparatus (Flawil, Switzerland) and were uncorrected. High-resolution mass spectra were recorded with electron spray ionization on a Bruker Daltonics microOTOF-QII instrument (Billerica, MA, USA). HPLC was performed using a Shimadzu Class-VP V6.12SP1 (Kyoto, Japan) system with a UV-vis detector: column Kromasil 100-5-C18 4.6 × 250 mm and flow—1 mL/min; samples were dissolved in MeCN. All solvents, chemicals, and reagents were obtained commercially and used without purification. The purity of final compounds **7a**–**m** was ≥95% as determined with HPLC analysis.

The *N*-(*tert*-butyl)methanesulfonamide (**8**) and *N*-(*tert*-butyl)-2-hydroxy-2-phenylethane-1-sulfonamide (**9**) were synthesized as described in [26].

*N*-(*tert*-Butyl)-2-oxo-2-phenylethane-1-sulfonamide (**6**)

To a stirring solution of *N*-(*tert*-butyl)-2-hydroxy-2-phenylethane-1-sulfonamide (**9** [26], 5.6 g, and 22.0 mmol) in acetone (30.0 mL), a solution of CrO_3_ (3.0 g and 30.0 mmol) and concentrated H_2_SO_4_ (3.0 mL) in water (20 mL) at 0–5 °C under argon was added. The cooling bath was removed, and the resulting mixture was stirred at room temperature for 4 h. Upon completion, the reaction mixture was diluted with cold water (80 mL) and extracted with ethyl acetate (3 × 20 mL); the organic fractions were combined, washed with 5% NaHCO_3,_ water, dried over anhydrous sodium sulfate, filtered, and concentrated in vacuo. The residue was purified with flash column chromatography (silica gel, hexane—EtOAc 1:1). The yield was 4.7 g (85%) of colorless crystals. ^1^H NMR (400 MHz, CDCl_3_): δ 7.98 (dd, *J*^1^ = 7.8, *J*^2^ =1.7 Hz, 2H, Ar), 7.63 (t, *J* = 7.4 Hz, 1H, Ar), 7.50 (t, *J* = 7.4 Hz, 2H, Ar), 4.84 (s, 1H, NH), 4.66 (s, 2H, CH_2_), and 1.41 (s, 9H, 3CH_3_). ^13^C NMR (100 MHz, CDCl_3_): δ 190.3, 135.7, 134.5, 128.9 (2C), 128.8 (2C), 61.1, 55.2, and 30.1 (3C). HRMS (ESI) *m*/*z* [M + Na]^+^: calculated for C_12_H_17_NNaO_3_S 278.0827, found 278.0864.

(*E*)-*N*-(*tert*-Butyl)-3-oxo-1,3-diphenylprop-1-ene-2-sulfonamide (**10a**)

To a stirring solution of *N*-(*tert*-butyl)-2-oxo-2-phenylethan-1-sulfonamide (**6**, 150 mg, and 0.60 mmol) in toluene (5.0 mL), piperidine (145.0 μL and 1.50 mmol), acetic acid (70.0 μL and 1.20 mmol), and benzaldehyde (76.0 μL and 1.0 mmol) were added at room temperature and heated to 70 °C for 6 h. Upon completion of the reaction, the mixture was cooled, diluted with ethyl acetate (50 mL), washed with 5% aqueous HCl (15 mL) and water (15 mL), dried over sodium sulfate, filtered, and concentrated in vacuo. The residue was purified with column chromatography (hexane—EtOAc (1:1)). The yield was 93 mg (46%) of colorless powder. ^1^H NMR (400 MHz, CDCl_3_): δ 7.91–7.88 (m, 3H, 2Ar, CH), 7.46 (t, *J* = 7.4 Hz, 1H, Ar), 7.31 (t, *J* = 7.4 Hz, 1H, Ar), 7.27-7.17 (m, 5H, Ar), 4.65 (s, 1H, NH), and 1.44 (s, 9H, 3CH_3_). ^13^C NMR (100 MHz, CDCl_3_): δ 193.6, 139.7, 138.8, 135.3, 134.3, 132.0, 130.4, 129.8 (2C), 129.8 (2C), 128.8 (2C), 128.7 (2C), 55.4, and 29.9 (3C). HRMS (ESI) *m*/*z* [M + H]^+^: calculated for C_19_H_22_NO_3_S 344.1315, found 344.1333.

(*E*)-*N*-(*tert*-Butyl)-1-(2-fluorophenyl)-3-oxo-3-phenylprop-1-ene-2-sulfonamide (**10b**)

This compound was prepared from **6** and 2-fluorobenzaldehyde as described for **10a**. The yield was 119 mg (55%) of colorless powder. ^1^H NMR (400 MHz, CDCl_3_): δ 8.06 (s, 1H, CH), 7.85 (d, *J* = 7.8 Hz, 2H, Ar), 7.45 (t, *J* = 7.4 Hz, 1H, Ar), 7.31 (t, *J* = 7.4 Hz, 2H, Ar), 7.24–7.17 (m, 1H, Ar), 7.09 (t, *J* = 7.4 Hz, 1H, Ar), 6.95 (t, *J* = 7.4 Hz, 1H, Ar), 6.88 (t, *J* = 7.4 Hz, 1H, Ar), 4.72 (s, 1H, NH), and 1.44 (s, 9H, 3CH_3_). ^13^C NMR (100 MHz, CDCl_3_): δ 193.1, 160.5, (d, *J* = 253.4 Hz, C-F), 141.5, 135.3, 134.3, 132.4 (d, *J* = 8.7 Hz), 131.9 (d, *J* = 2.7 Hz), 130.1, 129.7 (2C), 128.6 (2C), 124.3 (d, *J* = 2.7 Hz), 120.4 (d, *J* = 12.6 Hz, C-F), 115.9 (d, *J* = 21.7 Hz, C-F), 55.5, and 29.9 (3C). HRMS (ESI) (*m*/*z*) [M + H]^+^: calculated for C_19_H_21_FNO_3_S 362.1221, found 362.1238.

(*E*)-*N*-(*tert*-Butyl)-1-(3-fluorophenyl)-3-oxo-3-phenylprop-1-ene-2-sulfonamide (**10c**)

This compound was prepared from **6** and 3-fluorobenzaldehyde as described for **10a**. The yield was 126 mg (58%) of colorless powder. ^1^H NMR (400 MHz, CDCl_3_): δ 7.89 (d, *J* = 7.4 Hz, 2H, Ar), 7.82 (s, 1H, CH), 7.49 (t, *J* = 7.0 Hz, 1H, Ar), 7.34 (t, *J* = 7.8 Hz, 2H, Ar), 7.20–7.12 (m, 1H, Ar), 7.01 (d, *J* = 7.8 Hz, 1H, Ar), 6.94–6.89 (m, 2H, Ar), 4.64 (s, 1H, NH), and 1.43 (s, 9H, 3CH_3_). ^13^C NMR (100 MHz, CDCl_3_): δ 193.2, 162.5, (d, *J* = 248.2 Hz, C-F), 141.2, 137.1, 135.2, 134.5, 134.1 (d, *J* = 8.4 Hz), 130.4 (d, *J* = 8.4 Hz), 129.8 (2C), 128.8 (2C), 125.4 (d, *J* = 3.3 Hz), 117.4 (d, *J* = 21.6 Hz), 116.2 (d, *J* = 21.6 Hz), 55.5, and 29.9 (3C). HRMS (ESI) (*m*/*z*) [M + H]^+^: calculated for C_19_H_21_FNO_3_S 362.1221, found 362.1264.

(*E*)-*N*-(*tert*-Butyl)-1-(4-fluorophenyl)-3-oxo-3-phenylprop-1-ene-2-sulfonamide (**10d**)

This compound was prepared from **6** and 4-fluorobenzaldehyde as described for **10a**. The yield was 132 mg (61%) of colorless powder. ^1^H NMR (400 MHz, CDCl_3_): δ 7.89 (d, *J* = 7.4 Hz, 2H, Ar), 7.84 (s, 1H, CH), 7.49 (t, *J* = 7.4 Hz, 1H, Ar), 7.33 (t, *J* = 7.4 Hz, 2H, Ar), 7.21 (t, *J* = 8.2 Hz, 2H, Ar), 6.88 (d, *J* = 8.2 Hz, 2H, Ar), 4.63 (s, 1H, NH), and 1.43 (s, 9H, 3CH_3_). ^13^C NMR (100 MHz, CDCl_3_): δ 193.5, 163.7 (d, *J* = 253.8 Hz, C-F), 139.6, 137.4, 135.2, 134.5, 131.8 (d, *J* = 9.1 Hz, 2C), 129.8 (2C) 128.8 (2C), 128.2 (d, *J* = 3.2 Hz), 116.1 (d, *J* = 22.7 Hz, 2C), 55.4, and 29.9 (3C). HRMS (ESI) (*m*/*z*) [M − H]^−^: calculated for C_19_H_19_FNO_3_S 360.1075, found 360.0943.

(*E*)-*N*-(*tert*-Butyl)-1-(4-chlorophenyl)-3-oxo-3-phenylprop-1-ene-2-sulfonamide (**10e**)

This compound was prepared from **6** and 4-chlorobenzaldehyde as described for **10a**. The yield was 113 mg (51%) of colorless powder. ^1^H NMR (400 MHz, CDCl_3_): δ 7.89 (d, *J* = 7.8 Hz, 2H, Ar), 7.81 (s, 1H, CH), 7.50 (m, 1H, Ar), 7.34 (t, *J* = 7.8 Hz, 2H, Ar), 7.19–7.14 (m, 4H, Ar), 4.63 (s, 1H, NH), and 1.42 (s, 9H, 3CH_3_). ^13^C NMR (100 MHz, CDCl_3_): δ 193.3, 140.3, 137.1, 136.6, 135.1, 134.6, 130.9 (2C), 130.5, 129.8 (2C), 129.1(2C), 128.8 (2C), 55.5, and 29.9 (3C). HRMS (ESI) *m*/*z* [M + H]^+^: calculated for C_19_H_21_ClNO_3_S 378.0925, found 378.0897.

(*E*)-*N*-(*tert*-Butyl)-3-oxo-3-phenyl-1-(4-(trifluoromethyl)phenyl)prop-1-ene-2-sulfonamide (**10f**)

This compound was prepared from **6** and 4-(trifluoromethyl)benzaldehyde as described for **10a**. The yield was 152 mg (63%) of colorless powder. ^1^H NMR (400 MHz, CDCl_3_): δ 7.90 (s, 1H, CH), 7.88 (d, *J* = 8.0 Hz, 2H, Ar), 7.50 (t, *J* = 7.8 Hz, 1H, Ar), 7.45 (d, *J* = 8.2 Hz, 2H, Ar), 7.37–7.31 (m, 4H, Ar), 4.65 (s, 1H, NH), and 1.43 (s, 9H, 3CH_3_). ^13^C NMR (100 MHz, CDCl_3_): δ 193.0, 142.3, 136.5, 135.5, 135.0, 134.7, 131.8 (q, *J* = 32.4 Hz, C-CF_3_), 129.9 (2C), 129.8 (2C), 128.9 (2C), 125.7 (2C), 123.4 (q, *J* = 278.1 Hz, CF_3_), 55.6, and 29.9 (3C). HRMS (ESI) *m*/*z* [M + H]^+^: calculated for C_20_H_21_F_3_NO_3_S 412.1189, found 412.1186.

(*E*)-*N*-(*tert*-Butyl)-1-(4-hydroxyphenyl)-3-oxo-3-phenylprop-1-ene-2-sulfonamide (**10g**)

This compound was prepared from **6** and 4-hydroxybenzaldehyde as described for **10a**. The yield was 104 mg (48%) of colorless powder. ^1^H NMR (400 MHz, CDCl_3_): δ 7.88 (d, *J* = 7.4 Hz, 2H, Ar), 7.80 (s, 1H, CH), 7.47 (t, *J* = 7.4 Hz, 1H, Ar), 7.31 (t, *J* = 7.4 Hz, 2H, Ar), 7.08 (d, *J* = 8.2 Hz, 2H, Ar), 6.61 (d, *J* = 8.2 Hz, 2H, Ar), 5.80 (s, 1H, OH), 4.62 (s, 1H, NH), and 1.40 (s, 9H, 3CH_3_). ^13^C NMR (100 MHz, CDCl_3_): δ 194.0, 158.0, 139.0, 136.6, 135.4, 134.3, 132.1 (2C), 129.9 (2C), 128.7 (2C), 124.3, 115.9 (2C), 55.4, and 29.9 (3C). HRMS (ESI) (*m*/*z*) [M + H]^+^: calculated for C_19_H_22_NO_4_S 360.1264, found 360.1284.

(*E*)-*N*-(*tert*-Butyl)-1-(4-hydroxy-3-methoxyphenyl)-3-oxo-3-phenylprop-1-ene-2-sulfonamide (**10h**)

This compound was prepared from **6** and 4-hydroxy-3-methoxybenzaldehyde as described for **10a.** The yield was 98 mg (42%) of colorless powder. ^1^H NMR (400 MHz, CDCl_3_): δ 7.95 (d, *J* = 7.4 Hz, 2H, Ar), 7.78 (s, 1H, CH), 7.50 (t, *J* = 7.4 Hz, 1H, Ar), 7.35 (t, *J* = 7.4 Hz, 2H, Ar), 6.85 (dd, *J*^1^ = 8.2, *J*^2^ =1.6 Hz, 2H, Ar), 6.76 (d, *J* = 8.2 Hz, 2H, Ar), 6.63 (s, 1H, Ar), 5.82 (s, 1H, OH), 4.62 (s, 1H, NH), 3.58 (s, 3H, CH_3_), and 1.42 (s, 9H, 3CH_3_). ^13^C NMR (100 MHz, CDCl_3_): δ 194.0, 148.1, 146.3, 139.0, 136.7, 135.6, 134.4, 129.8 (2C), 128.9 (2C), 125.4, 124.1, 114.7, 111.7, 55.7, 55.2, and 29.9 (3C). HRMS (ESI) (*m*/*z*) [M – H]^−^: calculated for C_20_H_24_NO_5_S 388.1224, found 388.1022.

(*E*)*-N-*(*tert*-Butyl)-1-(furan-2-yl)-3-oxo-3-phenylprop-1-ene-2-sulfonamide (**10i**).

This compound was prepared from **6** and furan-2-carbaldehyde as described for **10a**. The yield was 121 mg (62%) of colorless powder. ^1^H NMR (400 MHz, CDCl_3_): δ 7.97 (d, *J* = 7.0 Hz, 2H, Ar), 7.57–7.51 (m, 2H, CH, Ar), 7.41 (t, *J* = 7.4 Hz, 2H, Ar), 7.17 (s, 1H, Ar), 6.60 (d, *J* = 4.2 Hz, 1H, Ar), 6.35–6.31 (m, 1H, Ar), 4.56 (s, 1H, NH), and 1.40 (s, 9H, 3CH_3_). ^13^C NMR (100 MHz, CDCl_3_): δ 192.5, 147.7, 146.2, 136.4, 135.9, 134.0, 129.6 (2C), 128.6 (2C), 124.7, 117.7, 112.4, 55.4, and 29.9 (3C). HRMS (ESI) *m*/*z* [M − H]^−^: calculated for C_17_H_18_NO_4_S 332.0962, found 332.0811.

(*E*)*-N-*(*tert*-Butyl)-3-oxo-3-phenyl-1-(pyridin-4-yl)prop-1-ene-2-sulfonamide (**10j**)

This compound was prepared from **6** and pyridine-4-carbaldehyde as described for **10a**. The yield was 109 mg (54%) of colorless powder. ^1^H NMR (400 MHz, CDCl_3_): δ 8.45 (d, *J* = 5.5 Hz, 2H, Ar), 7.86 (d, *J* = 7.8 Hz, 2H, Ar), 7.75 (s, 1H, CH), 7.50 (t, *J* = 7.4 Hz, 1H, Ar), 7.34 (t, *J* = 7.8 Hz, 2H, Ar), 7.07 (d, *J* = 5.5 Hz, 2H, Ar), 4.82 (s, 1H, NH), and 1.43 (s, 9H, 3CH_3_). ^13^C NMR (100 MHz, CDCl_3_): δ 192.5, 150.3 (2C), 144.5, 139.5, 135.0, 134.9, 134.8, 129.8 (2C), 128.9 (2C), 123.0 (2C), 55.7, and 29.9 (3C). HRMS (ESI) *m*/*z* [M + H]^+^: calculated for C_18_H_21_N_2_O_3_S 345.1267, found 345.1294.

(*E*)-*N*-(*tert*-Butyl)-1-(1*H*-indol-3-yl)-3-oxo-3-phenylprop-1-ene-2-sulfonamide (**10k**)

This compound was prepared from **6** and 1*H*-indole-3-carbaldehyde as described for **10a**. The yield was 99 mg (43%) of yellowish powder. ^1^H NMR (400 MHz, CDCl_3_): δ 8.77 (br s, 1H, NH), 8.26 (s, 1H, CH), 8.00 (d, *J* = 7.4 Hz, 2H, Ar), 7.69–7.65 (m, 1H, Ar), 7.51 (t, *J* = 7.4 Hz, 1H, Ar), 7.32 (t, *J* = 7.4 Hz, 2H, Ar), 7.29–7.19 (m, 3H, Ar), 6.89 (d, *J* = 2.9 Hz, 1H, Ar), 4.76 (s, 1H, NH), and 1.43 (s, 9H, 3CH_3_). ^13^C NMR (100 MHz, CDCl_3_): δ 193.4, 137.5, 136.4, 134.3, 132.6, 130.1 (2C), 129.2 (2C), 128.9, 128.6, 128.2, 123.4, 122.0, 118.4, 111.9, 109.5. 55.1, and 29.9 (3C). HRMS (ESI) *m*/*z* [M + H]^+^: calculated for C_21_H_23_N_2_O_3_S 383.1428, found 383.1453.

(*E*)-1-(1-Acetyl-1*H*-indol-3-yl)-*N*-(*tert*-butyl)-3-oxo-3-phenylprop-1-ene-2-sulfonamide (**10l**)

This compound was prepared from **6** and 1-acetyl-1*H*-indole-3-carbaldehyde as described for **10a**. The yield was 116 mg (55%) of colorless powder. ^1^H NMR (400 MHz, CDCl_3_): δ 8.33 (d, *J* = 7.4 Hz, 1H, Ar), 8.12 (s, 1H, CH), 8.06 (d, *J* = 8.0 Hz, 2H, Ar), 7.69 (d, *J* = 7.4 Hz, 1H, Ar), 7.58 (t, *J* = 7.4 Hz, 1H, Ar), 7.47–7.37 (m, 4H, Ar), 7.05 (s, 1H, Ar), 4.62 (s, 1H, NH), 2.22 (s, 3H, CH_3_), and 1.43 (s, 9H, 3CH_3_). ^13^C NMR (100 MHz, CDCl_3_): δ 192.2, 168.6 139.3, 136.8, 136.4, 135.7, 129.8 (2C), 129.4 (2C), 128.7, 127.8, 127.3, 126.7, 124.8, 118.6, 116.6, 112.8, 55.5, 29.9 (3C), and 23.6. HRMS (ESI) [M + H]^+^: calculated for C_23_H_25_N_2_O_4_S 425.1530 found 425.1546.

(*E*)-3-Oxo-1,3-diphenylprop-1-ene-2-sulfonamide (**7a**)

To a stirring solution of (*E*)-*N*-(*tert*-butyl)-3-oxo-1,3-diphenylprop-1-ene-2-sulfonamide (**10a**, 80 mg, 0.20 mmol) in CH_2_Cl_2_ (4.0 mL), TFA (1.0 mL) was added at room temperature. The resulting mixture was stirred for 4 h. Upon completion, the solution was concentrated in vacuo, diluted with toluene (5 mL), and concentrated in vacuo again. The residue was crystallized from a mixture of CHCl_3_-hexane. The yield was 60 mg (90%) of colorless powder, mp 140–142 °C. ^1^H NMR (400 MHz, CDCl_3_): δ 7.96 (s, 1H, CH), 7.88 (d, *J* = 7.8 Hz, 2H, Ar), 7.48 (t, *J* = 7.4 Hz, 1H, Ar), 7.32 (t, *J* = 7.4 Hz, 2H, Ar), 7.25–7.15 (m, 5H, Ar), and 5.12 (s, 2H, NH_2_). ^13^C NMR (100 MHz, CDCl_3_): δ 193.7, 139.3, 138.6, 134.9, 134.7, 131.4, 130.8, 130.1 (2C), 129.9 (2C), and 128.8 (4C). HRMS (ESI) *m*/*z* [M + Na]^+^: calculated for C_15_H_13_NNaO_3_S 310.0514, found 310.0520. HPLC (LW= 260 nm, A—H_3_PO_4_ (0.01 M) pH = 2.6, B—MeCN; gradient B 40→90%, 30 min): *t*_R_ = 12.2 min, purity 99%.

(*E*)-1-(2-Fluorophenyl)-3-oxo-3-phenylprop-1-ene-2-sulfonamide (**7b**)

This compound was prepared from **10b** as described for **7a**. The yield was 63 mg (91%) of colorless powder, 143–145 mp °C. ^1^H NMR (400 MHz, CDCl_3_): δ 8.13 (s, 1H, CH), 7.84 (d, *J* = 7.4 Hz, 2H, Ar), 7.47 (t, *J* = 7.4 Hz, 1H, Ar), 7.31 (t, *J* = 7.4 Hz, 2H, Ar), 7.25–7.17 (m, 1H, Ar), 7.09 (t, *J* = 7.0 Hz, 1H, Ar), 6.94 (t, *J* = 7.4 Hz, 1H, Ar), 6.88 (t, *J* = 7.4 Hz, 1H, Ar), and 4.18 (s, 2H, NH_2_). ^13^C NMR (100 MHz, CDCl_3_): δ 193.0, 160.5, (d, *J* = 252.7 Hz, C-F), 140.3, 134.9, 134.6, 132.8 (d, *J* = 8.8 Hz), 132.3 (d, *J* = 2.9 Hz), 130.5, 129.7 (2C), 128.7 (2C), 124.3 (d, *J* = 2.9 Hz), 119.9 (d, *J* = 12.6 Hz), and 115.9 (d, *J* = 22.8 Hz). HRMS (ESI) (*m*/*z*) [M – H]^−^: calculated for C_15_H_11_FNO_3_S 304.0449, found 304.0297. HPLC (LW= 260 nm, A—H_3_PO_4_ (0.01 M) pH = 2.6, B—MeCN; gradient B 40→90%, 30 min): *t*_R_ = 12.2 min, purity 96%.

(*E*)-1-(3-Fluorophenyl)-3-oxo-3-phenylprop-1-ene-2-sulfonamide (**7c**)

This compound was prepared from **10c** as described for **7a**. The yield was 62 mg (90%) of colorless powder, mp 110–112 °C. ^1^H NMR (400 MHz, CDCl_3_): *δ* 7.88 (s, 1H, CH), 7.86 (d, *J* = 7.8 Hz, 2H, Ar), 7.49 (t, *J* = 7.4 Hz, 1H, Ar), 7.33 (t, *J* = 7.8 Hz, 2H, Ar), 7.19–7.10 (m, 1H, Ar), 6.98 (d, *J* = 7.8 Hz, 1H, Ar), 6.96–6.86 (m, 2H, Ar), and 5.27 (s, 2H, NH_2_). ^13^C NMR (100 MHz, CDCl_3_): *δ* 193.3, 162.4 (d, *J* = 253.8 Hz, C-F), 140.0, 137.6, 134.9, 134.7, 133.4 (d, *J* = 7.8 Hz), 130.5 (d, *J* = 7.8 Hz), 129.9 (2C), 128.9 (2C), 125.8 (d, *J* = 2.8 Hz), 117.7 (d, *J* = 22.1 Hz), and 116.5 (d, *J* = 22.1 Hz). HRMS (ESI) (*m*/*z*) [M − H]^−^: calculated for C_15_H_11_FNO_3_S 304.0449, found 304.0485. HPLC (LW= 260 nm, A—H_3_PO_4_ (0.01 M) pH = 2.6, B—MeCN; gradient B 40→90%, 30 min): *t*_R_ = 12.7 min, purity 99%.

(*E*)-1-(4-Fluorophenyl)-3-oxo-3-phenylprop-1-ene-2-sulfonamide (**7d**)

This compound was prepared from **10d** as described for **7a**. The yield was 64 mg (91%) of colorless powder, mp 191–193 °C. ^1^H NMR (400 MHz, CDCl_3_): *δ* 7.92 (s, 1H, CH), 7.88 (d, *J* = 7.8 Hz, 2H, Ar), 7.52 (t, *J* = 7.0 Hz, 1H, Ar), 7.35 (t, *J* = 7.8 Hz, 2H, Ar), 7.24–7.19 (m, 2H, Ar), 6.88 (d, *J* = 8.4 Hz, 2H, Ar), and 5.09 (s, 2H, NH_2_). ^13^C NMR (100 MHz, CDCl_3_): *δ* 188.8, 159.2 (d, *J* = 253.6 Hz, C-F), 133.7, 133.2, 130.1, 130.0, 127.5 (d, *J* = 8.3 Hz, 2C), 125.1 (2C), 124.2 (2C), 122.8 (d, *J* = 3.2 Hz), and 111.4 (d, *J* = 22.5 Hz, 2C). HRMS (ESI) (*m*/*z*) [M − H]^−^: calculated for C_15_H_11_FNO_3_S 304.0449, found 304.0450. HPLC (LW= 260 nm, A—H_3_PO_4_ (0.01 M) pH=2.6, B—MeCN; gradient B 40→90%, 30 min): *t*_R_ = 12.6 min, purity 98%.

(*E*)-1-(4-Chlorophenyl)-3-oxo-3-phenylprop-1-ene-2-sulfonamide (**7e**)

This compound was prepared from **10e** as described for **7a**. The yield was 90 mg (92%) of colorless powder, mp 171–173 °C. ^1^H NMR (400 MHz, CDCl_3_): δ 7.92–7.85 (m, 3H, CH, 2Ar), 7.52 (t, *J* = 7.0 Hz, 1H, Ar), 7.35 (t, *J* = 7.8 Hz, 2H, Ar), 7.18–7.13 (m, 4H, Ar), and 5.12 (s, 2H, NH_2_). ^13^C NMR (100 MHz, CDCl_3_): δ 193.4, 139.2, 137.6, 137.1, 134.9, 134.7, 131.2 (2C), 129.9 (2C), 129.4, 129.1 (2C), and 129.0 (2C). HRMS (ESI) *m*/*z* [M − H]^−^: calculated for C_15_H_11_ClNO_3_S 320.0154, found 320.0046. HPLC (LW= 260 nm, A—H_3_PO_4_ (0.01 M) pH=2.6, B—MeCN; gradient B 40→90%, 30 min): *t*_R_ = 15.4 min, purity 99%.

(*E*)-3-Oxo-3-phenyl-1-(4-(trifluoromethyl)phenyl)prop-1-ene-2-sulfonamide (**7f**)

This compound was prepared from **10f** as described for **7a**. The yield was 97 mg (94%) of colorless powder, mp 95–97 °C. ^1^H NMR (400 MHz, CDCl_3_): δ 7.94 (s, 1H, CH), 7.86 (dd, *J*^1^ = 8.2, *J*^2^ = 1.3 Hz, 2H, Ar), 7.51 (t, *J* = 7.4 Hz, 1H, Ar), 7.43 (d, *J* = 8.2 Hz, 2H, Ar), 7.37–7.31 (m, 4H, Ar), and 5.18 (s, 2H, NH_2_). ^13^C NMR (100 MHz, CDCl_3_): δ 193.1, 141.2, 137.0, 135.1, 134.8, 134.6, 133.6 (q, *J* = 33.5 Hz, C-CF_3_), 130.1 (2C), 129.9 (2C), 129.0 (2C), 125.7 (2C), and 124.0 (q, *J* = 280.3 Hz, CF_3_). HRMS (ESI) *m*/*z* [M − H]^−^: calculated for C_16_H_11_F_3_NO_3_S 354.0417, found 354.0376. HPLC (LW = 260 nm, A—H_3_PO_4_ (0.01 M) pH=2.6, B—MeCN; gradient B 40→90%, 30 min): *t*_R_ = 16.4 min, purity 98%.

(*E*)-1-(4-Hydroxyphenyl)-3-oxo-3-phenylprop-1-ene-2-sulfonamide (**7g**)

This compound was prepared from **10g** as described for **7a**. The yield was 64 mg (89%) of colorless powder, mp 193–195 °C decomp. ^1^H NMR (400 MHz, DMSO-*d*_6_): δ 10.02 (s, 1H, OH), 7.89 (d, *J* = 7.4 Hz, 2H, Ar), 7.64–7.57 (m, 2H, CH, Ar), 7.47 (t, *J* = 7.4 Hz, 2H, Ar), 7.40 (s, 2H, NH_2_), 7.11 (d, *J* = 8.4 Hz, 2H, Ar), and 6.63 (d, *J* = 8.4 Hz, 2H, Ar). ^13^C NMR (100 MHz, DMSO-*d*_6_): δ 193.4, 160.0, 138.5, 136.5, 135.9, 134.8, 132.1 (2C), 129.9 (2C), 129.4 (2C), 123.0, and 116.3 (2C). HRMS (ESI) (*m*/*z*) [M + H]^+^: calculated for C_15_H_14_NO_4_S 304.0638, found 304.0643. HPLC (LW= 260 nm, A—H_3_PO_4_ (0.01 M) pH = 2.6, B—MeCN; gradient B 40→90%, 30 min): *t*_R_ = 12.4 min, purity 100%.

(*E*)-1-(4-Hydroxy-3-methoxyphenyl)-3-oxo-3-phenylprop-1-ene-2-sulfonamide (**7h**)

This compound was prepared from **10h** as described for **7a**. The yield was 61 mg (88%) of colorless powder, mp 223–225 °C. ^1^H NMR (400 MHz, CDCl_3_): δ 8.58 (s, 1H, OH), 7.73 (d, *J* = 7.4 Hz, 2H, Ar), 7.54 (s, 1H, CH), 7.30 (t, *J* = 7.4 Hz, 1H, Ar), 7.15 (t, *J* = 7.4 Hz, 2H, Ar), 6.56 (dd, *J^1^* = 8.2, *J^2^* = 1.9 Hz, 1H, Ar), 6.49 (d, *J* = 8.2 Hz, 1H, Ar), 6.38 (d, *J* = 1.9 Hz, 1H, Ar), and 6.19 (s, 2H, NH_2_). ^13^C NMR (100 MHz, CDCl_3_): δ 193.5, 149.1, 147.2, 138.3, 136.3, 135.4, 134.2, 129.6 (2C), 128.7 (2C), 125.2, 122.9, 115.4, 112.5, and 55.3. HRMS (ESI) (*m*/*z*) [M + H]^+^: calculated for C_16_H_16_NO_5_S 334.0744, found 334.0739. HPLC (LW = 260 nm, A—H_3_PO_4_ (0.01 M) pH = 2.6, B—MeCN; gradient B 40/90%, 30 min): *t*_R_ = 7.1 min, purity 100%.

(*E*)-1-(Furan-2-yl)-3-oxo-3-phenylprop-1-ene-2-sulfonamide (**7i**)

This compound was prepared from **10i** as described for **7a**. The yield was 89 mg (89%) of colorless powder, mp 135–137 °C. ^1^H NMR (400 MHz, CDCl_3_): δ 7.96 (d, *J* = 7.4 Hz, 2H, Ar), 7.62 (s, 1H, CH), 7.55 (t, *J* = 7.4 Hz, 1H, Ar), 7.41 (t, *J* = 7.4 Hz, 2H, Ar), 7.17 (s, 1H, Ar), 6.65 (d, *J* = 3.1 Hz, 1H, Ar), 6.36–6.31 (m, 1H, Ar), and 5.12 (s, 2H, NH_2_). ^13^C NMR (100 MHz, CDCl_3_): δ 192.7, 147.2, 146.6, 136.1, 134.6, 134.3, 129.7 (2C), 128.7 (2C), 125.0, 118.9, and 112.6. HRMS (ESI) *m*/*z* [M + H]^+^: calculated for C_13_H_12_NO_4_S 278.0482, found 278.0529. HPLC (LW = 260 nm, A—H_3_PO_4_ (0.01 M) pH = 2.6, B—MeCN; gradient B 40→90%, 30 min): *t*_R_ = 9.6 min, purity 99%.

(*E*)-3-Oxo-3-phenyl-1-(pyridin-4-yl)prop-1-ene-2-sulfonamide (**7j**)

This compound was prepared from **10j** as described for **7a**. The yield was 85 mg (93%) of colorless powder, mp 119–121 °C. ^1^H NMR (400 MHz, DMSO-*d*_6_): δ 8.65 (d, *J* = 5.5 Hz, 2H, Ar), 7.89 (br s, 5H, CH, NH_2_, Ar), 7.65 (t, *J* = 7.4 Hz, 1H, Ar), 7.54 (d, *J* = 5.5 Hz, 2H, Ar), and 7.49 (t, *J* = 7.4 Hz, 2H, Ar). ^13^C NMR (100 MHz, DMSO-*d*_6_): δ 191.6, 148.4, 146.6 (2C), 144.7, 135.5, 135.1, 132.4, 130.1 (2C), 129.6 (2C), and 125.2 (2C). HRMS (ESI) *m*/*z* [M + H]^+^: calculated for C_14_H_13_N_2_O_3_S 289.0641, found 289.0685. HPLC (LW = 260 nm, A—H_3_PO_4_ (0.01 M) pH = 2.6, B—MeCN; gradient B 40→90%, 30 min): *t*_R_ = 11.9 min, purity 100%.

(*E*)-1-(1*H*-Indol-3-yl)-3-oxo-3-phenylprop-1-ene-2-sulfonamide (**7k**)

This compound was prepared from **10k** as described for **7a**. The residue was purified with flash column chromatography (silica gel, hexane—EtOAc 2:1). The yield was 55 mg (43%) of yellow powder, mp 94–96 °C. ^1^H NMR (400 MHz, CDCl_3_): δ 8.57 (br s, 1H, NH), 8.24 (s, 1H, CH), 8.00 (d, *J* = 7.4 Hz, 2H, Ar), 7.76–7.72 (m, 1H, Ar), 7.49 (t, *J* = 7.4 Hz, 1H, Ar), 7.33 (t, *J* = 7.4 Hz, 2H, Ar), 7.25–7.21 (m, 2H, Ar), 7.18 (d, *J* = 7.4 Hz, 1H, Ar), 6.93 (d, *J* = 2.9 Hz, 1H, Ar), and 5.11 (s, 2H, NH_2_). ^13^C NMR (100 MHz, CDCl_3_): δ 194.6, 135.5, 134.8, 134.6, 132.5, 131.5, 130.0 (2C), 129.0 (2C), 128.3, 128.2, 123.6, 121.8, 118.6, 111.7, and 109.0. HRMS (ESI) *m*/*z* [M + H]^+^: calculated for C_17_H_15_N_2_O_3_S 327.0798, found 327.0802. HPLC (LW = 260 nm, A—H_3_PO_4_ (0.01 M) pH = 2.6, B—MeCN; gradient B 40→90%, 30 min): *t*_R_ = 10.6 min, purity 95%.

(*E*)-1-(1-Acetyl-1*H*-indol-3-yl)-3-oxo-3-phenylprop-1-ene-2-sulfonamide (**7l**)

This compound was prepared from **10l** as described for **7a**. The yield was 92 mg (91%) of yellowish powder, mp 103–105 °C. ^1^H NMR (400 MHz, CDCl_3_): δ 8.25 (d, *J* = 7.8 Hz, 1H, Ar), 8.02 (s, 1H, CH), 7.96 (d, *J* = 7.8 Hz, 2H, Ar), 7.61 (d, *J* = 7.0 Hz, 1H, Ar), 7.51 (t, *J* = 7.0 Hz, 1H, Ar), 7.39–7.29 (m, 4H, Ar), 6.97 (s, 1H, Ar), 5.38 (s, 2H, NH_2_), and 2.23 (s, 3H, CH_3_). ^13^C NMR (100 MHz, CDCl_3_): δ 193.9. 168.2, 137.9, 135.1 (2C), 134.6, 129.8 (2C), 129.2 (2C), 128.8, 128.5, 127.2, 126.4, 124.6, 118.5, 116.6, 113.6, and 23.5. HRMS (ESI) *m*/*z* [M + H]^+^: calculated for C_19_H_17_N_2_O_4_S 369.0904, found 369.0913. HPLC (LW= 260 nm, A—H_3_PO_4_ (0.01 M) pH = 2.6, B—MeCN; gradient B 40→90%, 30 min): *t*_R_ = 13.7 min, purity 95%.

2-Oxo-2-phenylethan-1-sulfonamide (**11**)

This compound was prepared from **6** as described for **7a**. The yield was 138 mg (93%) of colorless powder, mp 160–161 °C. ^1^H NMR (400 MHz, DMSO-*d*_6_): δ 8.02 (d, *J* = 7.4 Hz, 2H, Ar), 7.67 (t, *J* = 7.0 Hz, 1H, Ar), 7.54 (t, *J* = 7.4 Hz, 2H, Ar), 7.16 (s, 2H, NH_2_), and 4.80 (s, 2H, CH_2_). ^13^C NMR (100 MHz, DMSO-*d*_6_): δ 190.4, 136.3, 134.4, 129.7 (2C), 129.1 (2C), and 62.2. HRMS (ESI) *m*/*z* [M + H]^+^: calculated for C_8_H_10_NO_3_S 200.0376, found 200.0368. HPLC (LW = 260 nm, A—H_3_PO_4_ (0.01 M) pH = 2.6, B—MeCN; gradient B 40→90%, 30 min): *t*_R_ = 4.5 min, purity 99%.

### 4.2. X-ray Study of ***7a***

Single crystal X-ray studies of **7a** were carried out in the Center for molecule composition studies of INEOS RAS with APEX3 software v.2019.11-0 [56]. The data were then integrated with SAINT. SADABS was used for scaling, empirical absorption corrections, and the generation of data files for structure solution and refinement. 

The structures were solved with a dual-space algorithm and refined in anisotropic approximation for non-hydrogen atoms against F^2^(hkl). Hydrogen atoms of methyl, methylene, and aromatic fragments were calculated according to those idealized geometries and refined with constraints. All structures were solved with the ShelXT [57] program and refined with the ShelXL [58] program. Molecular graphics were drawn using the OLEX2 [59] program.

CCDC 2304141 contains the supplementary crystallographic data for **7a**. These data can be obtained free of charge from the Cambridge Crystallographic Data Centre via https://www.ccdc.cam.ac.uk/structures (accessed on 15 October 2023).

### 4.3. Antiproliferative Activity and Selectivity

Antiproliferative activity of the synthesized compounds **7a**–**l** and **11** was evaluated against MCF7, MCF7/HT2 (human breast cancer), K-562 (chronic myelogenous leukemia), K-562/4 (characterized by a high resistance index for doxorubicin; a kind gift of Dr. Alexander Shtil, Blokhin N.N. National Medical Research Center of Oncology, Moscow, Russia) [36,60], and HCT-116 (human colon carcinoma) cells via the MTT assay. The MCF7, K-562, and HCT-116 cell lines were obtained from the ATCC collection. MCF7/HT2 cell subline was obtained as described in our recent work [34]. The multidrug-resistant (MDR) subline K-562/4 was obtained via stepwise selection of K-562 cells for survival under continuous exposure to doxorubicin [60]. The selectivity of compounds **7e** and **11** were evaluated using the resazurin test with recommendations from [39,40,41]. Fibroblasts and the protocol for their cultivation were kindly provided by Pavel B. Kopnin [37,38]. Cells were cultured in vitro in DMEM medium (PanEco, Moscow, Russia) supplemented with 10% fetal bovine serum (HyClone, Logan, UT, USA), 50 U/mL penicillin, and 50 µg/mL streptomycin (PanEco, Moscow, Russia). Suspension cells (K-562 and K-562/4) were propagated in RPMI-1640 (PanEco, Moscow, Russia) with the same supplements. The incubation was performed at 37 °C, 5% CO_2_, and 80–90% relative humidity in the NU-5840E cell incubator (NuAire, Plymouth, MN, USA). Cells in the logarithmic phase were used in all experiments.

To determine the overall metabolic activity of the cells in the well, the MTT assay was used based on the reduction of the MTT reagent (3-(4,5-dimethylthiazol-2-yl)-2,5-diphenyltetrazolium bromide) in living cells to form violet formazan crystals insoluble in the culture medium; the assay was performed in a modified version as described previously [61]. The compounds were dissolved at a concentration of 10 mM in DMSO (AppliChem, Germany, Darmstadt) and stored at −20 °C. The cells were seeded on a 24-well plate (Corning, NY, USA) and the compounds were added within 24 h at concentrations in the range from 1 to 50 µM; doxorubicin was used as a reference agent. The appropriate volume of the solvent was added to the control cells; the final concentration of the solvent in the medium did not exceed 0.5%.

After 72 h of growth in the presence of the compounds, the medium was removed (suspension cells K562 and K-562/4 were precentrifuged on a plate centrifuge (Thermo Fisher Scientific, Waltham, MA, USA)) and the MTT reagent (AppliChem, Darmstadt, Germany) dissolved in medium was added to the cells. After 2 h of incubation, the MTT solution was removed and DMSO was added, and the plates were gently shaken to dissolve the formed formazan crystals. The absorbance of the solutions was measured on a MultiSkan FC spectrophotometer (Thermo Fisher Scientific, Waltham, MA, USA) at 571 nm. Then the blank absorbance values (from wells without cells) were subtracted from the sample absorbance values; the absorbance of control samples was taken as 100%. The IC_50_ values were calculated using GraphPad 7.0 (Boston, MA, USA) as the concentration of the compound that decreased the absorbance of the solution by 50% compared to the control sample. More precisely, the IC_50_ values were determined using a nonlinear regression of the log(inhibitor) vs. normalized response with a variable slope (four parameters).

Statistical analysis was performed using Microsoft Excel and GraphPad Prism 7.0 (Boston, MA, USA). Each biology experiment was repeated three times, and the results were expressed as mean ± S.D. (standard deviation value). The Student’s *t*-test was used to evaluate the significance of differences in comparisons. A *p*-value of <0.05 was considered statistically significant. 

### 4.4. Immunoblotting

MCF7 and MCF7/HT2 cells were seeded on 100 mm dishes (Corning, NY, USA), and after 24 h of growth, compounds **7e** and **11** were added in a fresh medium. The cells were harvested after 24 h of incubation with the compounds. To prepare cell extracts, the cells were twice washed in phosphate buffer and incubated for 10 min on ice in the modified lysis buffer containing 50 mM Tris-HCl, pH 7.5, 0.5% Igepal CA-630, 150 mM NaCl, 1 mM EDTA, 1 mM DTT, 1 mM PMSF, 0.1 mM sodium orthovanadate and aprotinin, leupeptin, and pepstatin (1 µg/mL each) as described earlier [62]. The protein content was determined using the Bradford method [63].

Cell lysates were separated in 10% SDS-PAGE under reducing conditions, transferred to a nitrocellulose membrane (GE HealthCare, Addison, IL, USA,), and processed according to a standard protocol. To prevent nonspecific absorption, the membranes were treated with 5% nonfat milk solution in a TBS buffer (20 mM Tris, 500 mM NaCl, and pH 7.5) with 0.1% Tween-20 and then incubated with primary antibodies overnight at 4 °C.

ERα, GREB1, AR, cyclin D1, CDK4, p-AKT, AKT, cleaved PARP, and Bcl-2 antibodies were obtained from Cell Signaling Technology (Danvers, MA, USA); the antibodies against GAPDH (Cell Signaling Technology, Danvers, USA, MA) were added to standardize loading. Goat antirabbit IgGs (Jackson ImmunoResearch, West Grove, PA, USA) conjugated to horseradish peroxidase were used as secondary antibodies. Signals were detected using the ECL reagent as described in Mruk and Cheng’s protocol [64] and an ImageQuant LAS4000 system (GE HealthCare, IL, USA).

## 5. Conclusions

In conclusion, this research provides valuable insights into the design, synthesis, and evaluation of chalconesulfonamides as promising anticancer agents, indicating their potential for targeting hormone-dependent breast cancer cells. Compound **7e** suppresses all cancer cell lines with IC_50_ values in the low micromolar range and retains activity against resistant cell lines. Further research could focus on optimizing the structure of the sulfonamide moiety and development of novel combinational strategies for the treatment of breast cancer and other hormone-dependent tumors.

## Figures and Tables

**Table 1:** Antiproliferative potency of compounds **7a**–**l** and **11** with doxorubicin (Dox) as a reference drug (MTT assay after 72 h exposure; IC_50_, µM).

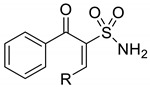						
Cmpd	R	IC_50_ (µM) ^a^				
		MCF7	MCF7/HT2	K-562	K-562/4	HCT-116
**7a**	** 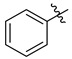 **	3.6 ± 0.2	3.5 ± 0.2	3.7 ± 0.3	12.5 ± 1.2	10.3 ± 1.1
**7b**	** 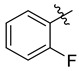 **	4.0 ± 0.3	4.5 ± 0.4	2.5 ± 0.3	6.4 ± 0.5	8.2 ± 0.8
**7c**	** 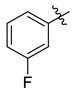 **	4.0 ± 0.3	3.9 ± 0.4	2.2 ± 0.1	11.5 ± 1.5	10.3 ± 1.1
**7d**	** 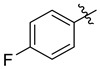 **	3.9 ± 0.2	4.8 ± 0.4	2.0 ± 0.1	15.3 ± 1.1	9.8 ± 1.1
**7e**	** 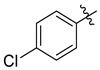 **	2.0 ± 0.1	3.8 ± 0.2	1.3 ± 0.1	2.8 ± 0.2	5.1 ± 0.4
**7f**	** 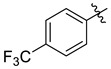 **	2.2 ± 0.3	2.5 ± 0.2	1.8 ± 0.2	2.8 ± 0.3	3.6 ± 0.3
**7g**	** 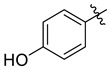 **	5.5 ± 0.7	7.3 ± 0.7	3.2 ± 0.2	18.5 ± 1.4	15.0 ± 1.4
**7h**	** 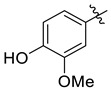 **	6.8 ± 0.3	8.8 ± 0.8	4.3 ± 0.6	32.0 ± 4.2	23.0 ± 3.0
**7i**	** 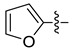 **	39.9 ± 3.3	35.9 ± 3.1	11.5 ± 1.2	NT	45.0 ± 7.0
**7j**	** 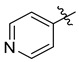 **	5.7 ± 0.3	5.1 ± 0.3	7.5 ± 0.8	NT	21.0 ± 2.5
**7k**	** 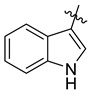 **	19.9 ± 1.4	14.1 ± 1.1	9.2 ± 1.1	NT	23.2 ± 2.5
**7l**	** 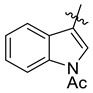 **	6.1 ± 0.4	6.7 ± 0.4	6.6 ± 0.7	NT	7.1 ± 0.8
**11**	** 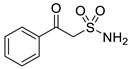 **	>50	>50	>50	>50	>50
Dox** ^b^**		0.31 ± 0.04	NT	0.12 ± 0.01	5.1 ± 0.5	0.32 ± 0.04

^a^ Mean ± S.D. of 3 independent experiments. ^b^ Dox, a reference drug. NT—not tested.

**Table 2:** Cytotoxicity and selectivity of compounds **7e** and **11** (the resazurin assay after 72 h exposure).

Cell Line		Compounds	
		7e	11
IC_50_, µM	MCF7 cells	2.6 ± 0.2	>50
	Fibroblasts	4.9 ± 0.3	>50
SI *		1.9	-

* SI—selectivity index = IC_50_ value for fibroblasts/IC_50_ value for MCF7 cells.

## Data Availability

Supplementary material contains 1H and 13C NMR spectra. For all final compounds HPLC results are provided.

## Supplementary Materials

The following supporting information can be downloaded at: https://www.mdpi.com/article/10.3390/ph17010032/s1. Figure S1. Centrosymmetric dimer in crystal structure of **7a** formed by hydrogen bonds between sufonamide groups; Figure S2. Centrosymmetric dimer in crystal structure of **7a** formed by hydrogen bonds between sufonamide and carbonyl groups; Figure S3. Crystal packing of **7a**; Table S1. Crystallographic data for **7a**; Table S2. Hydrogen Bonds for **7a**.

## Abbreviations

HRMS	High-resolution mass spectrometry;
HPLC	High-performance liquid chromatography;
LDA	Lithium diisopropylamide;
NMR	Nuclear magnetic resonance;
PG	Protecting group;
TFA	Trifluoroacetic acid.
ER α	Estrogen receptor α
AI	Aromatase inhibitor
SERM	Selective estrogen receptor modulator
SI	Selectivity index
GAPDH	Glyceraldehyde-3-phosphate dehydrogenase
GREB1	Gene regulated in breast cancer 1 protein
AR	Androgen receptor
CDK4	Cyclin-dependent kinase 4
PARP	Poly (ADP-ribose) polymerase 1
Bcl-2	B-cell CLL/lymphoma 2
HT	4-hydroxytamoxifen
MDR	Multidrug-resistant
