# Supplementary material for: Synthesis and Biological Evaluation of Chalconesulfonamides: En Route to Proapoptotic Agents with Antiestrogenic Potency
Source: Pharmaceuticals (Basel). 2023 Dec 25;17(1):32. doi: 10.3390/ph17010032 (PMC10818622; doi:10.3390/ph17010032)
Supplement: Supplementary file 1 [file pharmaceuticals-17-00032-s001.zip › pharmaceuticals-2721085-supplementary.pdf]

Supplementary material for the article

Synthesis and Biological Evaluation of Chalconesulfonamides: En Route to Proapoptotic Agents with Antiestrogenic Potency

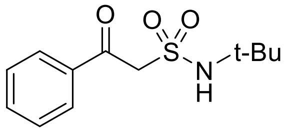

N-(*tert*-butyl)-2-oxo-2-phenylethane-1-sulfonamide (**6**)

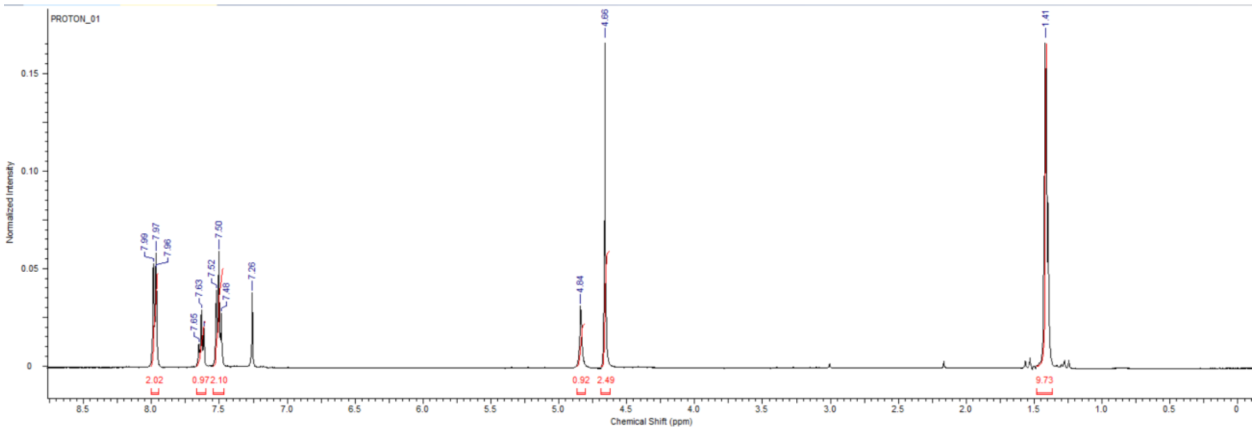

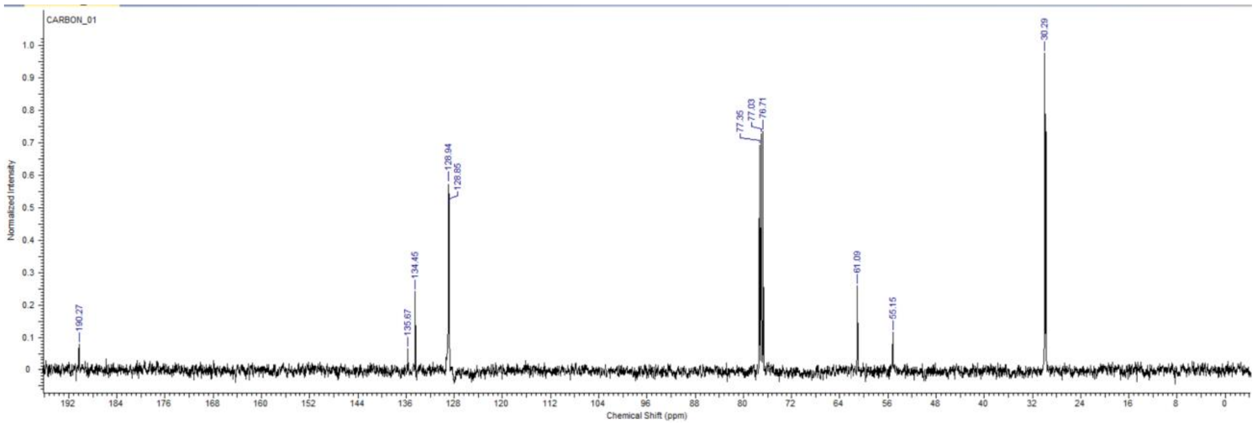

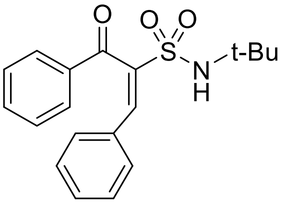

(*E*)-*N*-(*tert*-butyl)-3-oxo-1,3-diphenylprop-1-ene-2-sulfonamide (**10a**)

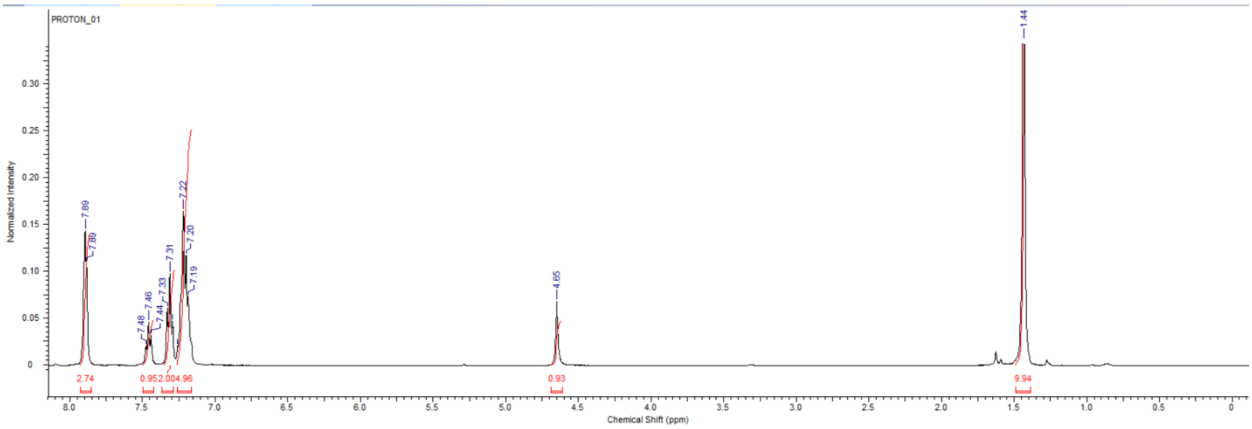

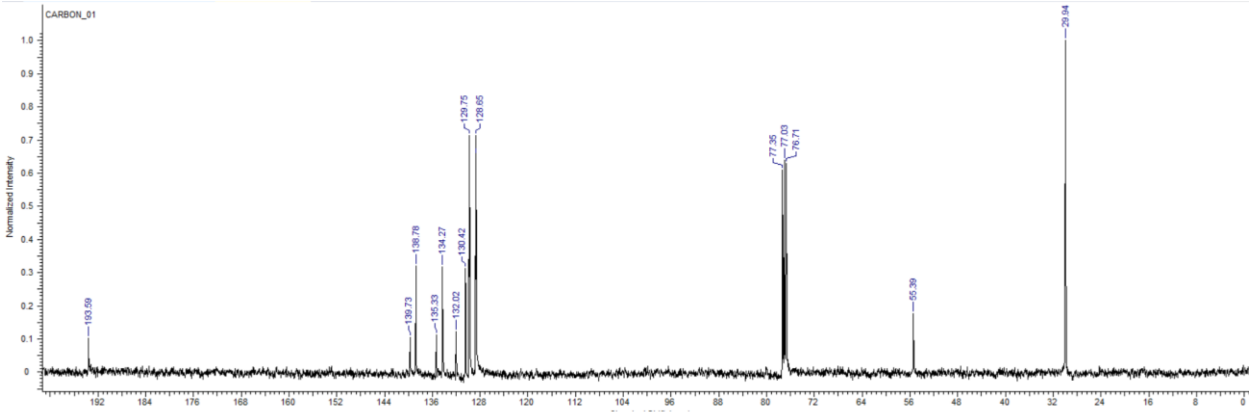

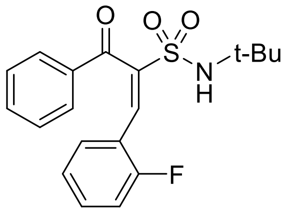

(*E*)-*N*-(*tert*-butyl)-1-(2-fluorophenyl)-3-oxo-3-phenylprop-1-ene-2-sulfonamide (**10b**)

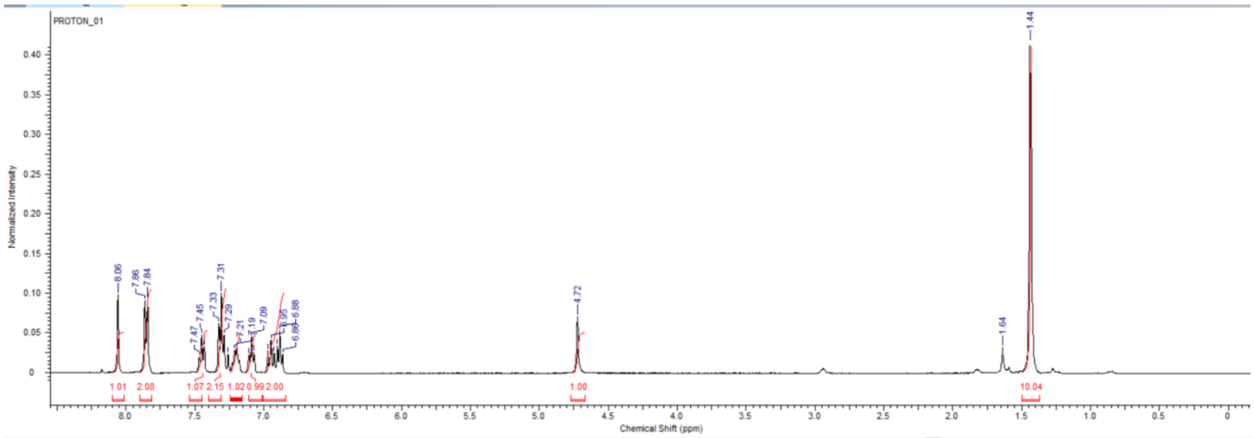

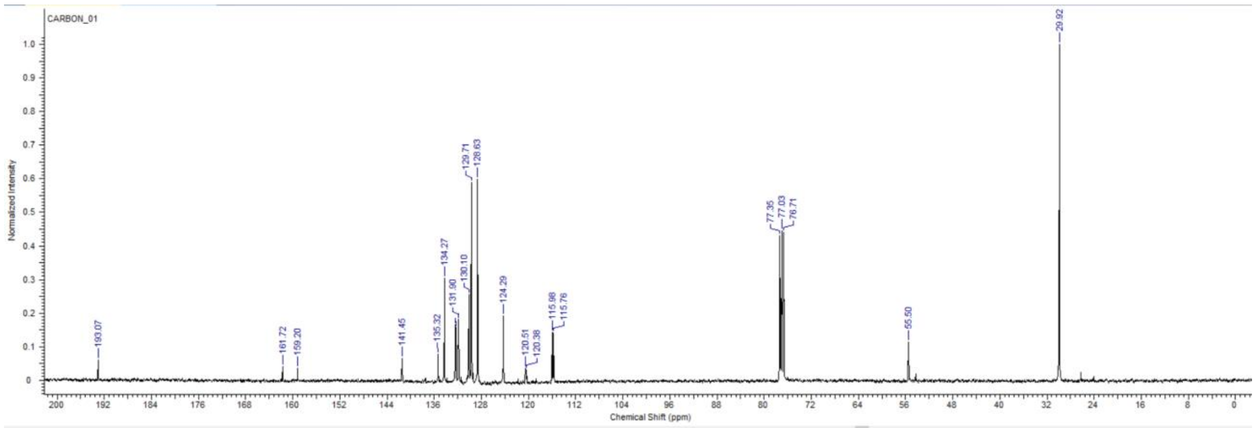

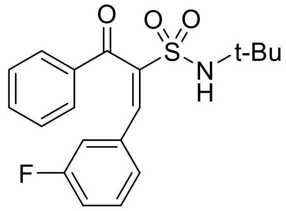

(*E*)-*N*-(*tert*-butyl)-1-(3-fluorophenyl)-3-oxo-3-phenylprop-1-ene-2-sulfonamide (**10c**)

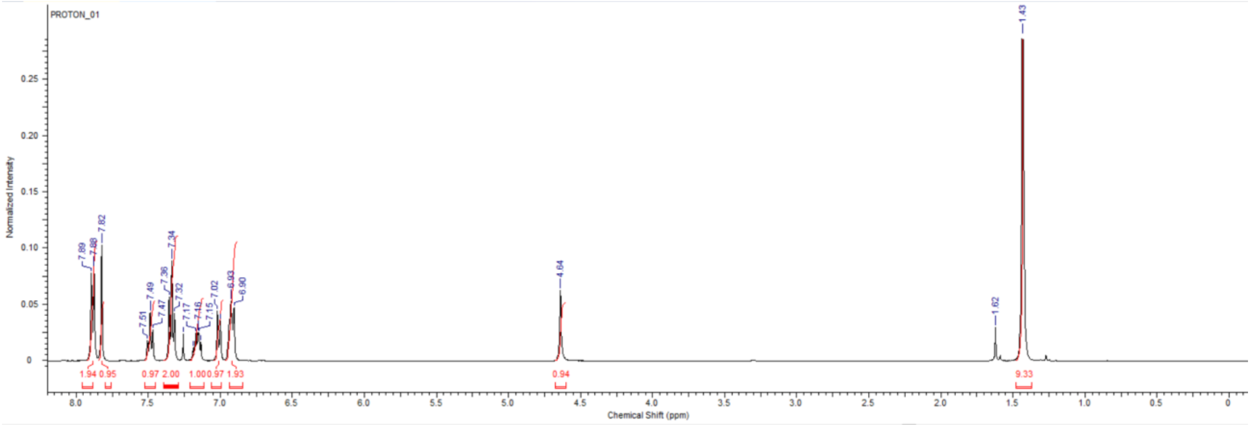

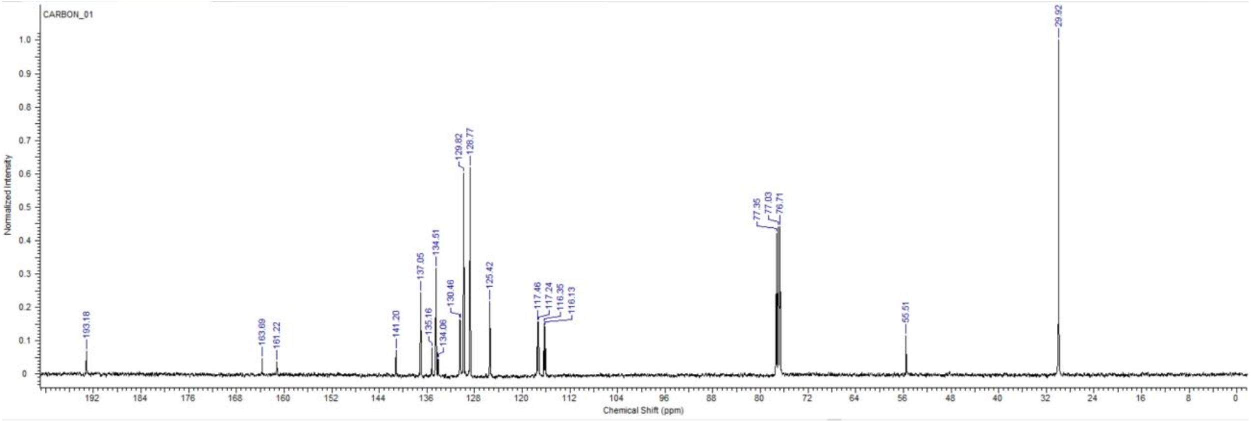

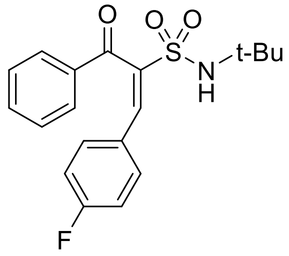

(*E*)-*N*-(*tert*-butyl)-1-(4-fluorophenyl)-3-oxo-3-phenylprop-1-ene-2-sulfonamide (**10d**)

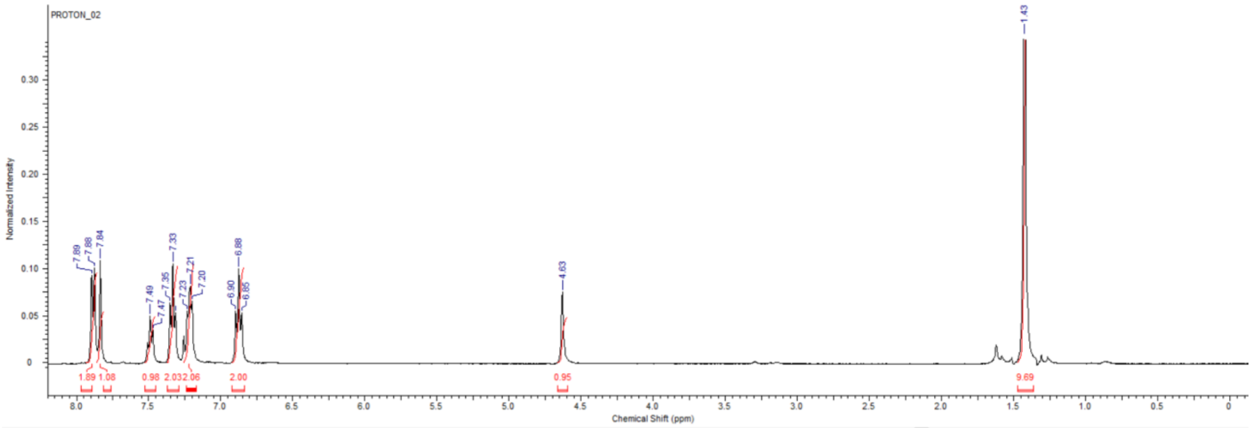

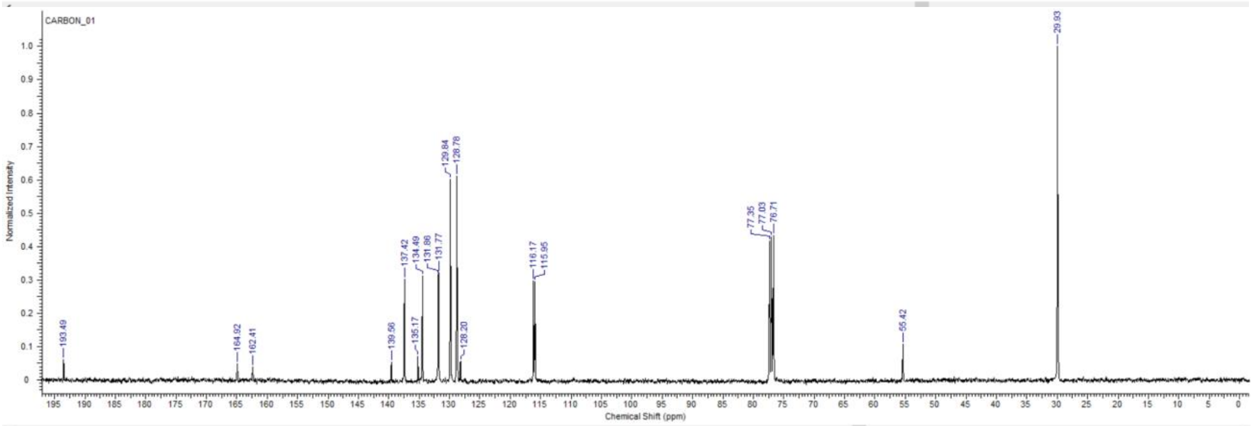

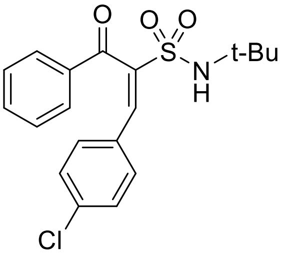

(*E*)-*N*-(*tert*-butyl)-1-(4-chlorophenyl)-3-oxo-3-phenylprop-1-ene-2-sulfonamide (**10e**)

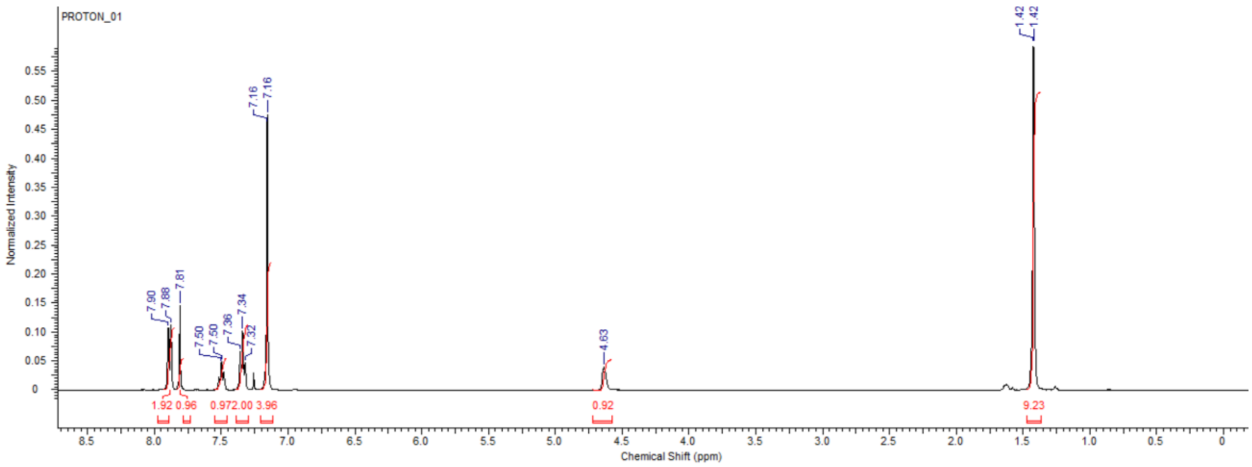

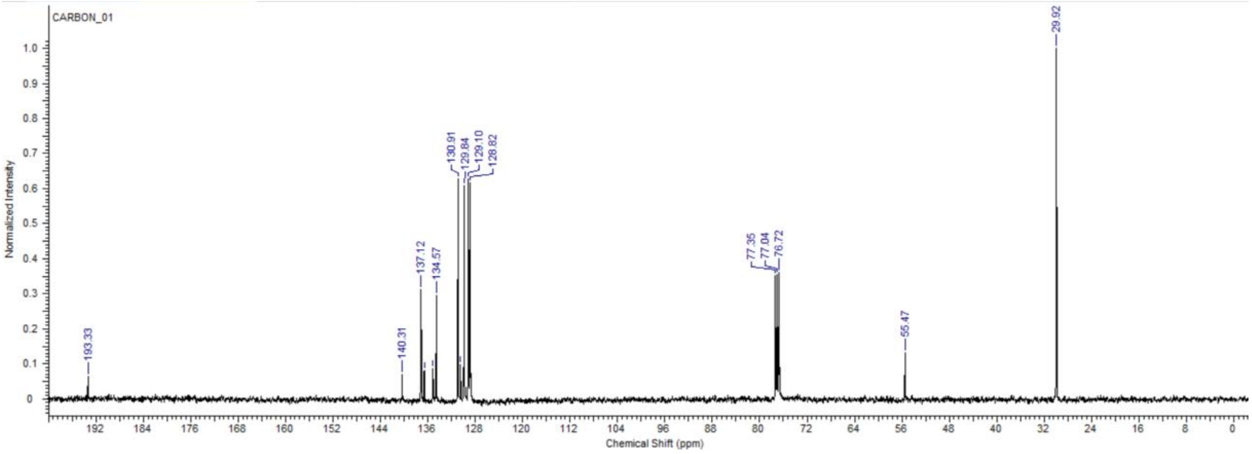

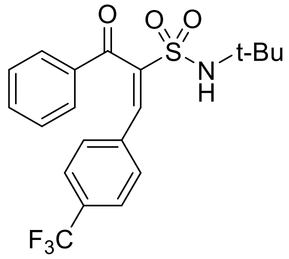

(*E*)-*N*-(*tert*-butyl)-3-oxo-3-phenyl-1-(4-(trifluoromethyl)phenyl)prop-1-ene-2-sulfonamide (**10f**)

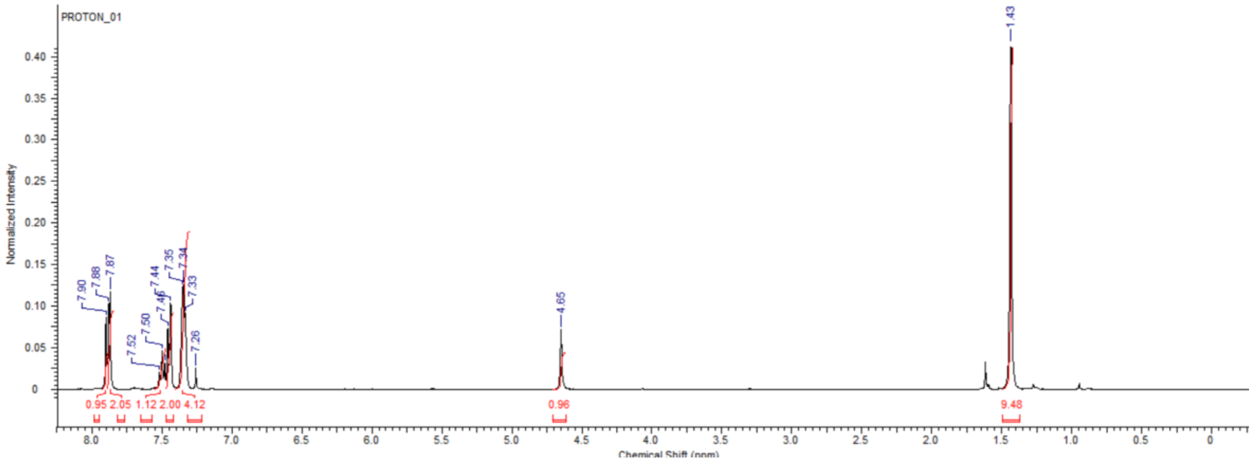

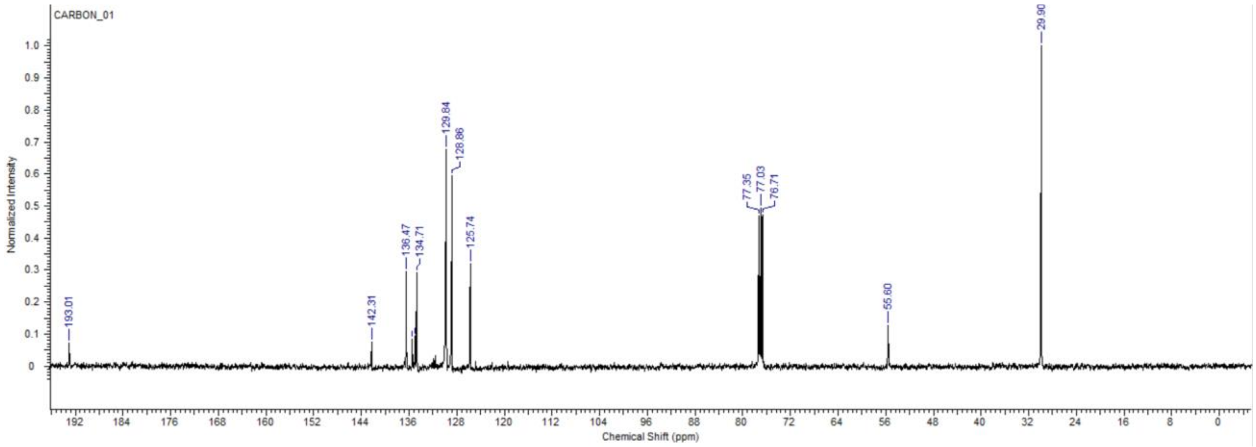

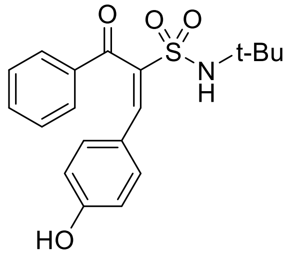

(*E*)-*N*-(*tert*-butyl)-1-(4-hydroxyphenyl)-3-oxo-3-phenylprop-1-ene-2-sulfonamide (**10g**)

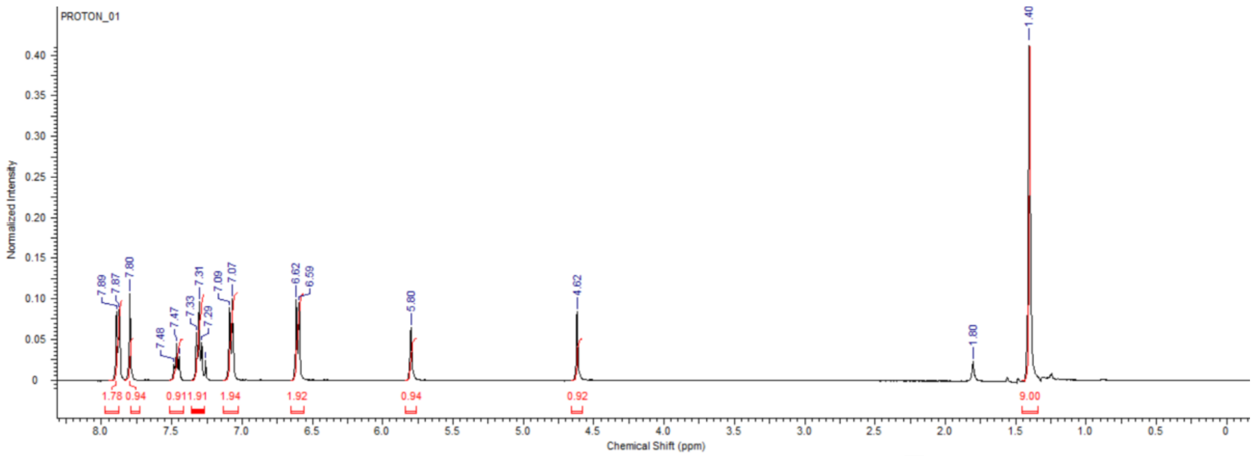

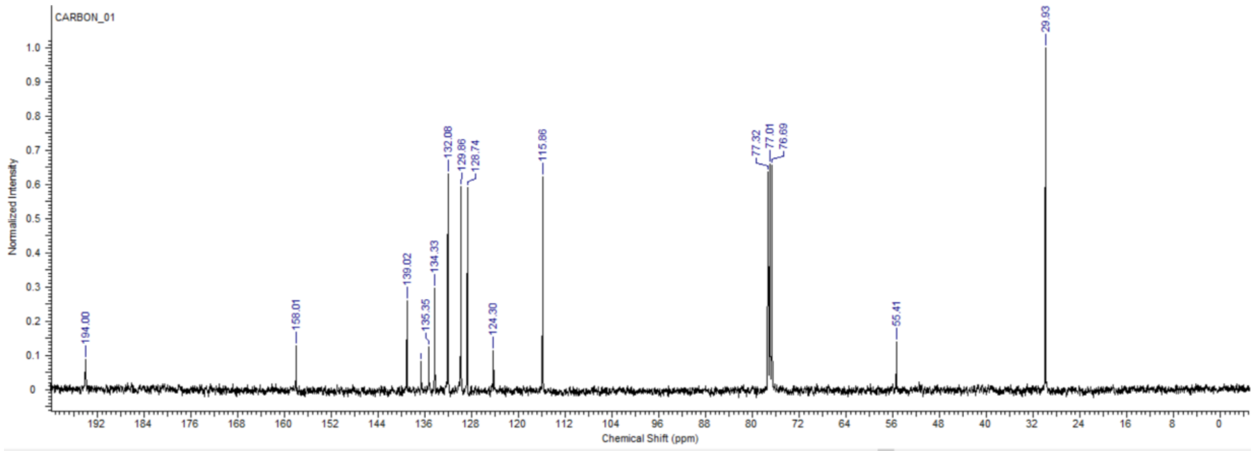

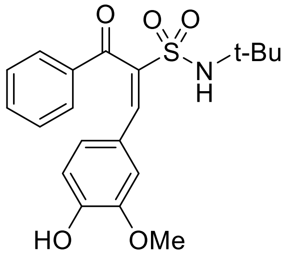

(*E*)-*N*-(*tert*-butyl)-1-(4-hydroxy-3-methoxyphenyl)-3-oxo-3-phenylprop-1-ene-2-sulfonamide (**10h**)

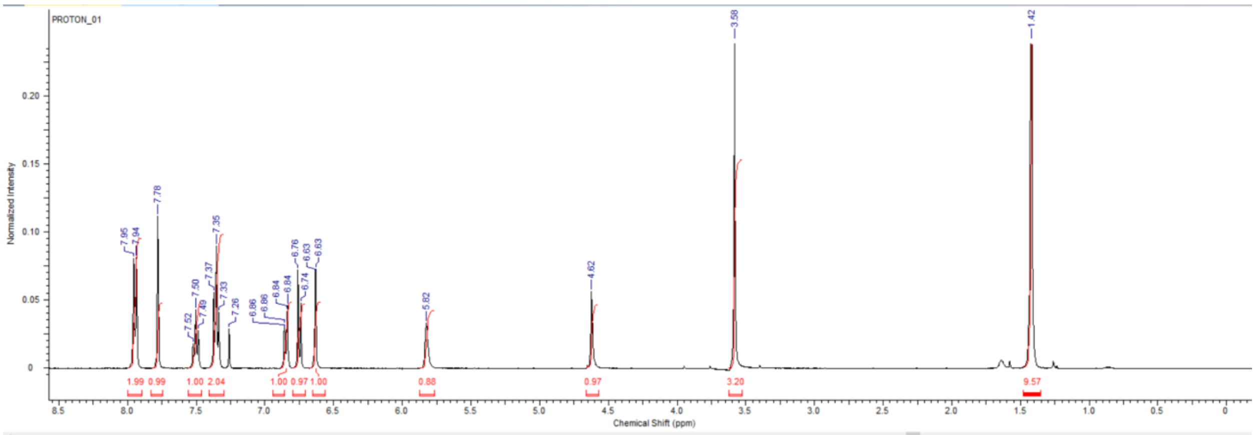

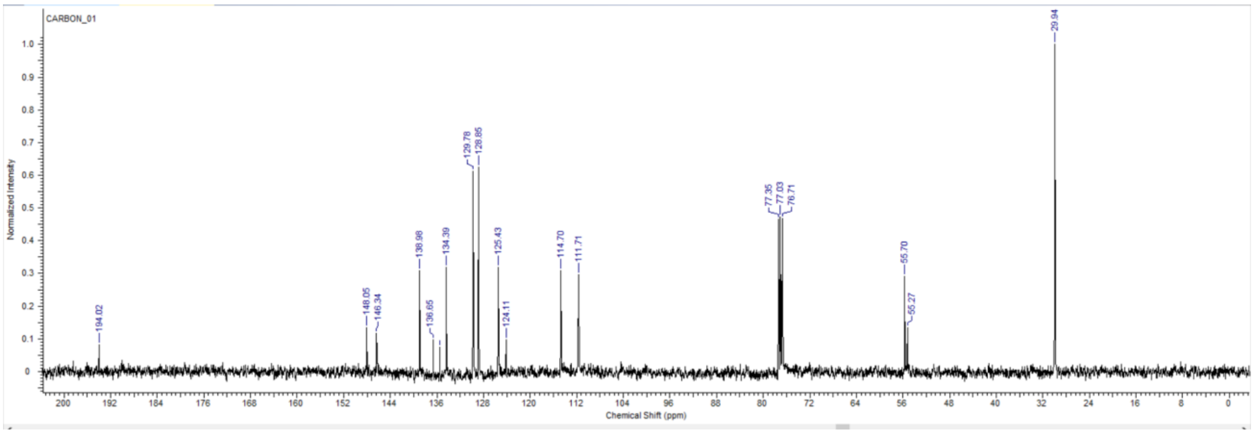

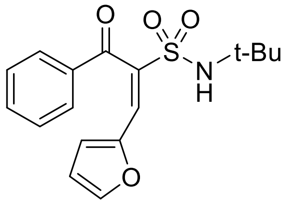

(*E*)-*N*-(*tert*-butyl)-1-(furan-2-yl)-3-oxo-3-phenylprop-1-ene-2-sulfonamide (**10i**)

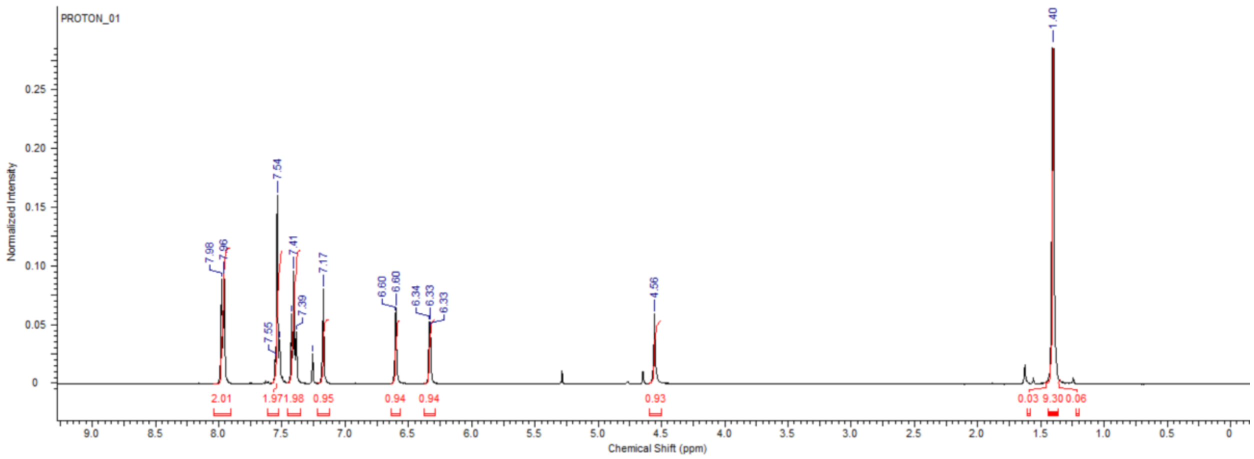
PROTON_01
Normalized Intensity
Chemical Shift (ppm)
7.98
7.96
7.55
7.54
7.41
7.39
7.17
6.60
6.60
6.34
6.33
6.33
4.56
1.40
2.01
1.97
1.98
0.95
0.94
0.94
0.93
0.03
9.30
0.06

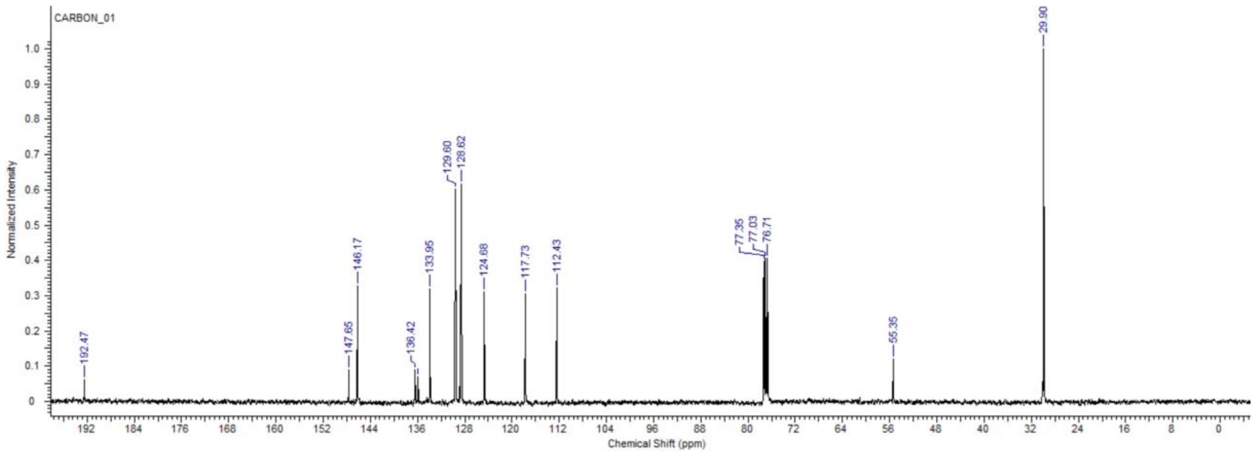
CARBON_01
Normalized Intensity
Chemical Shift (ppm)
192.47
147.65
146.17
136.42
133.95
129.80
128.62
124.68
117.73
112.43
77.35
77.03
76.71
55.35
29.90

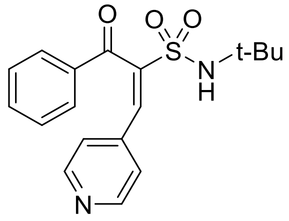

(E)-*N*-(*tert*-butyl)-3-oxo-3-phenyl-1-(pyridin-4-yl)prop-1-ene-2-sulfonamide (**10j**)

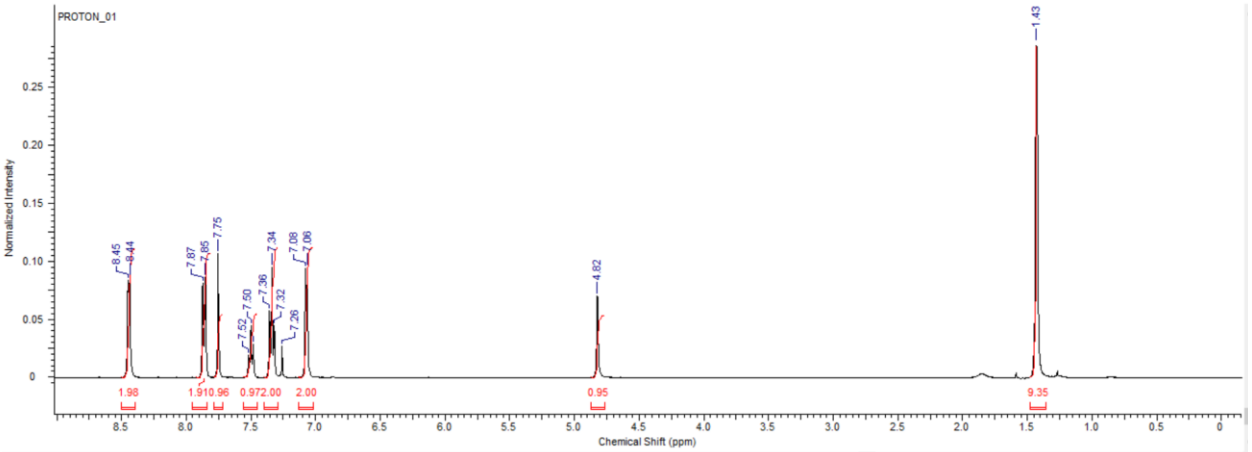

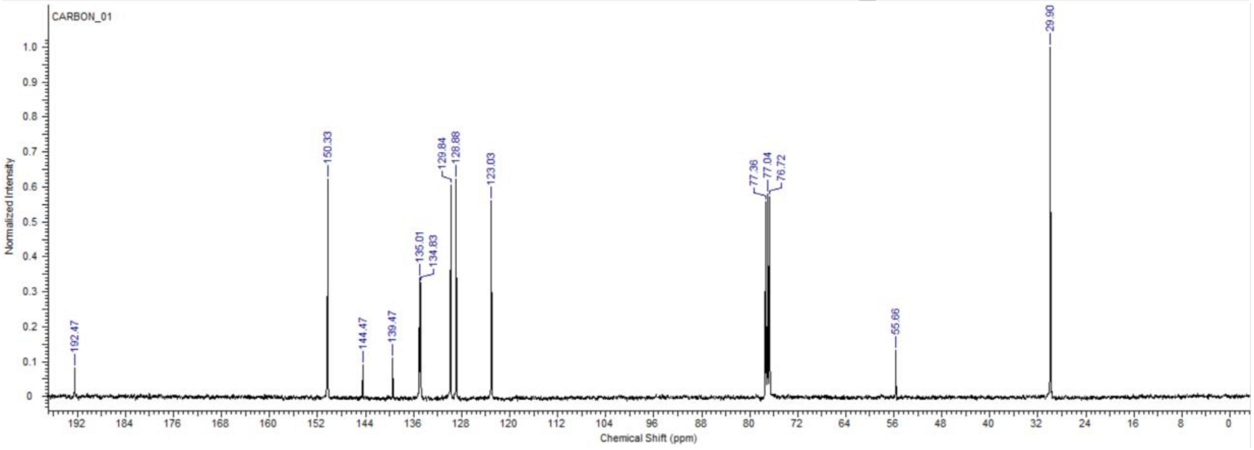

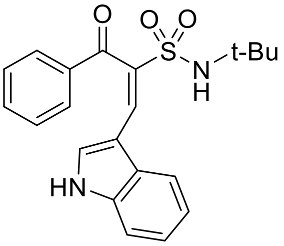

(*E*)-1-(1*H*-Indol-3-yl)- *N*-(*tert*-butyl)-3-oxo-3-phenylprop-1-ene-2-sulfonamide (**10k**).

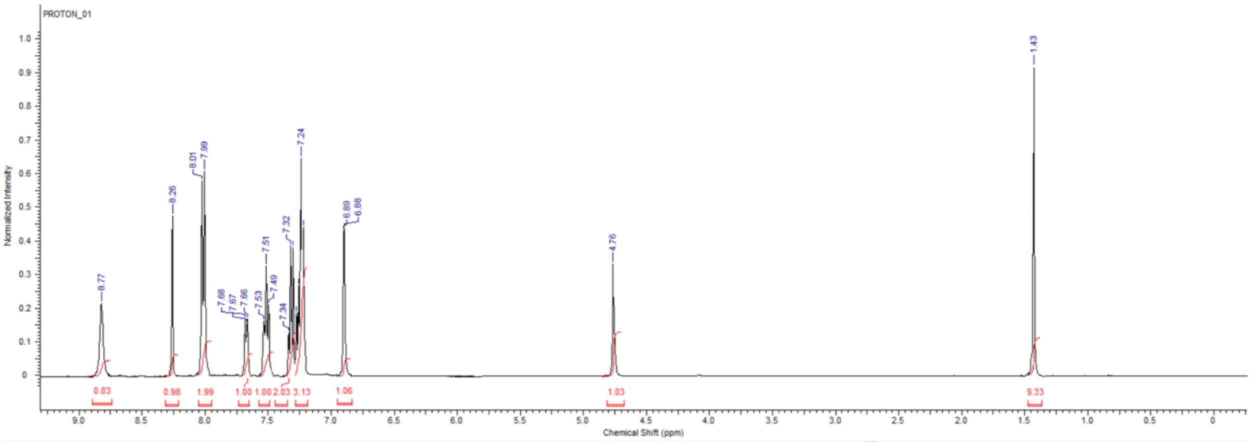

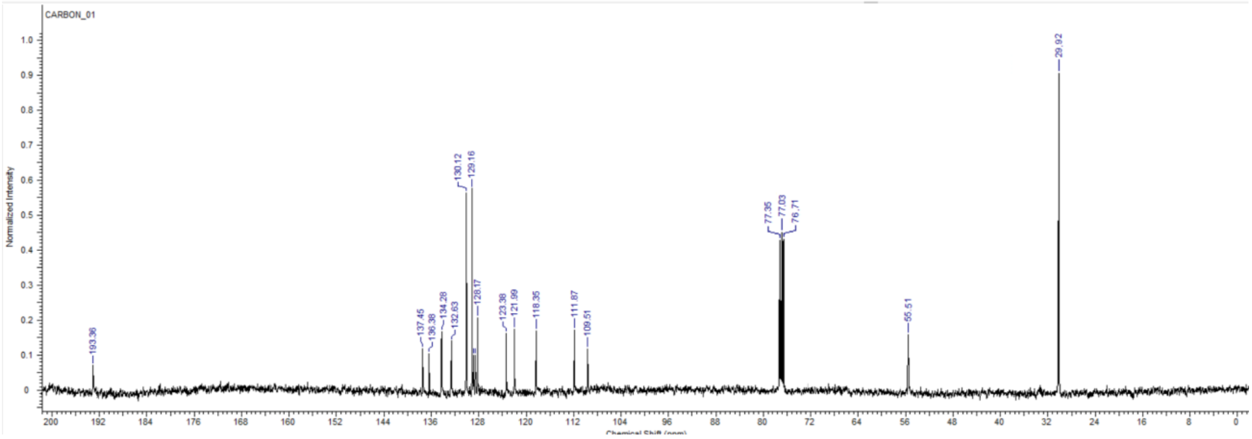

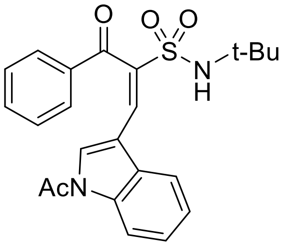

(E)-1-(1-Acetyl-1*H*-indol-3-yl)-N-(*tert*-butyl)-3-oxo-3-phenylprop-1-ene-2-sulfonamide (**10l**)

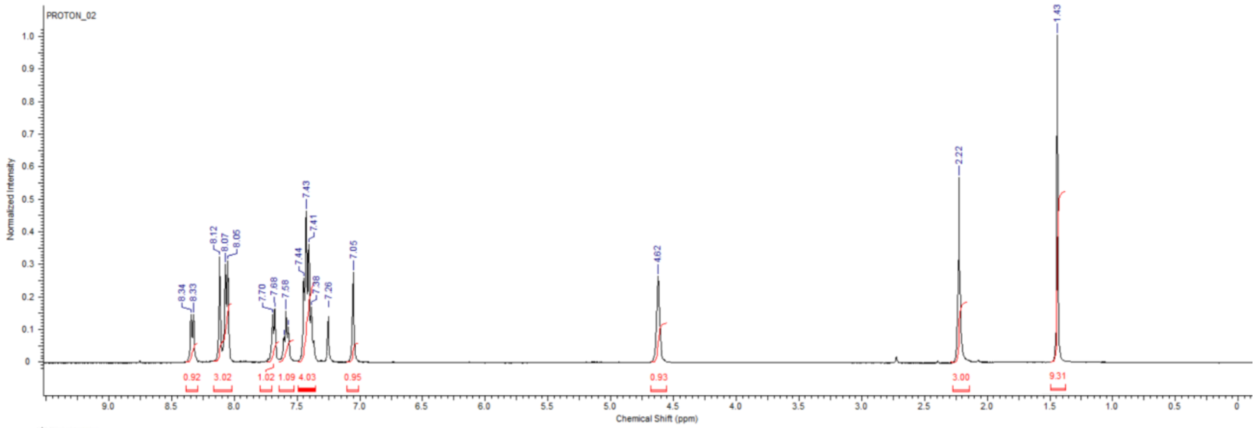

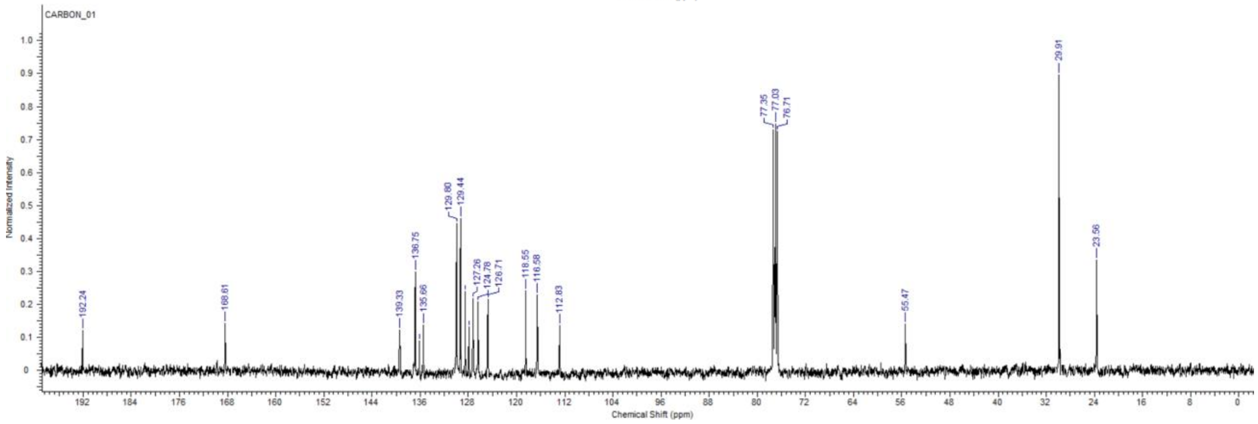

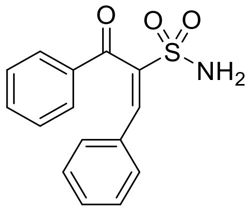

(E)-3-oxo-1,3-diphenylprop-1-ene-2-sulfonamide (**7a**)

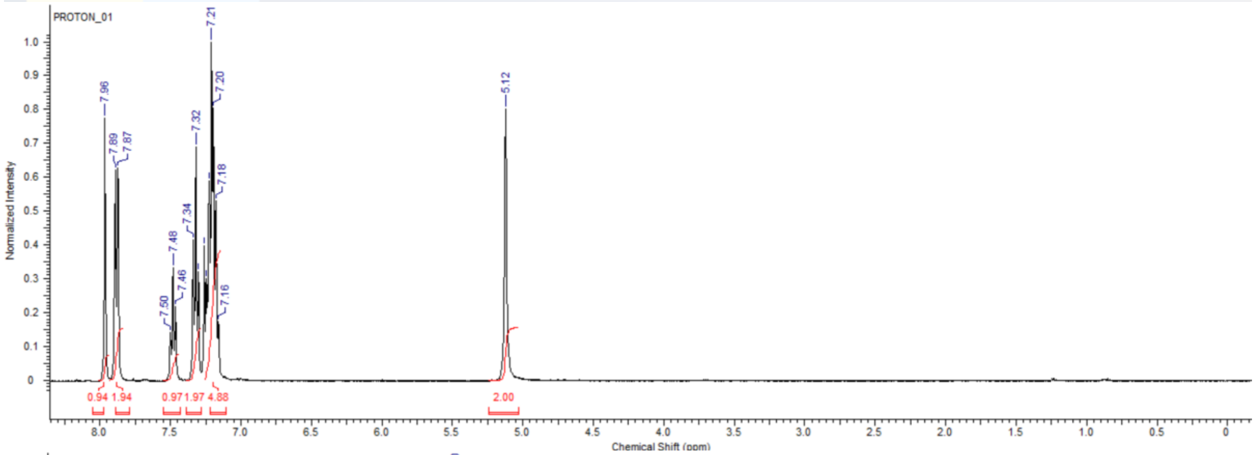

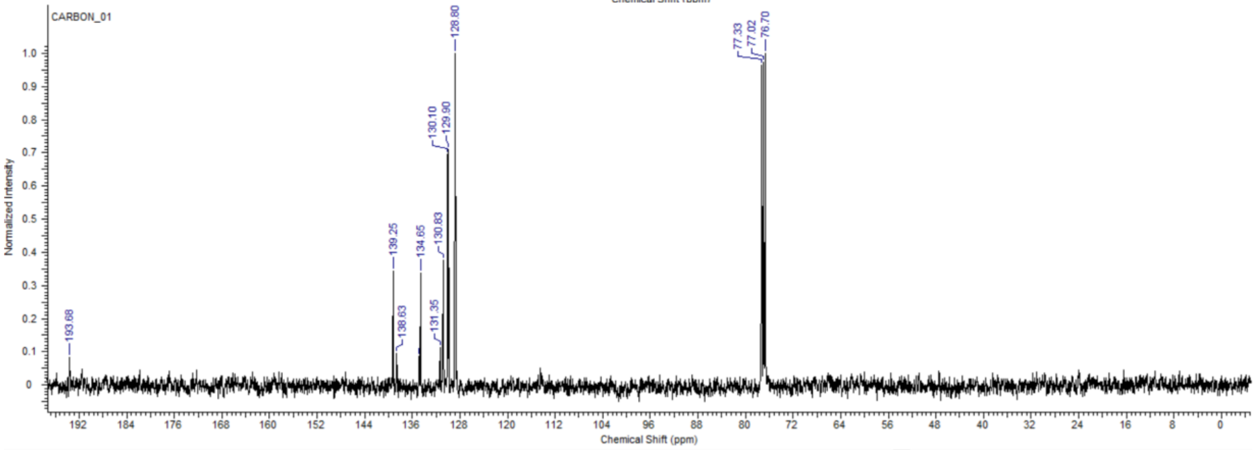

Chromatogram
ST-242 C:\LabSolutions\Data\CHEK\ST S0815-02.lcd

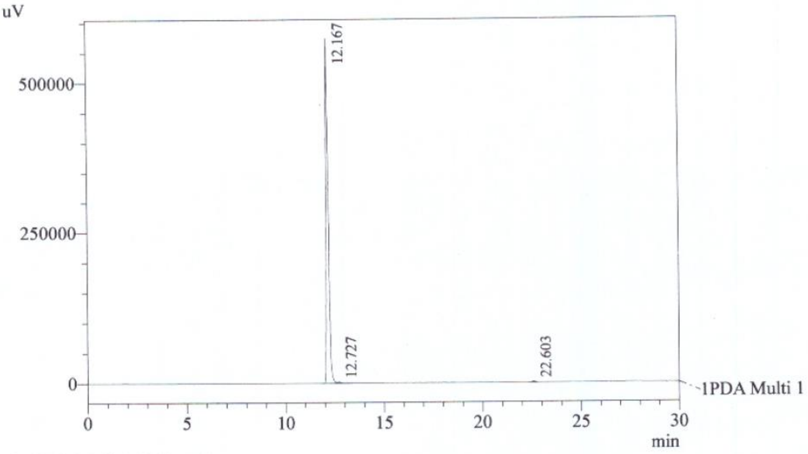

1 PDA Multi 1 / 260nm 4nm

PDA Ch1 260nm 4nm

Peak#	Ret. Time	Area	Height	Area %
1	12.167	4937769	572939	99.332
2	12.727	21106	1981	0.425
3	22.603	12086	1517	0.243
Total		4970960	576437	100.000

Method Filename : FOS B.lcm 15.08.2022 12:10:41

Time	Unit	Command	Valu
0.01	Pumps	B.Conc	40
30.00	Pumps	B.Conc	90
33.00	Pumps	B.Conc	40
45.00	Controller	Stop	

Shimadzu LC-20 AD; System - FOS Colon- Kromasil-100-5mkm. C-18, 4,6x250 mm. N 62511
Elution: A - H3PO4 0,01M pH 2,6; B - MeCN, fl - 1.0 ml/min, loop 20 mkl

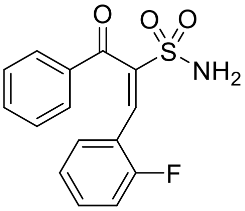

(E)-1-(2-fluorophenyl)-3-oxo-3-phenylprop-1-ene-2-sulfonamide (**7b**)

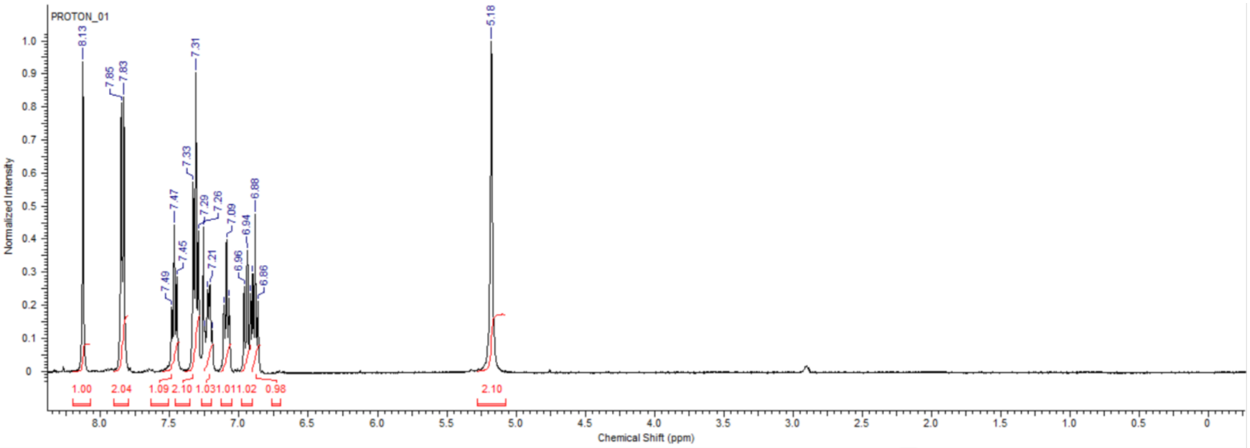

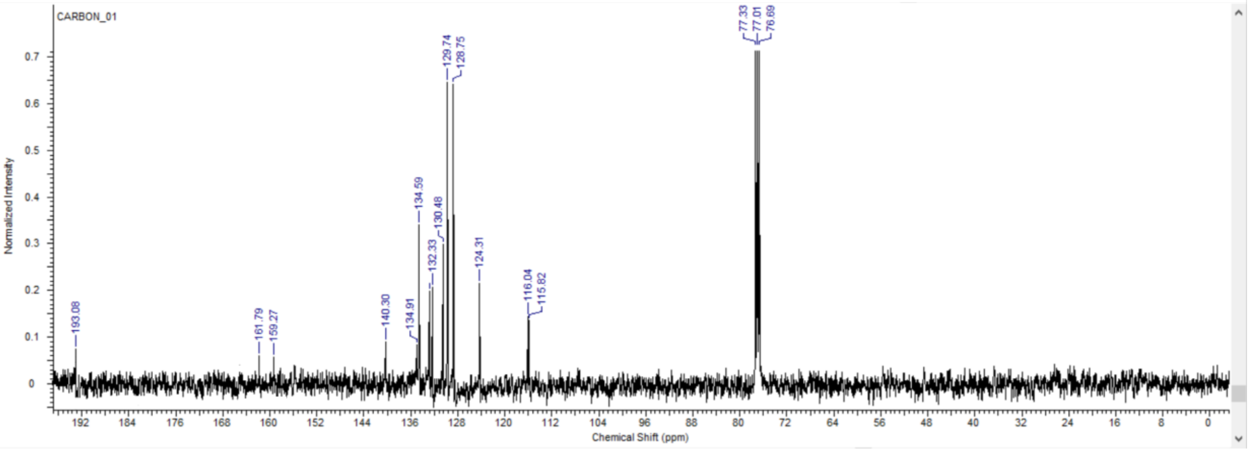

Chromatogram
ST-245 C:\LabSolutions\Data\CHEK\ST S0823-02.lcd

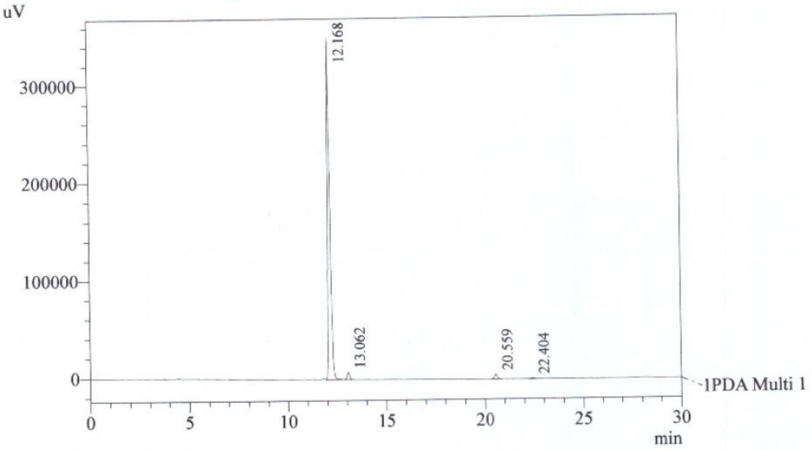

1 PDA Multi 1 / 260nm 4nm

PDA Ch1 260nm 4nm

Peak#	Ret. Time	Area	Height	Area %
1	12.168	3230131	351062	96.099
2	13.062	68951	7340	2.051
3	20.559	51609	5055	1.535
4	22.404	10563	946	0.314
Total		3361254	364403	100.000

Method Filename : FOS B.lcm 23.08.2022 11:24:28

Time	Unit	Command	Valu
0.01	Pumps	B.Conc	40
30.00	Pumps	B.Conc	90
33.00	Pumps	B.Conc	40
45.00	Controller	Stop	

Shimadzu LC-20 AD; System - FOS Colon- Kromasil-100-5mkm. C-18, 4,6x250 mm. N 62511
Elution: A - H3PO4 0,01M pH 2,6; B - MeCN, fl - 1.0 ml/min, loop 20 mkl

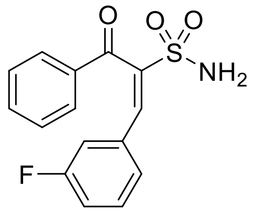

(E)-1-(3-fluorophenyl)-3-oxo-3-phenylprop-1-ene-2-sulfonamide (**7c**)

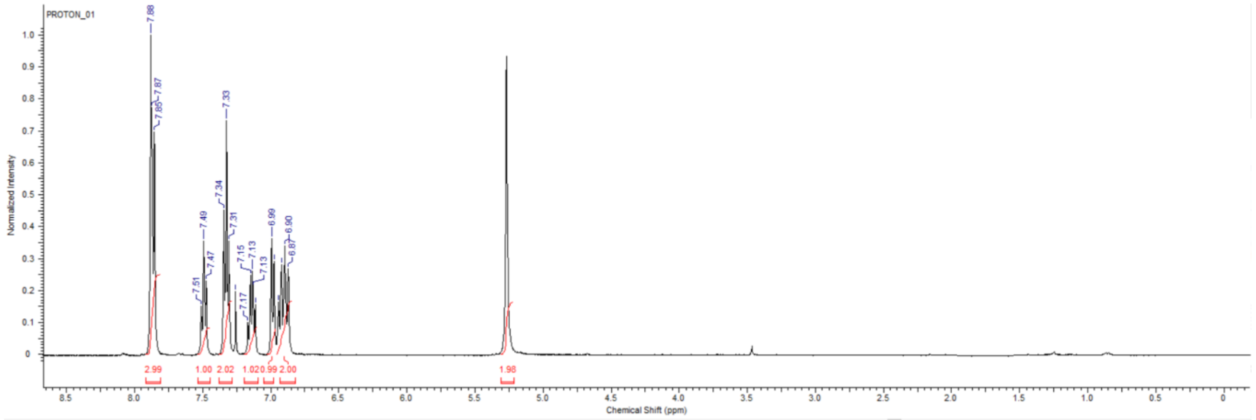

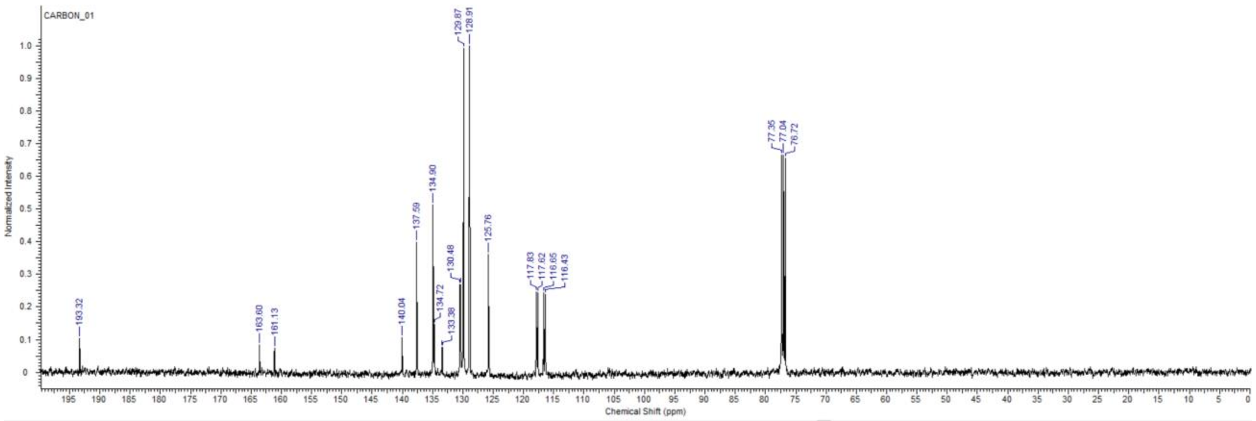

Chromatogram
ST-248 C:\LabSolutions\Data\CHEK\ST S0901-01.lcd

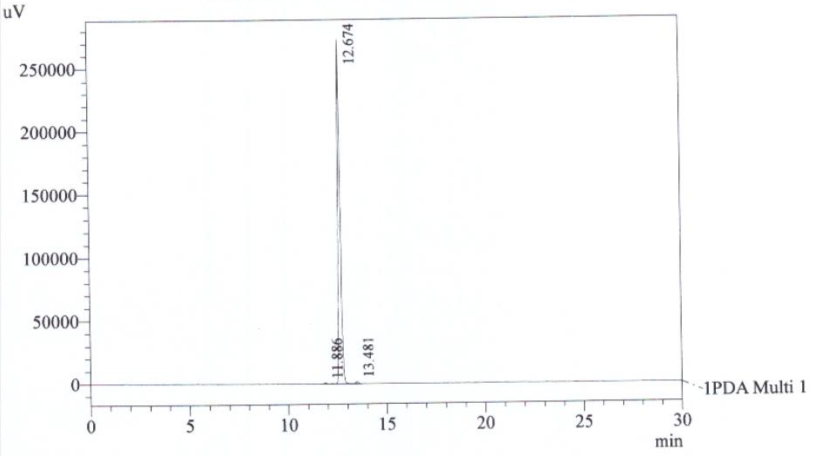

1 PDA Multi 1 / 260nm 4nm

PDA Ch1 260nm 4nm

Peak#	Ret. Time	Area	Height	Area %
1	11.886	10774	877	0.441
2	12.674	2415255	273682	98.970
3	13.481	14374	1610	0.589
Total		2440404	276169	100.000

Method Filename : FOS B.lcm 01.09.2022 13:17:26

Time	Unit	Command	Valu
0.01	Pumps	B.Conc	40
30.00	Pumps	B.Conc	90
33.00	Pumps	B.Conc	40
45.00	Controller	Stop	

Shimadzu LC-20 AD; System - FOS Colon- Kromasil-100-5mkm. C-18, 4,6x250 mm. N 62511
Elution: A - H3PO4 0,01M pH 2,6; B - MeCN, fl - 1.0 ml/min, loop 20 mkl

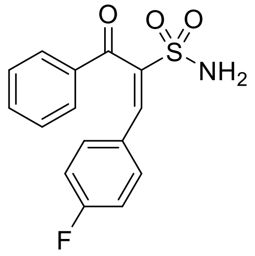

(*E*)-1-(4-fluorophenyl)-3-oxo-3-phenylprop-1-ene-2-sulfonamide (**7d**)

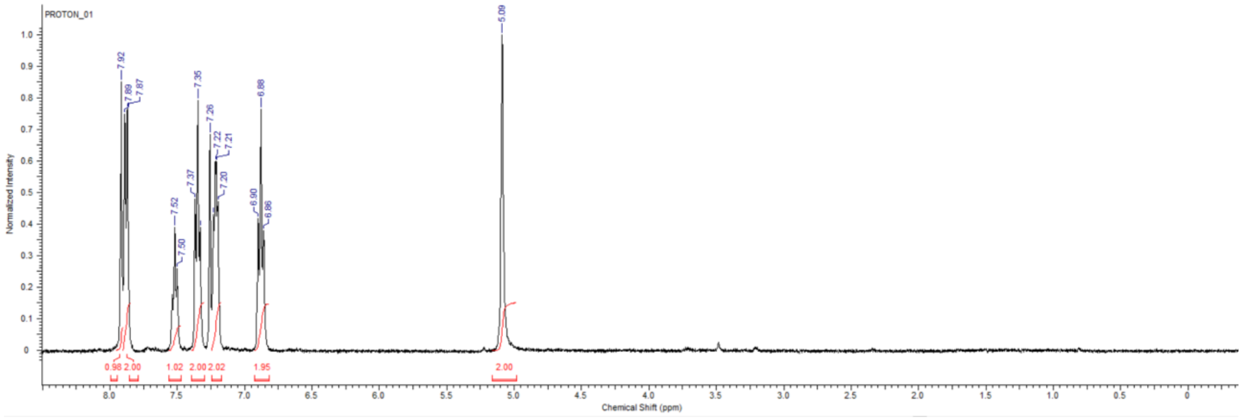

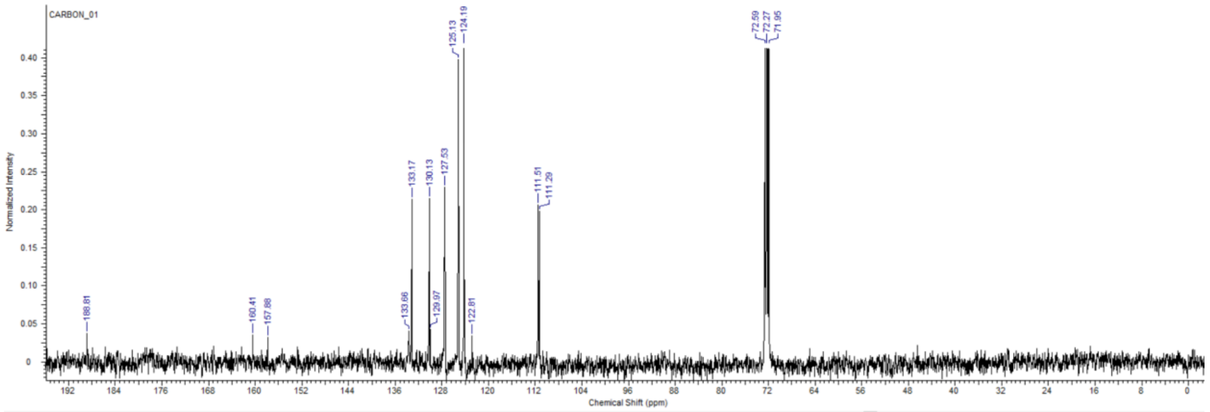

Chromatogram
ST-247 C:\LabSolutions\Data\CHEK\ST S0901-02.lcd

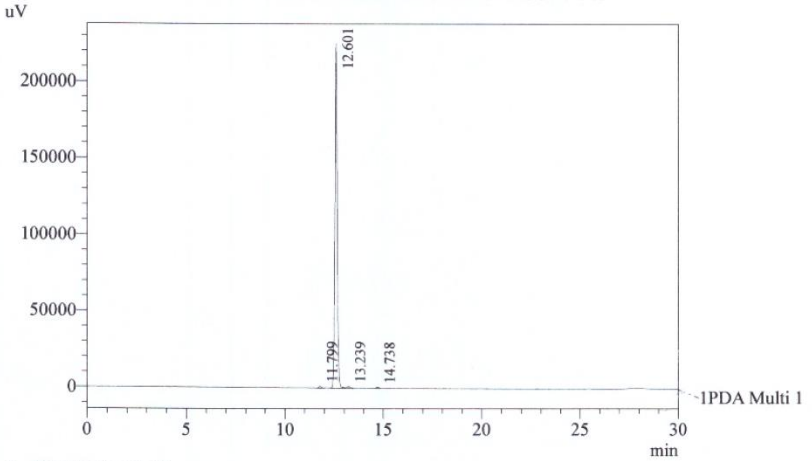

1 PDA Multi 1 / 260nm 4nm

PDA Ch1 260nm 4nm

Peak#	Ret. Time	Area	Height	Area %
1	11.799	11438	1248	0.570
2	12.601	1975737	225727	98.414
3	13.239	13287	1223	0.662
4	14.738	7108	707	0.354
Total		2007569	228905	100.000

Method Filename : FOS B.lcm 01.09.2022 14:18:22

Time	Unit	Command	Valu
0.01	Pumps	B.Conc	40
30.00	Pumps	B.Conc	90
33.00	Pumps	B.Conc	40
45.00	Controller	Stop	

Shimadzu LC-20 AD; System - FOS Colon- Kromasil-100-5mkm. C-18, 4,6x250 mm. N 62511
Elution: A - H_3PO_4 0,01M pH 2,6; B - MeCN, fl - 1.0 ml/min, loop 20 mkl

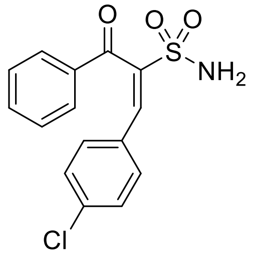

(*E*)-1-(4-chlorophenyl)-3-oxo-3-phenylprop-1-ene-2-sulfonamide (**7e**)

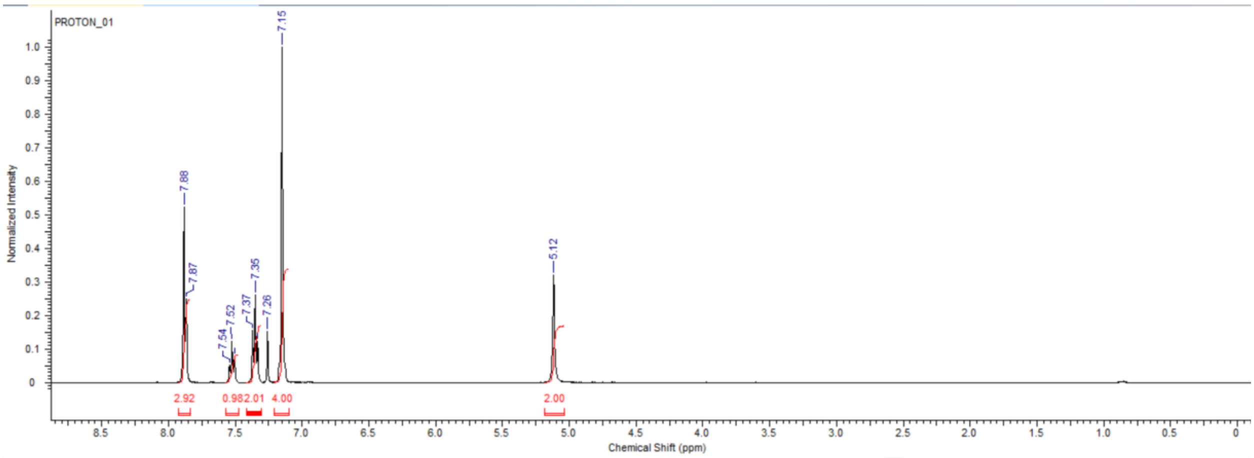

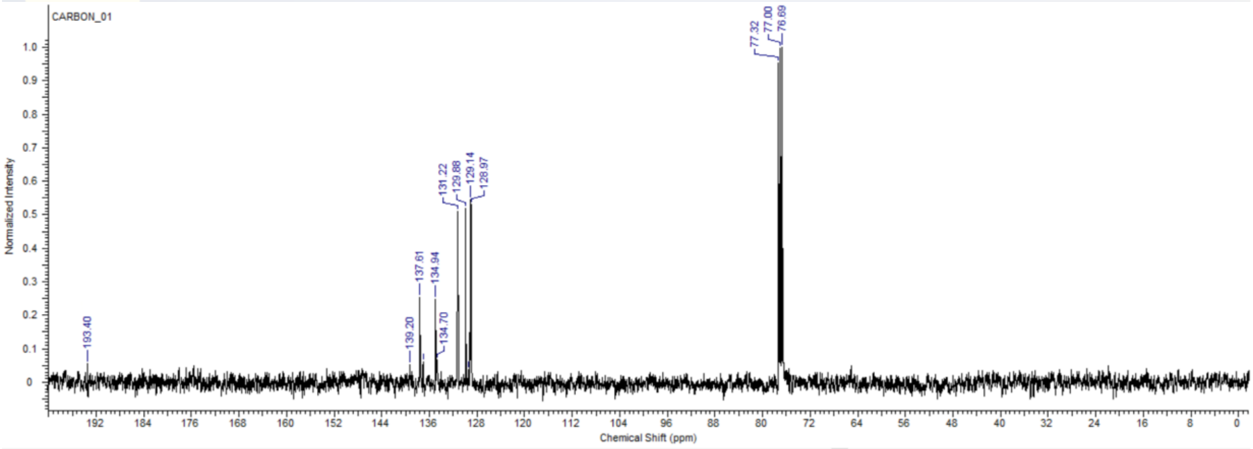

Chromatogram
ST-240 C:\LabSolutions\Data\CHEK\ST S0811-03.lcd

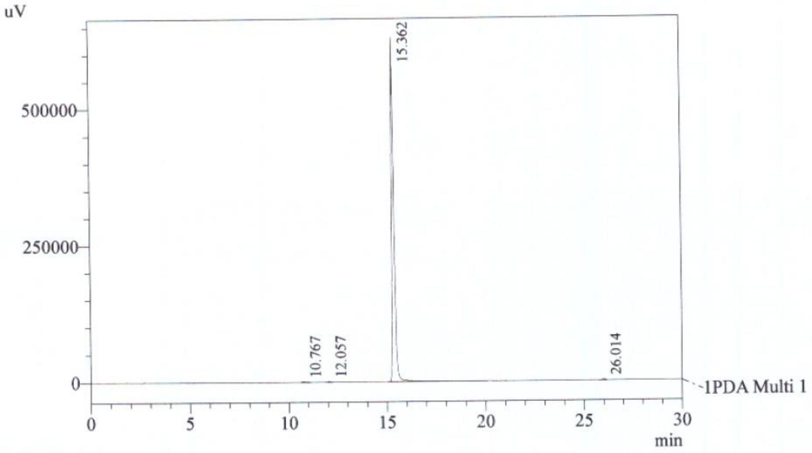

1 PDA Multi 1 / 260nm 4nm

PDA Ch1 260nm 4nm

Peak#	Ret. Time	Area	Height	Area %
1	10.767	12669	1248	0.219
2	12.057	11371	1547	0.196
3	15.362	5745646	630596	99.268
4	26.014	18320	2184	0.317
Total		5788005	635575	100.000

Method Filename : FOS B.lcm 11.08.2022 13:17:22

Time	Unit	Command	Valu
0.01	Pumps	B.Conc	40
30.00	Pumps	B.Conc	90
33.00	Pumps	B.Conc	40
45.00	Controller	Stop	

Shimadzu LC-20 AD; System - FOS Colon- Kromasil-100-5mkm. C-18, 4,6x250 mm. N 62511
Elution: A - H3PO4 0,01M pH 2,6; B - MeCN, fl - 1.0 ml/min, loop 20 mkl

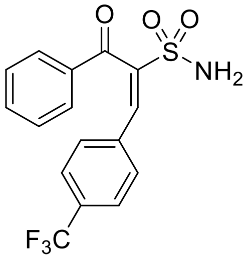

(*E*)-3-oxo-3-phenyl-1-(4-(trifluoromethyl)phenyl)prop-1-ene-2-sulfonamide (**7f**)

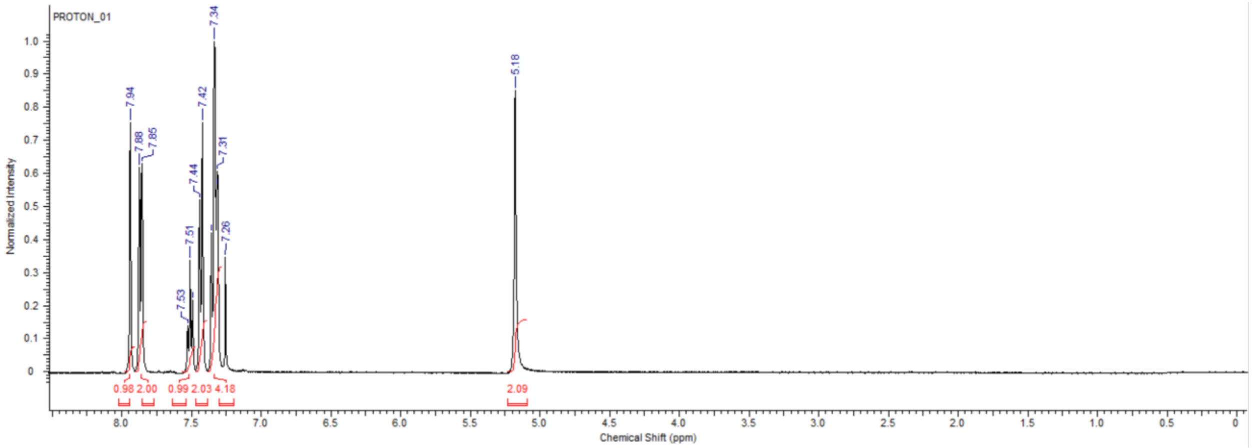

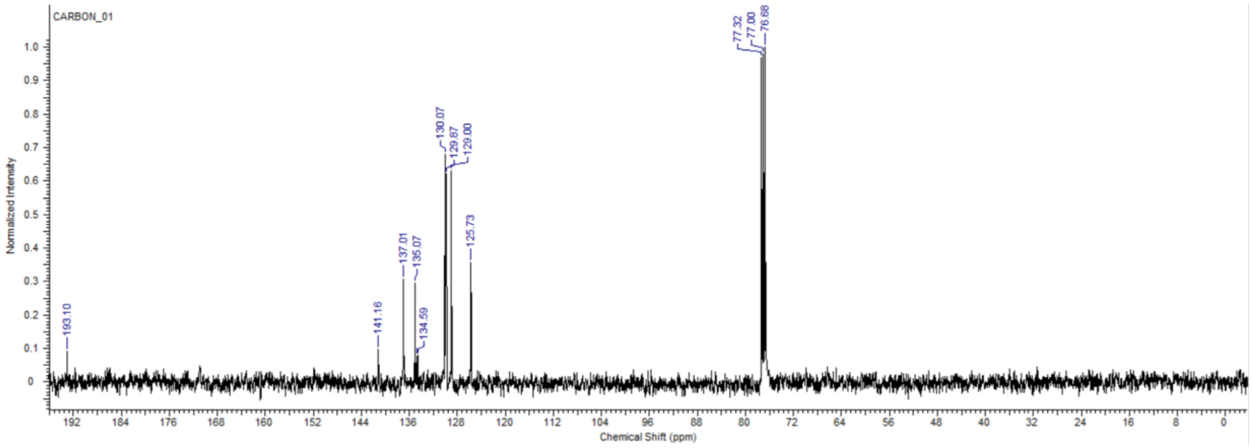

Chromatogram
ST-244 C:\LabSolutions\Data\CHEK\ST S0823-01.lcd

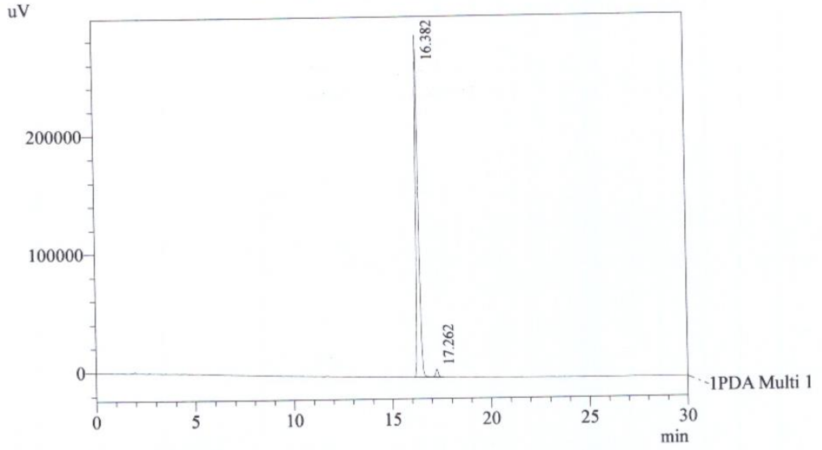

1 PDA Multi 1 / 260nm 4nm

PDA Ch1 260nm 4nm

Peak#	Ret. Time	Area	Height	Area %
1	16.382	2750488	288752	97.867
2	17.262	59948	6427	2.133
Total		2810436	295179	100.000

Method Filename : FOS B.lcm 23.08.2022 10:33:27

Time	Unit	Command	Valu
0.01	Pumps	B.Conc	40
30.00	Pumps	B.Conc	90
33.00	Pumps	B.Conc	40
45.00	Controller	Stop	

Shimadzu LC-20 AD; System - FOS Colon- Kromasil-100-5mkm. C-18, 4,6x250 mm. N 62511
Elution: A - H3PO4 0,01M pH 2,6; B - MeCN, fl - 1.0 ml/min, loop 20 mkl

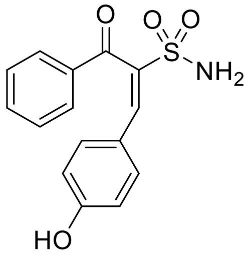

(*E*)-1-(4-hydroxyphenyl)-3-oxo-3-phenylprop-1-ene-2-sulfonamide (**7g**)

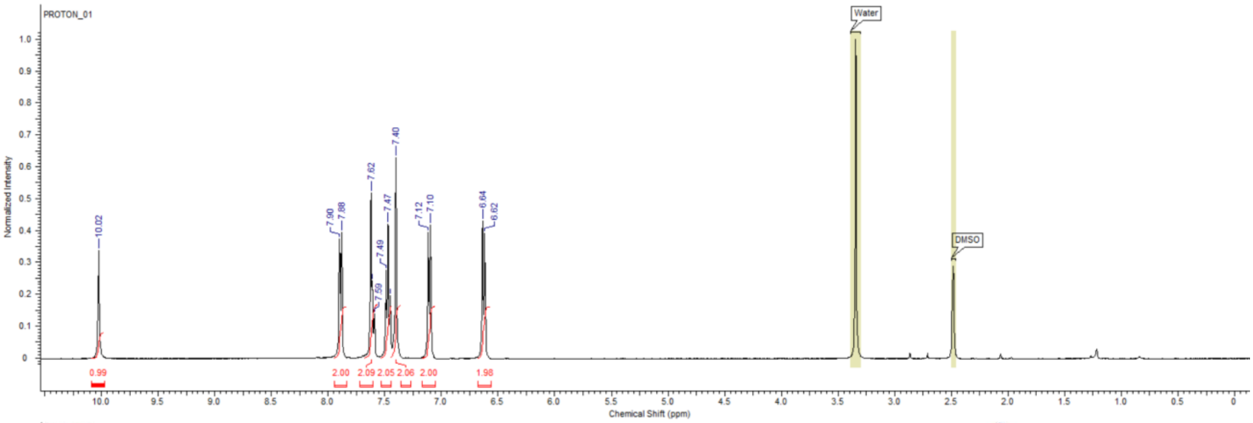

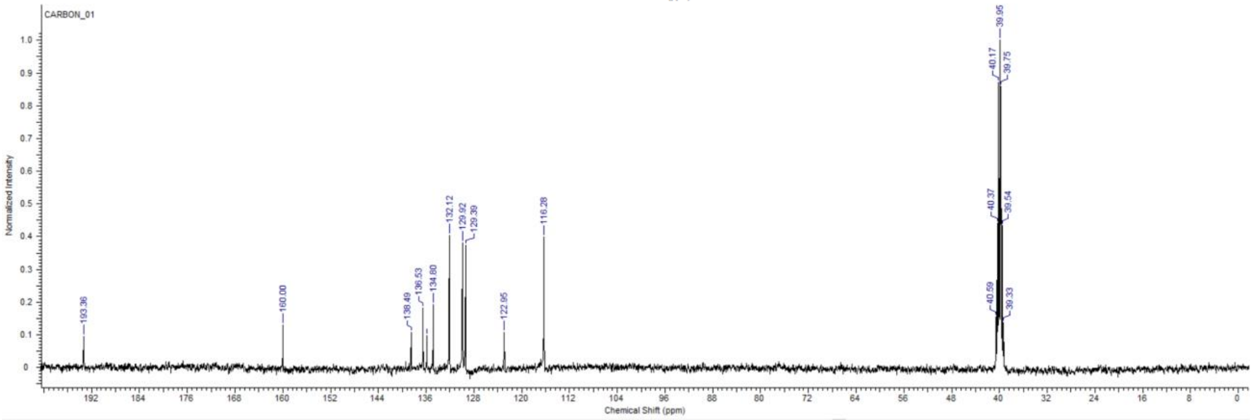

Chromatogram
ST-254 C:\LabSolutions\Data\CHEK\ST S1003-04.lcd

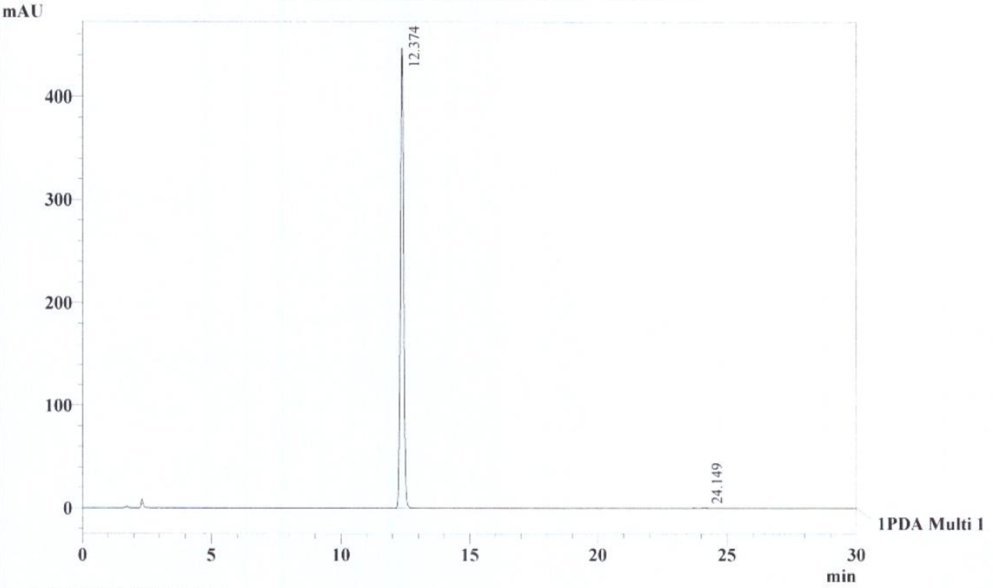

1 PDA Multi 1 / 254nm 4nm

PeakTable

PDA Ch1 254nm 4nm

Peak#	Ret. Time	Area	Height	Area %
1	12.374	3953006	447406	99.714
2	24.149	11328	779	0.286
Total		3964334	448185	100.000

Method

<<LC Program>>

Time	Unit	Command	Value
0.10	Pumps	B.Conc	30
30.00	Pumps	B.Conc	70
33.00	Pumps	B.Conc	30
45.00	Controller	Stop	

Method Filename : FOS Cv.lcm

Shimadzu LC-20AD; 2-System FOS, Colon Kromasil 100-C18,.size 5mkm, 4,6*250mm, N 86912
Elution: A - H3PO4 0.01M pH 2.6; B - MeCN, fl. 1,0 ml/min, loop 20mkl.

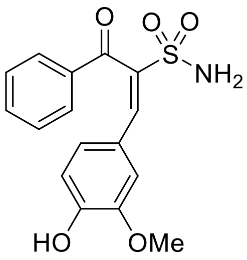

(*E*)-1-(4-hydroxy-3-methoxyphenyl)-3-oxo-3-phenylprop-1-ene-2-sulfonamide (**7h**)

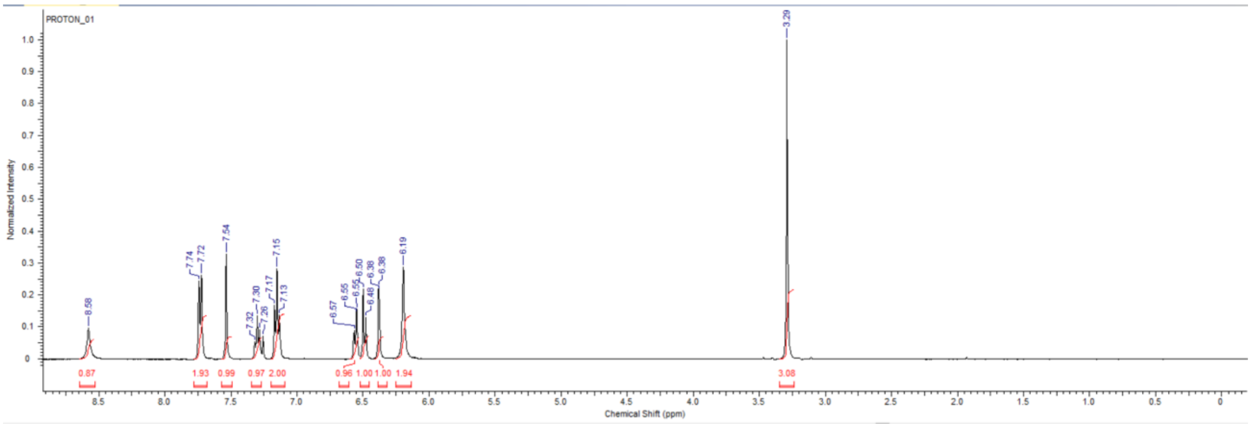

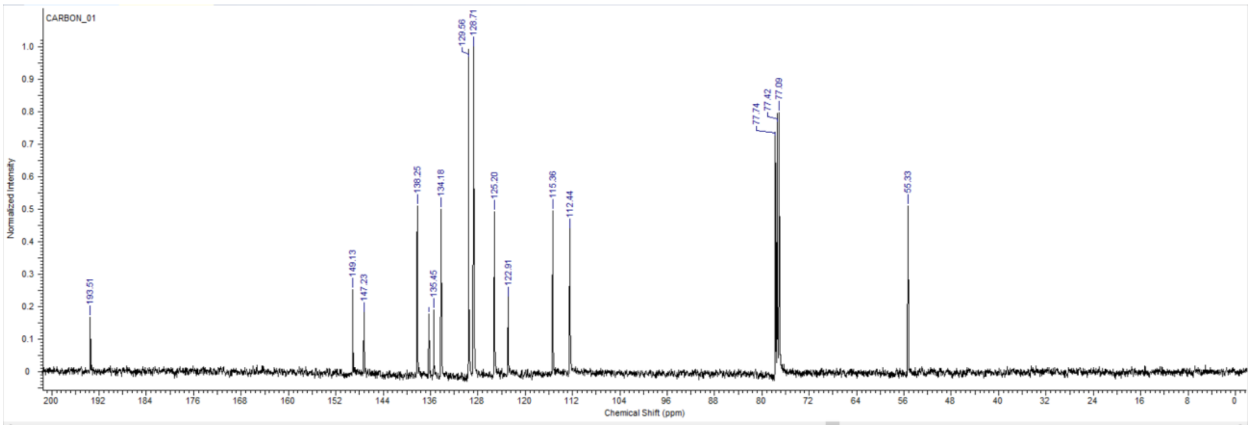

Chromatogram
ST-262 C:\LabSolutions\Data\CHEK\ST S1110-02.lcd

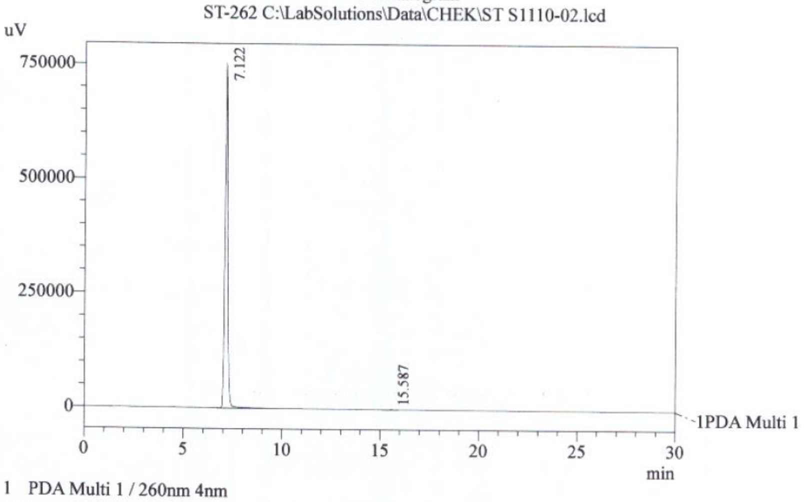

1 PDA Multi 1 / 260nm 4nm

PDA Ch1 260nm 4nm

Peak#	Ret. Time	Area	Height	Area %
1	7.122	7702425	754910	99.891
2	15.587	8436	562	0.109
Total		7710860	755473	100.000

Method Filename : FOS B.lcm 10.11.2022 12:17:34

Time	Unit	Command	Valu
0.01	Pumps	B.Conc	40
30.00	Pumps	B.Conc	90
33.00	Pumps	B.Conc	40
45.00	Controller	Stop	

Shimadzu LC-20 AD; System - FOS Colon- Kromasil-100-5mkm. C-18, 4,6x250 mm. N 62511
Elution: A - H3PO4 0,01M pH 2,6; B - MeCN, fl - 1.0 ml/min, loop 20 mkl

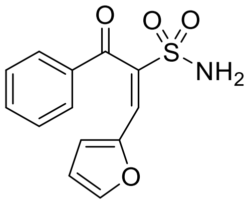

(E)-1-(furan-2-yl)-3-oxo-3-phenylprop-1-ene-2-sulfonamide (**7i**)

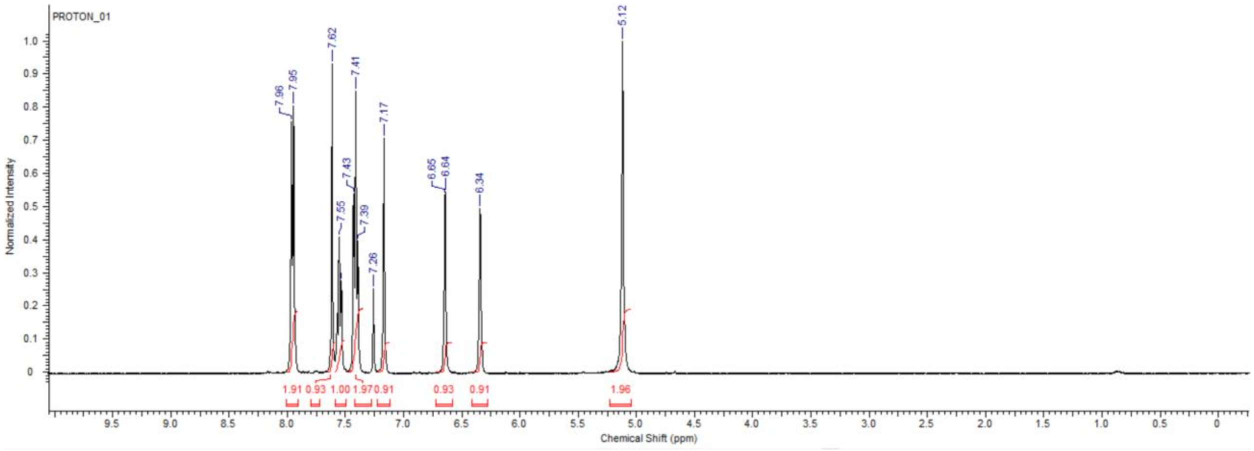

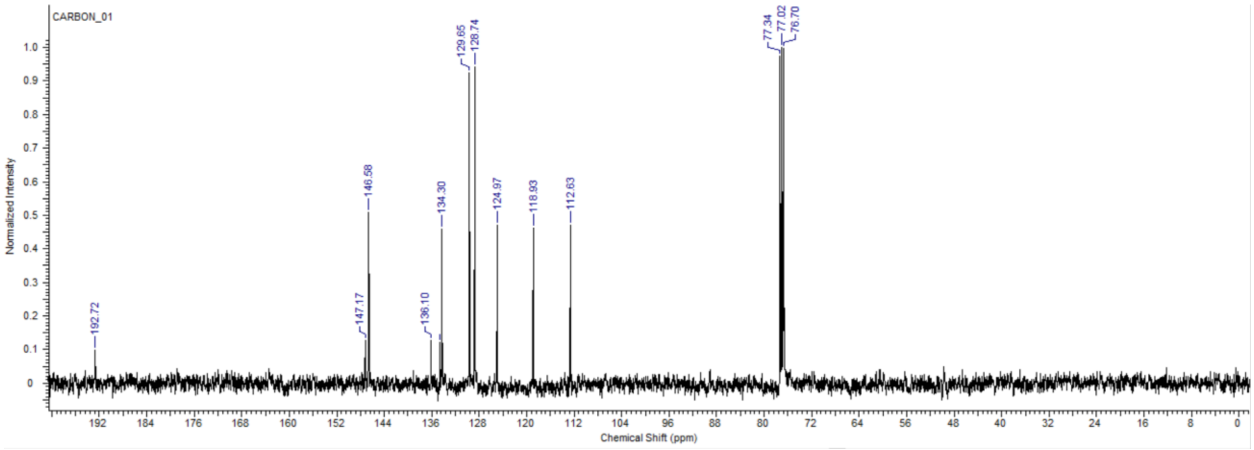

Chromatogram
ST-252 C:\LabSolutions\Data\CHEK\ST 0916-02.lcd

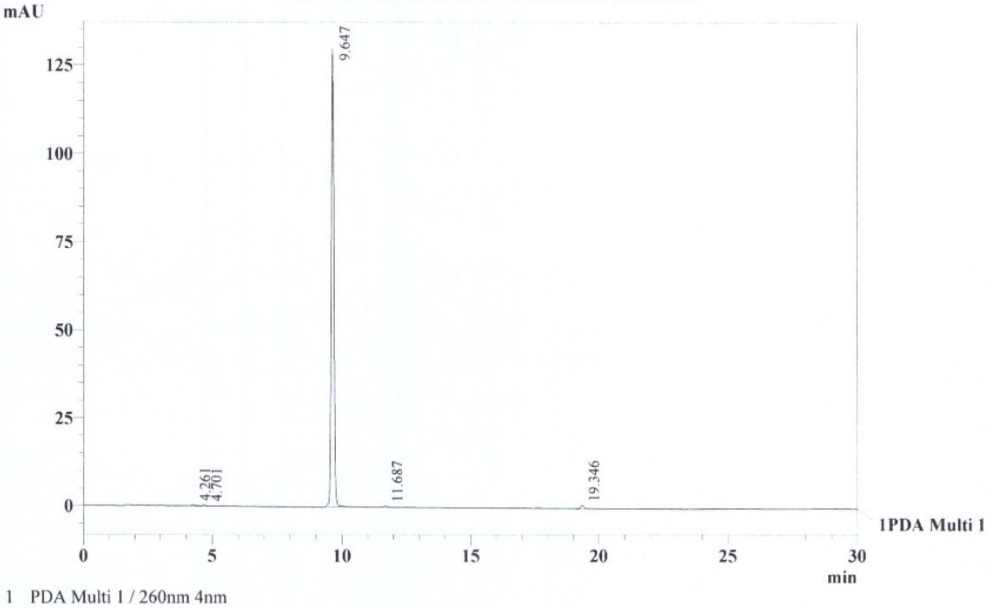

1 PDA Multi 1 / 260nm 4nm

PeakTable

PDA Ch1 260nm 4nm

Peak#	Ret. Time	Area	Height	Area %
1	4.261	2580	333	0.278
2	4.701	1013	175	0.109
3	9.647	915660	130177	98.490
4	11.687	2475	358	0.266
5	19.346	7969	989	0.857
Total		929697	132032	100.000

Method

<<LC Program>>

Time	Unit	Command	Value
0.10	Pumps	B.Conc	40
30.00	Pumps	B.Conc	90
33.00	Pumps	B.Conc	40
45.00	Controller	Stop	

Method Filename : FOS C.lcm

Shimadzu LC-20AD; 2-System FOS, Colon Kromasil 100-C18,.size 5mkm, 4,6*250mm, N 86912
Elution: A - H3PO4 0.01M pH 2.6; B - MeCN, fl. 1,0 ml/min, loop 20mkl.

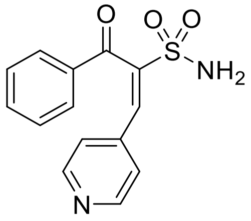

(*E*)-3-oxo-3-phenyl-1-(pyridin-4-yl)prop-1-ene-2-sulfonamide (**7j**)

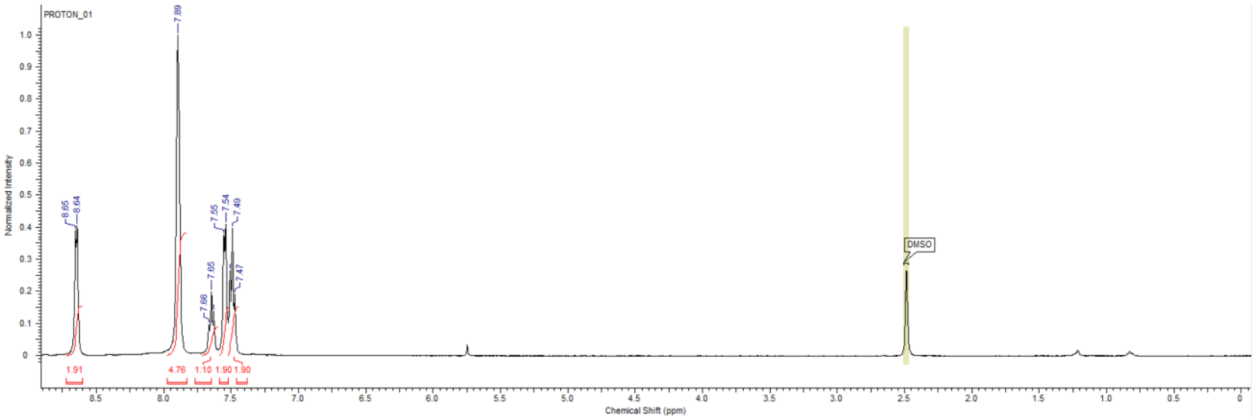

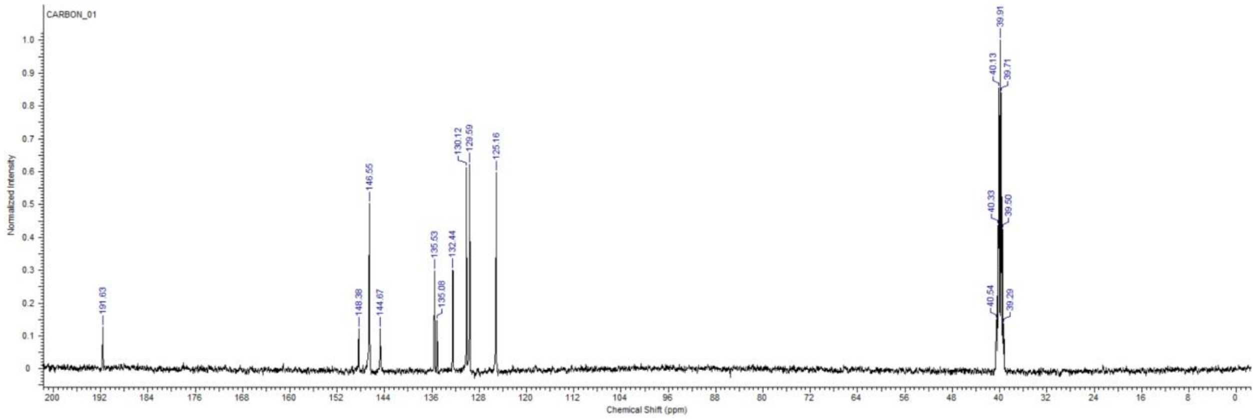

Chromatogram
ST-241 C:\LabSolutions\Data\CHEK\ST S0815-03.lcd

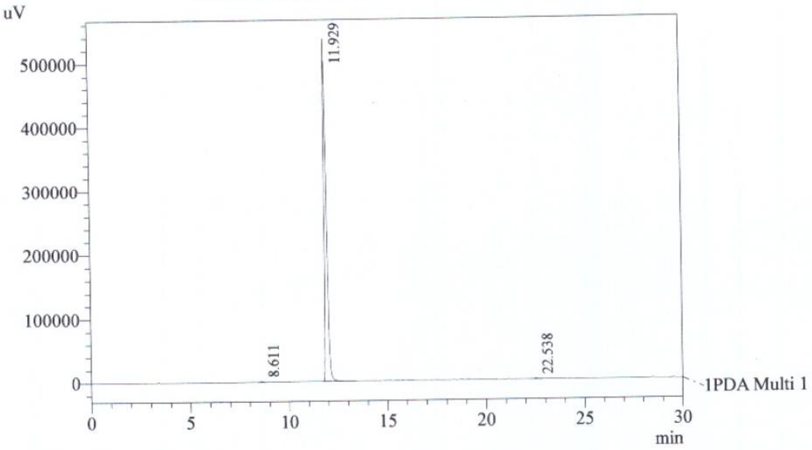

1 PDA Multi 1 / 260nm 4nm

PDA Ch1 260nm 4nm

Peak#	Ret. Time	Area	Height	Area %
1	8.611	13630	1318	0.288
2	11.929	4705042	535625	99.465
3	22.538	11680	1272	0.247
Total		4730353	538214	100.000

Method Filename : FOS B.lcm 15.08.2022 13:32:25

Time	Unit	Command	Valu
0.01	Pumps	B.Conc	10
30.00	Pumps	B.Conc	60
33.00	Pumps	B.Conc	10
45.00	Controller	Stop	

Shimadzu LC-20 AD; System - FOS Colon- Kromasil-100-5mkm. C-18, 4,6x250 mm. N 62511
Elution: A - H3PO4 0,01M pH 2,6; B - MeCN, fl - 1.0 ml/min, loop 20 mkl

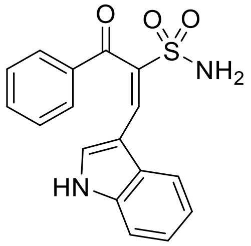

(*E*)-1-(1*H*-indol-3-yl)-3-oxo-3-phenylprop-1-ene-2-sulfonamide (**7k**)

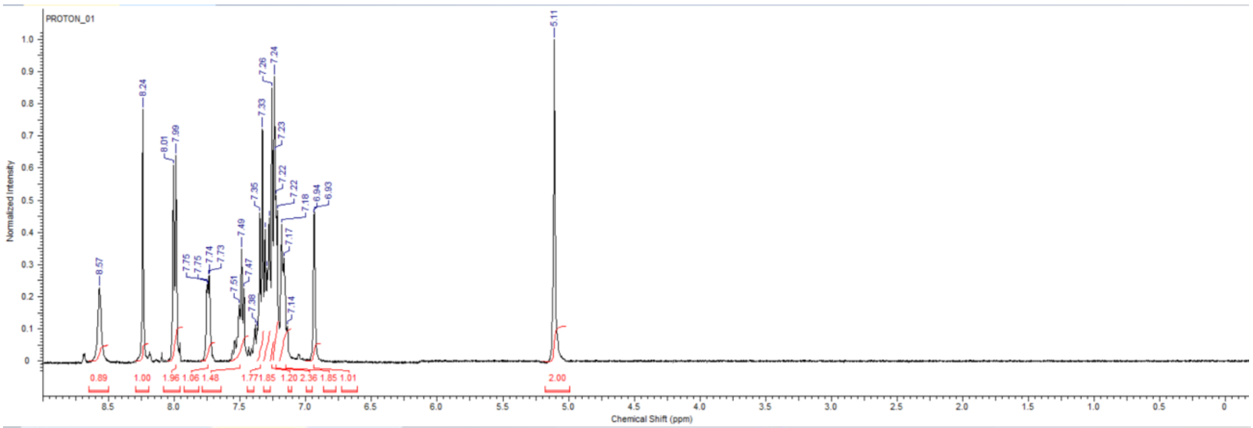

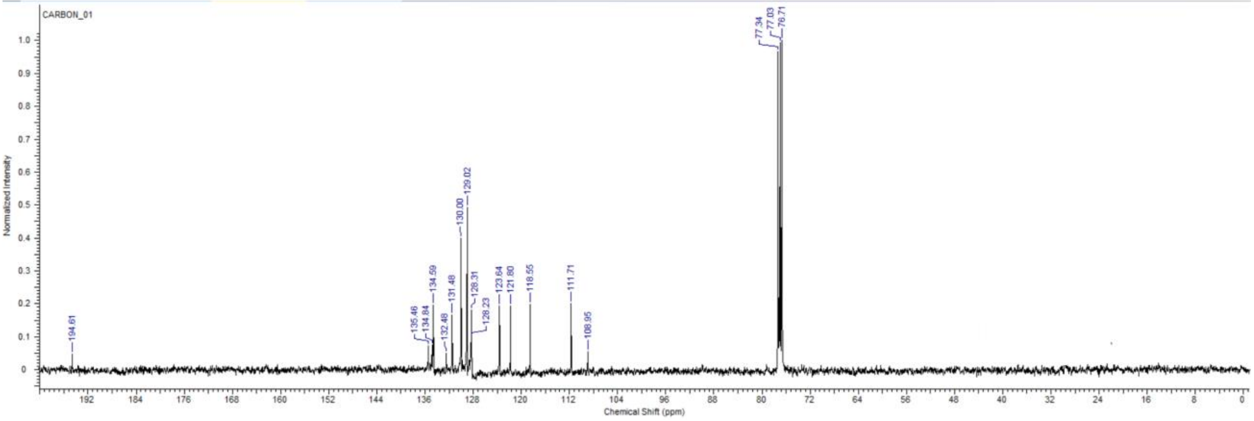

Chromatogram
ST-237 C:\LabSolutions\Data\CHEK\ST S0811-05.lcd

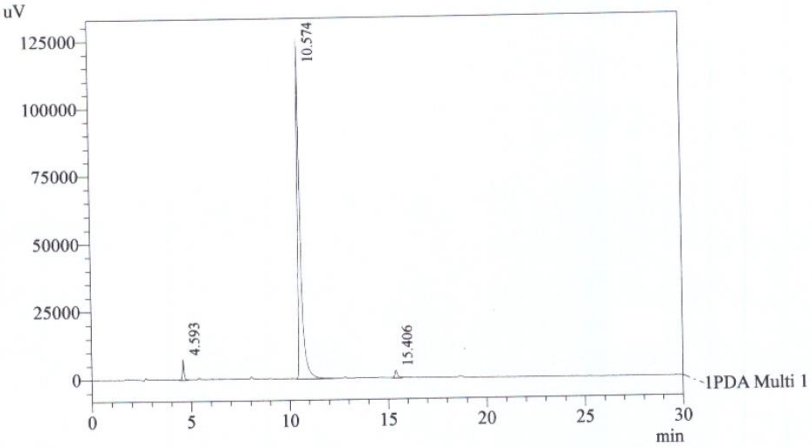

1 PDA Multi 1 / 260nm 4nm

PDA Ch1 260nm 4nm

Peak#	Ret. Time	Area	Height	Area %
1	4.593	48833	7620	3.488
2	10.574	1265539	125908	94.566
3	15.406	27238	2757	1.945
Total		1400115	140131	100.000

Method Filename : FOS B.lcm 11.08.2022 14:52:42

Time	Unit	Command	Valu
0.01	Pumps	B.Conc	40
30.00	Pumps	B.Conc	90
33.00	Pumps	B.Conc	40
45.00	Controller	Stop	

Shimadzu LC-20 AD; System - FOS Colon- Kromasil-100-5mkm. C-18, 4,6x250 mm. N 62511
Elution: A - H3PO4 0,01M pH 2,6; B - MeCN, fl - 1.0 ml/min, loop 20 mkl

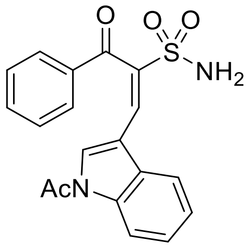

(F)-1-(1-acetyl-1*H*-indol-3-yl)-3-oxo-3-phenylprop-1-ene-2-sulfonamide (**7l**)

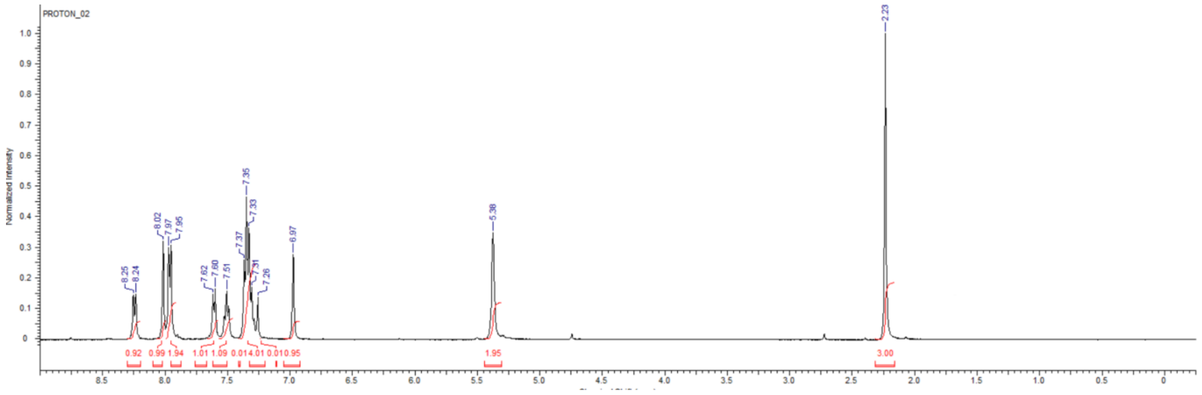

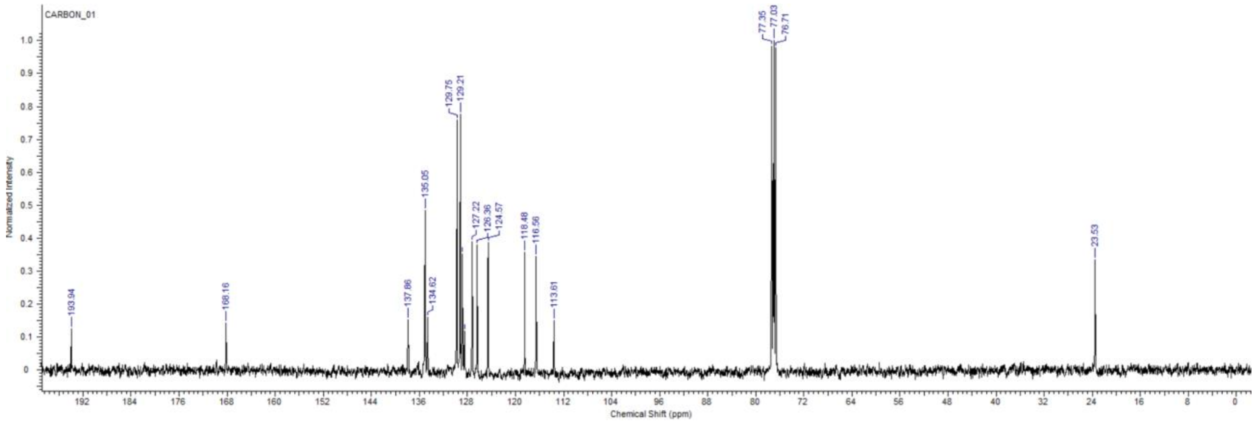

Chromatogram
ST-235 C:\LabSolutions\Data\CHEK\ST S0811-04.lcd

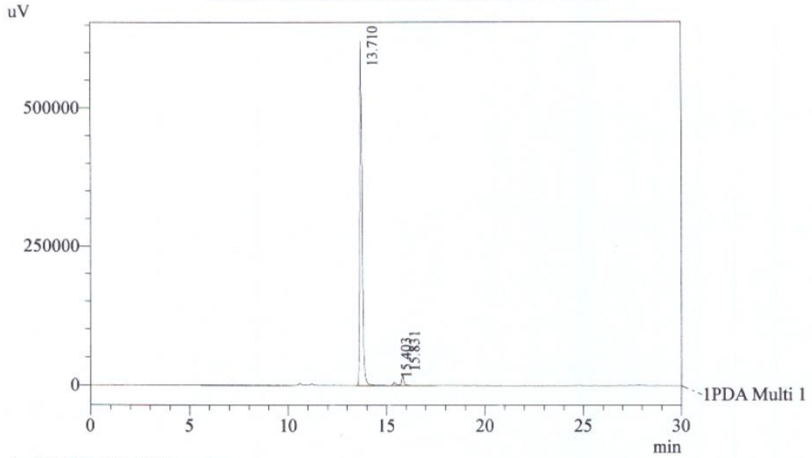

1 PDA Multi 1 / 254nm 4nm

PDA Ch1 254nm 4nm

Peak#	Ret. Time	Area	Height	Area %
1	13.710	5650631	620863	96.285
2	15.403	61564	5805	1.049
3	15.831	156434	18107	2.666
Total		5868629	656912	100.000

Method Filename : FOS B.lcm 11.08.2022 14:04:32

Time	Unit	Command	Valu
0.01	Pumps	B.Conc	40
30.00	Pumps	B.Conc	90
33.00	Pumps	B.Conc	40
45.00	Controller	Stop	

Shimadzu LC-20 AD; System - FOS Colon- Kromasil-100-5mkm. C-18, 4,6x250 mm. N 62511
Elution: A - H3PO4 0,01M pH 2,6; B - MeCN, fl - 1.0 ml/min, loop 20 mkl

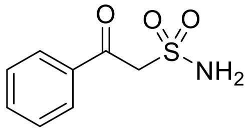

2-oxo-2-phenylethane-1-sulfonamide (**11**)

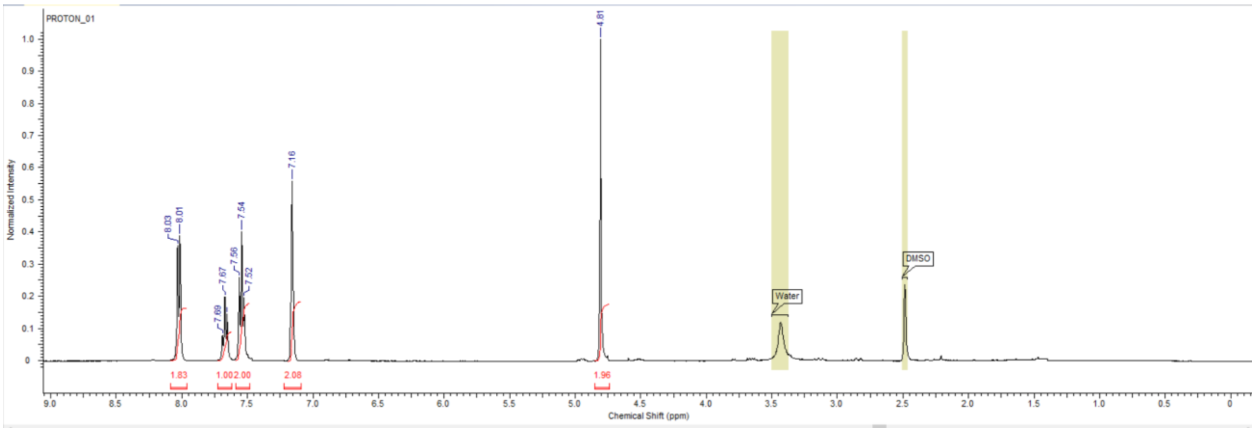

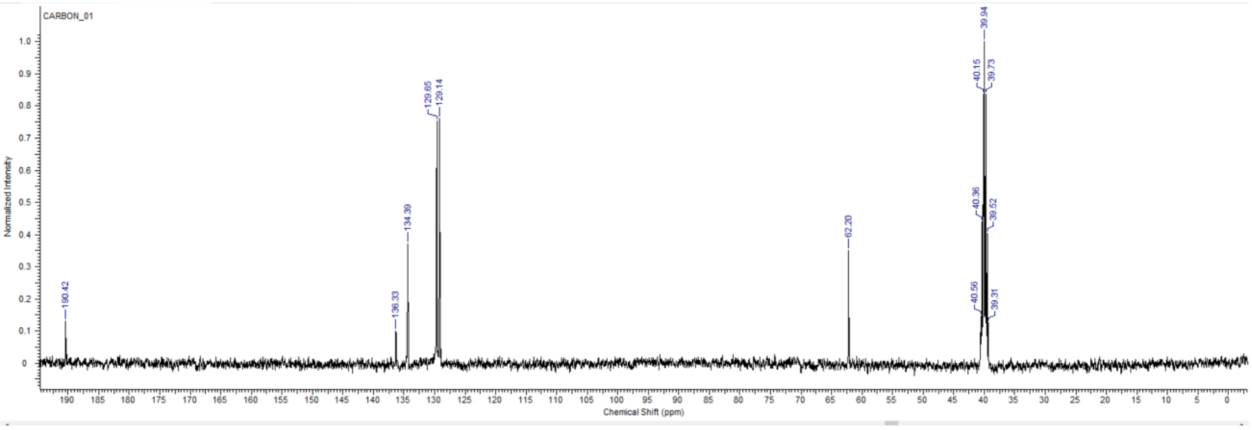

Chromatogram
ST-255 C:\LabSolutions\Data\CHEK\ST S1110-03.lcd

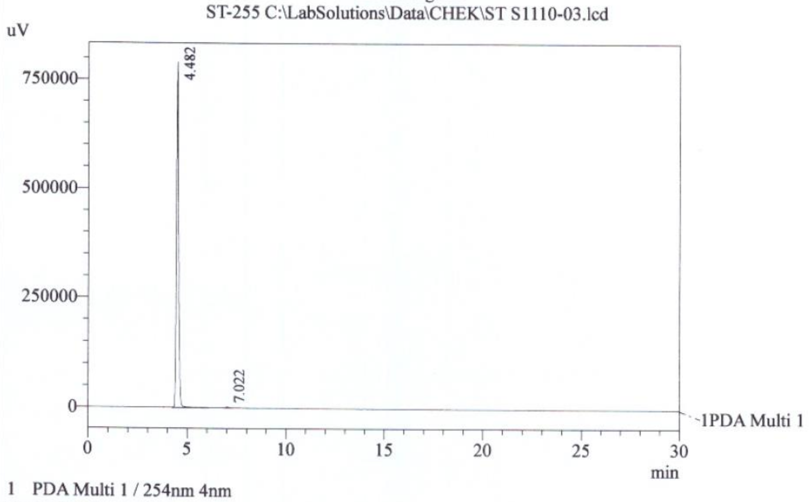

1 PDA Multi 1 / 254nm 4nm

PDA Ch1 254nm 4nm

Peak#	Ret. Time	Area	Height	Area %
1	4.482	6468293	790739	99.604
2	7.022	25725	2679	0.396
Total		6494018	793417	100.000

Method Filename : FOS B.lcm 10.11.2022 12:59:59

Time	Unit	Command	Valu
0.01	Pumps	B.Conc	40
30.00	Pumps	B.Conc	90
33.00	Pumps	B.Conc	40
45.00	Controller	Stop	

Shimadzu LC-20 AD; System - FOS Colon- Kromasil-100-5mkm. C-18, 4,6x250 mm. N 62511
Elution: A - H3PO4 0,01M pH 2,6; B - MeCN, fl - 1.0 ml/min, loop 20 mkl

Table S1. Crystallographic data for **7a**

Formula moiety	$C_{15}H_{13}NO_3S$
Brutto formula	$C_{15}H_{13}NO_3S$
Formula weight	287.32
Diffractometer	Bruker QUEST
Scan mode	ω and ϕ scans
Anode [Wavelength, Å]	MoKα [0.71073] microfocus sealed X-ray tube
Crystal Dimensions, mm	0.07 × 0.31 × 0.31
Crystal color	colourless
Crystal system	monoclinic
a, Å	10.3899(3)
b, Å	33.2046(9)
c, Å	16.2988(4)
α, °	90
β, °	102.374(2)
γ, °	90
Volume, Å^3	5492.3(3)
Density, gcm^{-3}	1.390
Temperature, K	100
T_{min}/T_{max}	0.4273/0.4998
μ, mm^{-1}	0.242
Space group	Cc
Z	16
F(000)	2400

Reflections collected	39106
Independent reflections	14133
Reflections (I>2σ(I))	12646
Parameters	729
R_{int}	0.0419
$2\theta_{min}$ - $2\theta_{max}$, °	4.196 - 61.016
wR_2 (all reflections)	0.1181
R_1(I>σ(I))	0.0485
GOF	1.060
$\varrho_{min}/\varrho_{max}$, $eÅ^{-3}$	-0.404/0.948
Restraints	2

Hydrogen bonding in **7a**

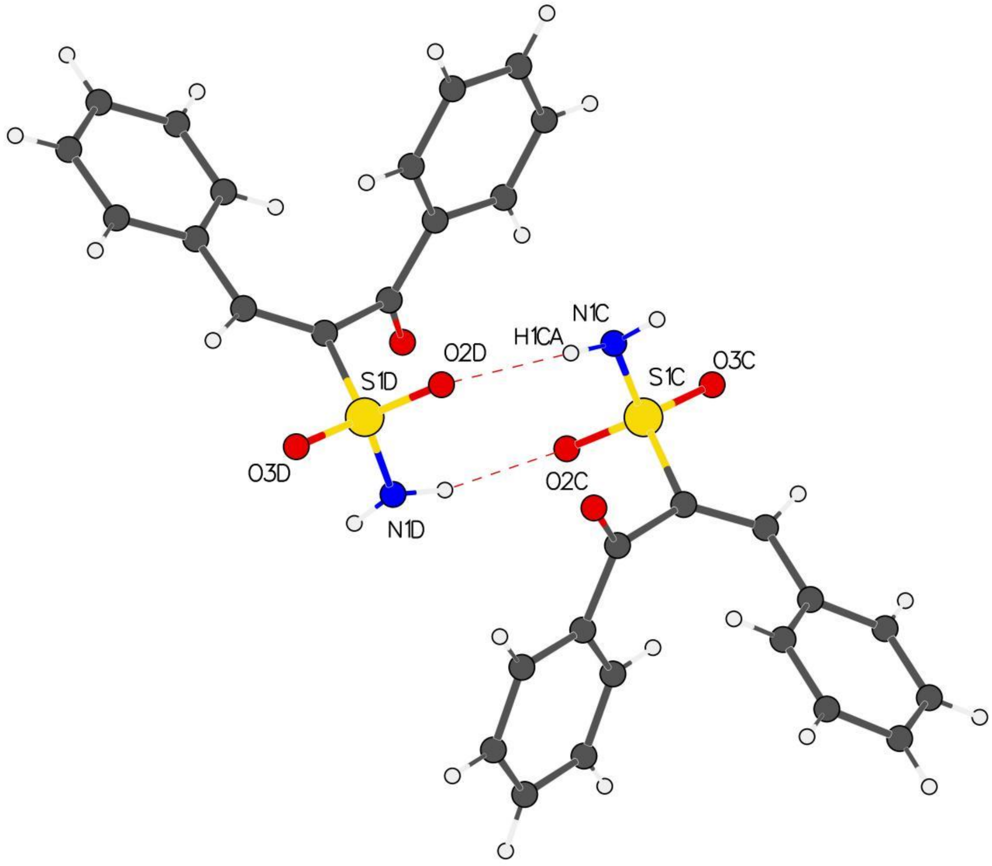

Figure S1. Centrosymmetric dimer in crystal structure of **7a** formed by hydrogen bonds between sufonamide groups.

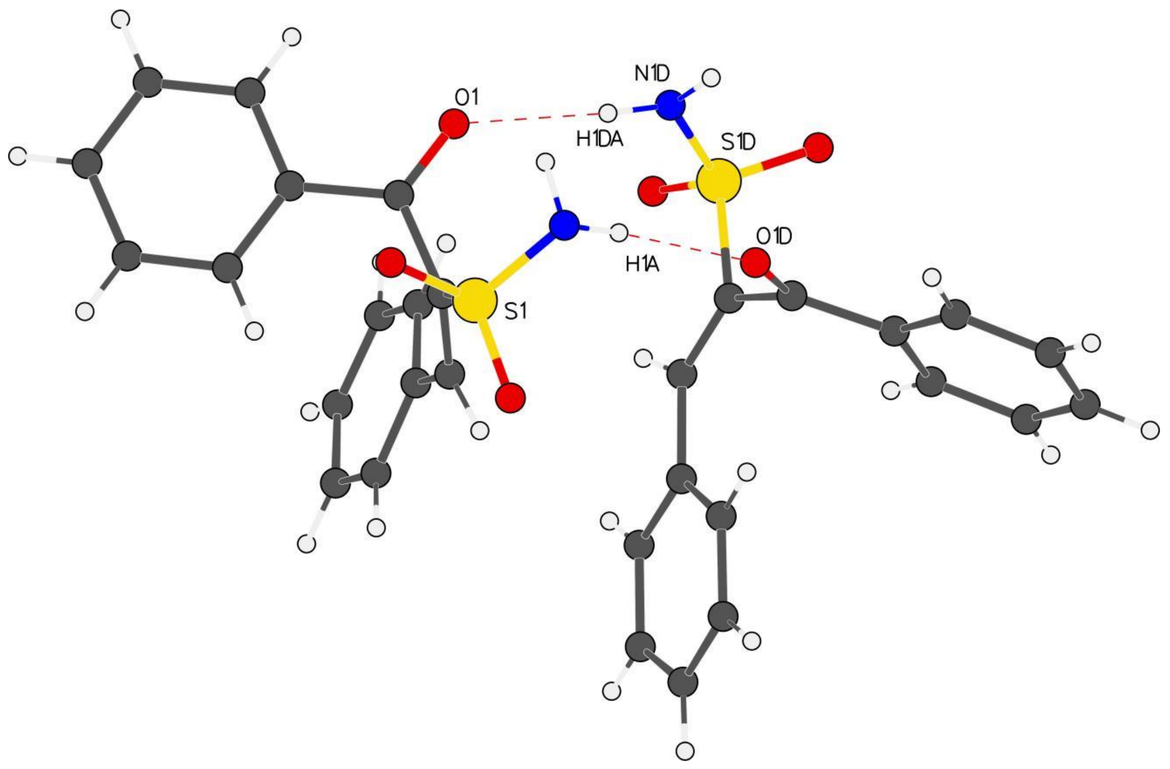

Figure S2. Centrosymmetric dimer in crystal structure of **7a** formed by hydrogen bonds between sufonamide and carbonyl groups.

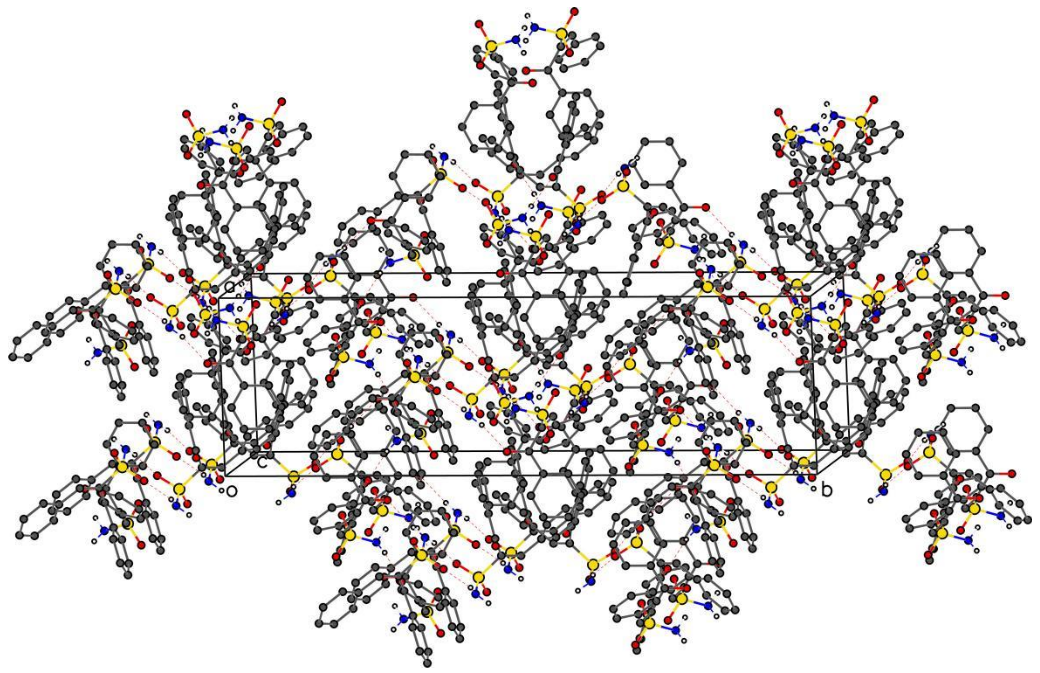

Figure S3. Crystal packing of **7a**.

D	H	A	d(D-H)/Å	d(H-A)/Å	d(D-A)/Å	D-H-A/°
N1	H1A	O1D[1]	0.90	1.99	2.864(4)	163.4
N1A	H1AA	O1C[2]	0.90	2.06	2.941(4)	166.6
N1C	H1CA	O2D	0.90	2.10	2.905(4)	147.5
N1C	H1CB	O1A[3]	0.90	2.06	2.942(4)	167.6
N1D	H1DA	O1[4]	0.90	2.12	3.013(4)	170.6
N1D	H1DB	O2C	0.90	2.10	2.971(4)	164.0

Table S2. Hydrogen Bonds for **7a**

[1]-1/2+X,-1/2+Y,+Z; [2]1+X,+Y,+Z; [3]-1+X,+Y,+Z; [4]1/2+X,1/2+Y,+Z
